# Supplementary material for: Container Profiler: Profiling resource utilization of containerized big data pipelines
Source: Gigascience. 2023 Aug 25;12:giad069. doi: 10.1093/gigascience/giad069 (PMC10452954; doi:10.1093/gigascience/giad069)
Supplement: giad069_GIGA-D-23-00052_Original_Submission [file giad069_giga-d-23-00052_original_submission.pdf]

GigaScience

Container Profiler: Profiling Resource Utilization of Containerized Big Data Pipelines

--Manuscript Draft--

Manuscript Number:	GIGA-D-23-00052
Full Title:	Container Profiler: Profiling Resource Utilization of Containerized Big Data Pipelines
Article Type:	Technical Note
Funding Information:	National Institute of General Medical Sciences (R01GM126019) — Dr. Ka Yee Yeung<br>National Human Genome Research Institute (U24HG012674) — Dr. Ka Yee Yeung<br>Division of Microbiology and Infectious Diseases, National Institute of Allergy and Infectious Diseases (R03AI159286) — Dr. Ka Yee Yeung<br>Office of Advanced Cyberinfrastructure (1849970) — Dr. Wes Lloyd
Abstract:	Background This paper presents the Container Profiler, a software tool that measures and records the resource usage of any containerized task. Our tool profiles the CPU, memory, disk, and network utilization of containerized tasks collecting over fifty Linux operating system metrics at the virtual machine, container, and process levels. The Container Profiler supports performing time series profiling at a configurable sampling interval to enable continuous monitoring of the resources consumed by containerized tasks and pipelines.<br>Results To investigate the utility of the Container Profiler, we profile the resource utilization requirements of a multi-stage bioinformatics analytical pipeline (RNA sequencing using unique molecular identifiers). We examine profiling metrics to assess patterns of CPU, disk, and network resource utilization across the different stages of the pipeline. We also quantify the profiling overhead of our Container Profiler tool to assess the impact of profiling a running pipeline with different levels of profiling granularity verifying that impacts are negligible.<br>Conclusions The Container Profiler provides a useful tool that can be used to continuously monitor the resource consumption of long and complex containerized applications that run locally or on the cloud. This can help identify bottlenecks where more resources are needed to improve performance.
Corresponding Author:	Wes Lloyd<br>University of Washington, Tacoma<br>Tacoma, WA UNITED STATES
Corresponding Author Secondary Information:	
Corresponding Author's Institution:	University of Washington, Tacoma
Corresponding Author's Secondary Institution:	
First Author:	Varik Hoang
First Author Secondary Information:	
Order of Authors:	Varik Hoang<br>Ling-Hong Hung<br>David Perez<br>Huazeng Deng

Powered by Editorial Manager® and ProduXion Manager® from Aries Systems Corporation

	Raymond Schooley
	Niharika Arumilli
	Ka Yee Yeung
	Wes Lloyd
**Order of Authors Secondary Information:**	
**Additional Information:**	
**Question**	**Response**
Are you submitting this manuscript to a special series or article collection?	No
**Experimental design and statistics**<br><br>Full details of the experimental design and statistical methods used should be given in the Methods section, as detailed in our Minimum Standards Reporting Checklist. Information essential to interpreting the data presented should be made available in the figure legends.<br><br>Have you included all the information requested in your manuscript?	Yes
**Resources**<br><br>A description of all resources used, including antibodies, cell lines, animals and software tools, with enough information to allow them to be uniquely identified, should be included in the Methods section. Authors are strongly encouraged to cite Research Resource Identifiers (RRIDs) for antibodies, model organisms and tools, where possible.<br><br>Have you included the information requested as detailed in our Minimum Standards Reporting Checklist?	Yes
**Availability of data and materials**<br><br>All datasets and code on which the conclusions of the paper rely must be either included in your submission or	Yes

Powered by Editorial Manager® and ProduXion Manager® from Aries Systems Corporation

deposited in publicly available repositories (where available and ethically appropriate), referencing such data using a unique identifier in the references and in the “Availability of Data and Materials” section of your manuscript.<br><br>Have you have met the above requirement as detailed in our Minimum Standards Reporting Checklist?	

Manuscript
Click here to access/download;Manuscript;Profiler_Gigascience_Technical_

GigaScience, 2023, 1–11

doi: xx.xxxx/xxxx
Manuscript in Preparation
Paper

PAPER

Container Profiler: Profiling Resource Utilization of Containerized Big Data Pipelines

Varik Hoang[1,*], Ling-Hong Hung[1,*], David Perez[1], Huazeng Deng[1], Raymond Schooley[1], Niharika Arumilli[1], Ka Yee Yeung[1] and Wes Lloyd[1,†]

[1]School of Engineering and Technology, University of Washington Tacoma

[*]Contributed equally.
[†]wlloyd@uw.edu

Abstract

Background This paper presents the *Container Profiler*, a software tool that measures and records the resource usage of any containerized task. Our tool profiles the CPU, memory, disk, and network utilization of containerized tasks collecting over sixty Linux operating system metrics at the virtual machine, container, and process levels. The *Container Profiler* supports performing time series profiling at a configurable sampling interval to enable continuous monitoring of the resources consumed by containerized tasks and pipelines.
Results To investigate the utility of the *Container Profiler*, we profile the resource utilization requirements of a multi-stage bioinformatics analytical pipeline (RNA sequencing using unique molecular identifiers). We examine profiling metrics to assess patterns of CPU, disk, and network resource utilization across the different stages of the pipeline. We also quantify the profiling overhead of our Container Profiler tool to assess the impact of profiling a running pipeline with different levels of profiling granularity verifying that impacts are negligible.
Conclusions The *Container Profiler* provides a useful tool that can be used to continuously monitor the resource consumption of long and complex containerized applications that run locally or on the cloud. This can help identify bottlenecks where more resources are needed to improve performance.

Key words: Resource profiling; performance; testing; cloud computing; RNA sequencing

Findings

Background

Large-scale and diverse biomedical data have been generated to advance the understanding of biological mechanisms. Interpreting these data typically includes multiple analytical steps, each of which consists of different computational methods and software tools. An analytical *pipeline* (or *workflow*) is a sequence of computational tasks used to process and analyze specific biomedical data. Each analytical step in a pipeline can potentially require a different set of applications, libraries, and software dependencies. As a result, software containers that encapsulate executables with their dependencies have become popular to facilitate the deployment of complicated pipelines and to enhance their reproducibility [1, 2]. Additionally, different analytical steps in a pipeline could have different computing resource requirements. In particular, many bioinformatics pipelines consist of one or more computationally intensive steps stemming from their operation on large datasets requiring significant CPU, memory, network, and disk resources. As an example, the alignment step in a RNA sequencing pipeline typically requires relatively more CPU and memory resources than other steps, while the data download step typically requires more disk I/O and network resources.

Cloud computing has emerged as a solution that can provide the necessary resources needed for computationally intensive bioinformatics analyses [3, 4, 5, 6, 7, 8, 9]. However, deployment of analytical pipelines using Infrastructure-as-a-Service (IaaS) cloud platforms requires selecting the appropriate type

Compiled on: February 24, 2023.
Draft manuscript prepared by the author.

Key Points

- We present the *Container Profiler* a tool that enables profiling the resource utilization of any script or container-based task on Linux.
- The *Container Profiler* collects CPU, memory, disk, and network resource utilization metrics at the virtual machine, container, and process levels.
- The *Container Profiler* supports delta and time series resource utilization profiling at an adjustable time interval (e.g. 1-second) supporting monitoring and graphing of resource utilization enabling time series analysis to help identify performance bottlenecks for any Linux-based computational task.
- The *Container Profiler* can profile complex containerized computational jobs such as bioinformatics pipelines where multiple individual containers are used to implement specific steps.
- The *Container Profiler* is provided as a container which can merge with any existing container or used separately to profile independent Linux scripts or executables to characterize task resource utilization locally or on the cloud.
- We illustrate how different resources are required when performing different steps of a containerized pipeline analyzing unique molecular identifiers (UMI) RNA sequencing data.

and quantity of virtual machines (VMs) to address performance goals while balancing hosting costs. Cloud resource type selection is presently complicated by the rapidly growing number of available VM instance types and pricing models offered by public cloud providers. For example, the Amazon, Microsoft, and Google public clouds presently offer hundreds of different VM types under different pricing models. Further, Google allows users to create custom VM types with unique combinations of CPUs, memory, and disk capacity. These cloud VMs are available directly, or through various container platforms. Determining the best cloud deployment requires understanding the resource requirements of the pipeline.

Our Contributions

This paper presents the *Container Profiler*, a tool that supports profiling the computational resources utilized by software within a Docker container. Our tool is simple, easy-to-use, and can record the resource utilization for any Dockerized computational job. Understanding fine-grained resource utilization of containerized computational tasks can help identify resource bottlenecks and inform the choice of optimal cloud deployment. The *Container Profiler* collects over sixty metrics to characterize the CPU, memory, disk, and network resource utilization at the VM, container, and process level. In addition, the *Container Profiler* supports time-series graphing enabling the visualization and monitoring of resource utilization of containerized tasks and pipelines.

We present a case study involving profiling the resource utilization of a multi-stage containerized bioinformatics pipeline that analyzes the unique molecular identifiers (UMI) of RNA sequencing data. In this study, we demonstrate how the *Container Profiler* performed time-series sampling at a one-second interval while a compute-bound bioinformatics pipeline simultaneously ran up to 85 distinct processes. Under load, our tool was able to profile the RNA-sequencing pipeline with full verbosity (all metrics) with 100% of the profiling samples obtained in under 100ms.

Related Work

Cloud computing has been used to process massive RNA sequencing (RNA-seq) datasets [10, 11]. These pipelines typically consist of multiple computational tasks, where not all tasks necessarily have the same resource requirements. Tatlow *et al.* studied the performance and cost profiles for processing large-scale RNA-seq data using pre-emptible virtual machines (VMs) on the Google Cloud Platform [10]. The authors collected resource utilization metrics to characterize user and system vCPU utilization, memory usage, disk activity, and network activity for the different computational stages of the RNA-seq pipeline. Tatlow *et al.* observed how resource utilization can vary dramatically across different processing tasks in the pipeline, while demonstrating that resource profiling can help to identify resource requirements of unique pipeline stages. Juve *et al.* developed a pair of tools called wfprof (pipeline profiling) to collect and summarize performance metrics for diverse scientific pipelines from multiple domains including bioinformatics [12]. Wfprof consists of two tools, ioprof to measure process I/O, and pprof that characterizes process runtime, memory usage, and CPU utilization. These tools accomplish profiling at the machine level primarily by analyzing process level resource utilization, and they do not focus on profiling containerized pipelines, nor do they collect container specific metrics.

Tyryshkina, Coraor, and Nekrutenko leveraged coarse grained resource utilization data from historical job runs collected over 5 years on the Galaxy platform to estimate the required CPU time and memory to improve task scheduling [13]. Galaxy, a scientific workflow, data integration, data analysis, persistence, and publishing platform was initially developed for genomics research and is now considered largely domain agnostic and is used for processing general bioinformatics pipelines. The authors identified the challenge of determining the appropriate amount of memory and processing resources for scheduling bioinformatics analyses at scale. The majority of metrics in the study consisted of metadata regarding job configurations. Assessing the utility of using fine grained operating system metrics as with the Container Profiler to profile resource utilization of genomics pipelines was not the focus. This effort considered many older jobs that ran on Galaxy where containers were not used thus they lacked container based metrics.

Outside bioinformatics, Weingartner et al. highlight the importance of profiling resource requirements of applications for deployment in the cloud to improve resource allocation and forecast performance [14]. Brendan Gregg described the USE method (Utilization, Saturation, and Errors) as a tool to diagnose performance bottlenecks [15]. Gregg's method involves checking utilization of every resource involved in the system including CPUs, disks, memory, and more to identify saturation and errors. Lloyd *et al.* provided a virtual machine manager known as VM-scaler that integrated resource utilization profiling of software deployments to Infrastructure-as-a-Service (IaaS) cloud VMs [16]. VM-scaler focused on the management and profiling of cloud infrastructure used to host environmental modeling web services. This work was later extended by

building resource utilization models to enable identifying the most cost effective cloud VM types to host environmental modeling web service workloads without sacrificing runtime or throughput [17]. This effort demonstrated a cost variance of 25% for hosting these workloads across different VM types on the Amazon Elastic Compute Cloud (EC2) while identifying potential for cost savings up to $25,000 for 10,000 hours of compute time.

To characterize resource requirements of containerized tasks and pipelines, a variety of commercial and open source tools exist. The vast majority of the available tools, however, require the setup and maintenance of a complete monitoring application including a time-series database and web application server. [18] These monitoring applications require dedicated infrastructure (i.e. servers and/or virtual machines) to run always-on daemons. Many of these tools are also oriented towards monitoring entire container clusters (e.g. Kubernetes). Access to such cluster-level monitoring tools is often restricted organizationally to system administrators and privileged users and not made freely available to any user. For container profiling, there are far fewer solutions that enable a user to easily profile the resource utilization of containerized tasks or pipelines on a local computer or personal cloud VM with minimal effort and expertise. The lack of lightweight easy-to-use developer tools that require no setup or maintenance of a permanent monitoring application and/or database server is what motivated the creation of the *Container Profiler*.

CMonitor as a related tool has been developed to support similar goals of lightweight container profiling without setup of a full monitoring application [19, 20]. CMonitor is installed and run on the host and is used to profile host metrics in addition to container metrics as the tool is not focused specifically on profiling a containerized task or pipeline. CMonitor, however, runs as an external tool which requires the user to posses detailed information about the host's operating system, runtime configuration, and Docker setup. Additionally CMonitor does not support container profiling of ARM-based Linux VMs or servers. These systems are of interest with the advent of low-cost compute-optimized VMs based on the Graviton series of ARM CPUs (e.g. c6g and c7g) on Amazon EC2 [21, 22, 23]. These VMs offer performance improvements and cost savings of interest for executing bioinformatics pipelines. CMonitor is installed as a package requiring several dependencies.

Container Profiler: Overview

The *Container Profiler* tool supports profiling resource utilization including CPU, memory, disk, and network metrics of containerized tasks. Resource utilization metrics are obtained across three levels: virtual machine (VM)/host, container, and process. Our implementation leverages facilities provided by the Linux operating system that is integral with Docker containers. Development and testing of the *Container Profiler* described in this paper was completed using Debian-based Ubuntu Linux.

The *Container Profiler* collects information from the Linux `/proc` and `/sys/fs/cgroup` file systems while a workload is running inside a container on the host machine. To support collecting metrics the *Container Profiler* is implemented using Python3 while leveraging psutil, a cross-platform library for retrieving information on running processes and system utilization [24]. It should be noted that psutil itself is not a profiling tool. Psutil assists with collecting host-level and process-level metrics from the system, but does not process metrics for time-series analysis or graphing. Psutil also does not output metrics in specific formats (e.g. JSON, CSV) or orchestrate time-series profiling. The host machine could be a physical computer such as a laptop or a virtual machine (VM) in the public cloud. The workload being profiled can be any job capable of running inside a Docker container. Figure 1 provides an overview of the various metrics collected by the *Container Profiler*.

Host-Level Metrics: Host/VM level resource utilization metrics are obtained from the Linux `/proc` virtual filesystem using psutil. The `/proc` filesystem is a virtual filesystem that consists of dynamically generated files produced on demand by the Linux operating system kernel providing an immense amount of data regarding the state of the system [25]. Files in the `/proc` filesystem are generated at access time from metadata maintained by Linux to describe current resource utilization, devices, and hardware configuration as managed by the Linux kernel. The *Container Profiler* queries the `/proc` filesystem directly and by using the psutil library at regular time intervals to obtain resource utilization metrics. Documentation regarding the Linux `/proc` filesystem is found on the `/proc` Linux manual pages [25] though other references provide more detailed descriptions of available metadata: [26, 27, 28, 29, 30, 31, 32, 33, 34, 35, 36]. User-mode and kernel-mode CPU utilization metrics can be obtained found in the `/proc/stat` file. Table 1 provides a subset of CPU, disk, and network utilization metrics profiled by the *Container Profiler* at the VM/host level.

Container-Level Metrics: Docker relies on the Linux `cgroup` and `namespace` features to facilitate the aggregation of a set of Linux processes together to form a container. `Cgroups` were originally added to the Linux operating system to provide system administrators with the ability to dynamically control hardware resources for a set of related Linux processes [37]. Linux control groups (`cgroups`) provide a kernel feature to both limit and monitor total resource utilization of containers. Docker leverages `cgroups` for resource management to restrict hardware access to the underlying host machine to facilitate sharing when multiple containers share the host. Linux subsystems such as CPU and memory are attached to a `cgroup` enabling the ability to control resources of the `cgroup`. Resource utilization of `cgroup` processes is aggregated for reporting purposes under the `/sys/fs/cgroup` virtual filesystem and we leverage this filesystem to obtain container-level metrics in the *Container Profiler*. `Cgroup` files provide aggregated resource utilization statistics describing all of the processes inside a container. Container-level metrics are not available from psutil. As a profiling example, a container's CPU utilization statistics can be obtained from `/sys/fs/cgroups/cpuacct/cpuacct.stat`. Table 2 describes a subset of the CPU, disk, and network utilization metrics profiled at the container level by the *Container Profiler*.

Process-Level Metrics: The *Container Profiler* also supports profiling the resource utilization for each process running inside a container. The *Container Profiler* leverages support from the psutil library to capture process level metrics from Linux. Table 3 describes a subset of the process-level metrics collected by the *Container Profiler* to profile resource utilization of container processes.

Resource utilization data collected at the VM/host, container, and process level allows characterization of resource use with increasingly greater isolation. Host-level resource metrics for example, do not isolate background processes. This could lead to variance in measurements as background processes on the host machine outside the container may be randomly present. Profiling at the container level allows fine-grained resource profiling of ONLY the resources used by the containerized task or pipeline. Finally, profiling at the process level allows very fine-grained profiling so that resource bottlenecks can be attributed to the specific activities or tasks. The ability of the *Container Profiler* to characterize resource utilization at multiple levels enables high observability of the resource requirements of computational tasks. This observability can be crucial to improving job deployments to cloud platforms to al-

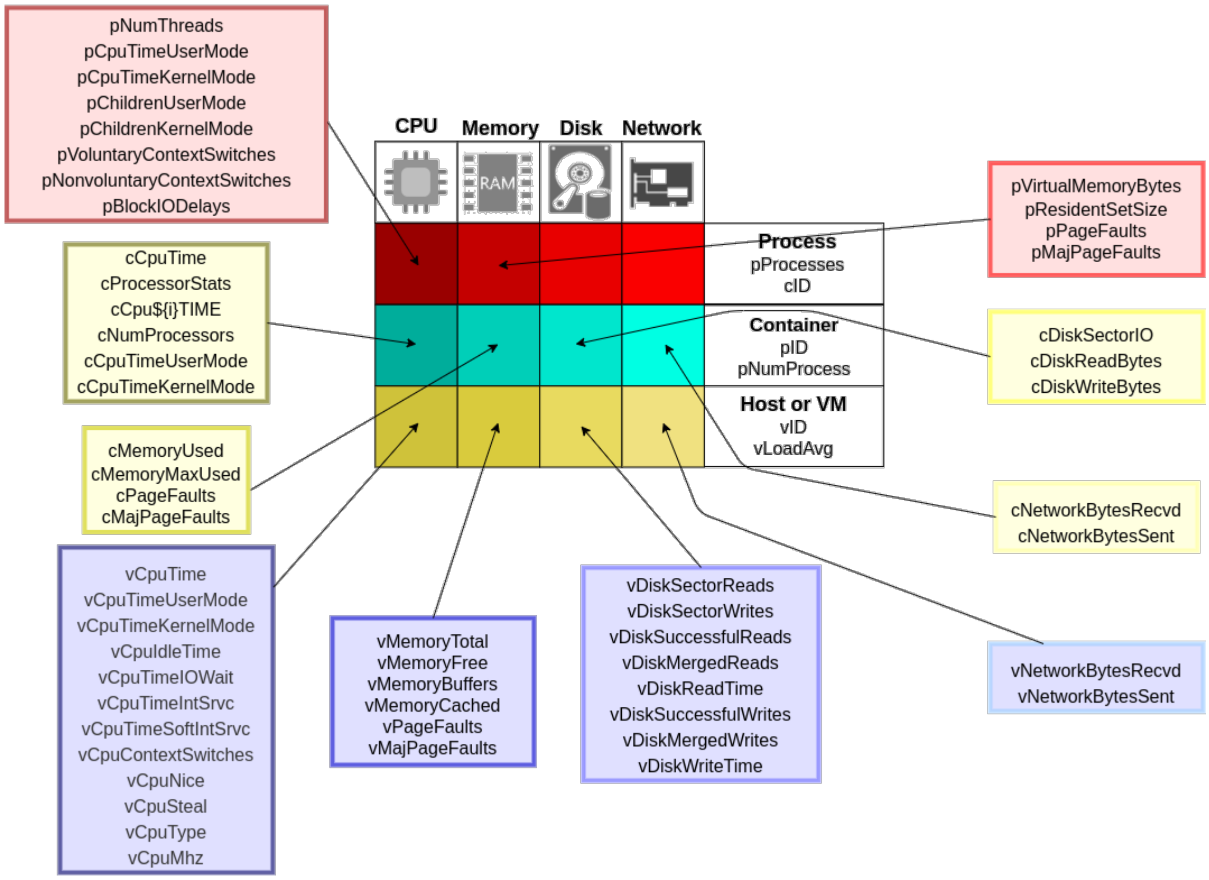

Figure 1. Overview summarizing resource utilization metrics (61 total) collected by the *Container Profiler* across three levels (i.e. host/VM, container, and process level) and four categories (i.e. CPU, memory, network, and disk). Process level metrics are depicted by red and prefaced with lower case "p", container level metrics by yellow and prefaced with lower case "c", and host/VM level metrics by blue and prefaced with lower case "v".

Table 1. Selected CPU, disk, and network utilization metrics profiled at the VM/host level.

Metric	Description	Source
vCpuTimeUserMode	Time the CPU spent executing in user mode	/proc/stat
vCpuTimeKernelMode	Time the CPU spent executing in kernel mode	/proc/stat
vCpuIdleTime	Time the CPU was idle	/proc/stat
vCpuTimeIOWait	Time the CPU waits for I/O to complete	/proc/stat
vCpuContextSwitches	The total number of context switches across all CPU cores	/proc/stat
vDiskSectorReads	Number of sector reads	/proc/diskstats
vDiskSectorWrites	Number of sectors writes	/proc/diskstats
vDiskReadTime	Time spent reading	/proc/diskstats
vDiskWriteTime	Time spent writing	/proc/diskstats
vNetworkBytesRecvd	Network Bytes received	/proc/net/dev
vNetworkBytesSent	Network Bytes written	/proc/net/dev

leviate performance bottlenecks and optimize performance and cost.

Results

We demonstrate the Container Profiler using unique molecular identifier (UMI) RNA sequencing data generated by the LINCS Drug Toxicity Signature (DToxS) Generation Center at Icahn School of Medicine at Mount Sinai in New York [38]. The scripts and supporting files for the analytical pipeline to analyse this originated from the Broad Institute [39]. In addition to downloading the datasets, there are 3 other stages. The first stage is a demultiplexing or split step that sorts the reads using a sequence barcode to identify the originating sample. The second stage aligns the reads to a human reference sequence to identify the gene that produced the transcript. The final stage is the "merge" step which counts all the aligned reads to identify the number of transcripts produced by each gene. The unique molecular identifier (UMI) sequence is used to filter out reads that arise from duplication during the sample preparation process. In the original pipeline, only the most CPU intensive part of the pipeline, the alignment step, was optimized and executed in parallel. We further optimized the split and align steps in the original pipeline [39] to decrease the running time from 29 to 3.5 hours in our previous work [40]. We also encapsu-

Table 2. Selected CPU, disk, and network utilization metrics profiled at the container level.

Metric	Description	Source
cCpuTimeUserMode	CPU time consumed by tasks in user mode	/sys/fs/cgroup/cpuacct/cpuacct.stat
cCpuTimeKernelMode	CPU time consumed by tasks in kernel mode	/sys/fs/cgroup/cpuacct/cpuacct.stat
cDiskSectorIO	Number of sectors transferred to or from specific devices	/sys/fs/cgroup/blkio/blkio.sectors
cDiskReadBytes	Number of bytes transferred from specific devices	/sys/fs/cgroup/blkio/blkio.throttle.io_service_bytes
cDiskWriteBytes	Number of bytes transferred to specific devices	/sys/fs/cgroup/blkio/blkio.throttle.io_service_bytes
cNetworkBytesRecvd	The number of bytes each interface has received	/proc/net/dev
cNetworkBytesSent	The number of bytes each interface has sent	/proc/net/dev

Table 3. List of important metrics for profiling process resource utilization.

Metric	Description	Source
pCpuTimeUserMode	Amount of time that this process has been scheduled in user mode	/proc/[pid]/stat
pCpuTimeKernelMode	Amount of time that this process has been scheduled in kernel mode	/proc/[pid]/stat
pVoluntaryContextSwitches	Number of voluntary context switches	/proc/[pid]/status
pNonvoluntaryContextSwitches	Number of involuntary context switches	/proc/[pid]/status
pBlockIODelays	Aggregated block I/O delays	/proc/[pid]/stat
pResidentSetSize	Number of pages the process has in real memory	/proc/[pid]/stat

lated each step in the pipeline in separate Docker containers to facilitate deployment and ensure reproducibility.

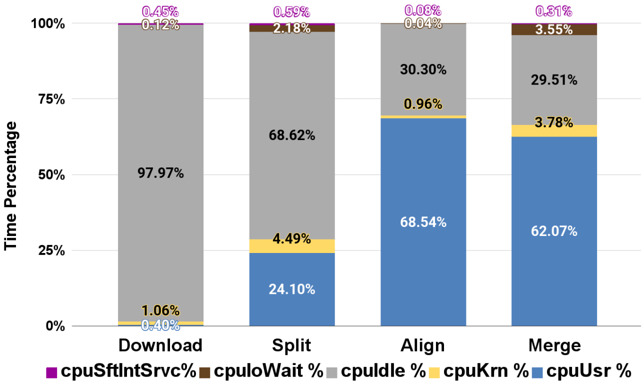

Figure 2. CPU utilization graph for the four stages (e.g. download, split, align, and merge) of the UMI RNA-seq pipeline. This graph depicts the percentage of CPU utilization in each CPU mode. CpuUsr (shown in green) captures time the pipeline spent executing its source code. CpuKrn (shown in yellow) captures time when the processor executed code in the Linux kernel. Typically the kernel is invoked to support disk and network I/O which are considered privileged operations. CpuIdle (shown in blue) is unused time across the 8 available CPU cores throughout each stage. CpuIdle time is common when waiting for disk or network I/O to complete. High CpuIdle time during computational stages indicates potential for performance optimization with better parallelization of code. CpuIOWait (shown in maroon) depicts CPU time where the pipeline was waiting for I/O (disk or network) to complete. cpuSftIntSrvc (shown in magneta) is time spent handling soft interrupts. Soft interrupts commonly occur with network I/O.

To profile resource utilization, we deployed our UMI RNA-sequencing pipeline alongside the *Container Profiler* on an IBM Cloud bx2d-metal-96x384 virtual machine with dual Intel Platinum 8260 CPUs at 2.4 GHz, with 96 virtual CPU cores, 384GB of memory, and a 960 GB SATA M.2 mirrored SSD as the local boot disk. We leveraged the UMI RNA-sequencing pipeline as our case study as each stage of the RNA-seq pipeline exhibits different resource utilization characteristics. Specifically, the dataset download stage is limited by the network capacity. The split stage writes many files and is limited by the speed of disk writes. The alignment stage is performed by multiple CPU-intensive processes and performance is primarily limited by the CPU. However, it is possible that available memory capacity will limit the performance in some circumstances. The final merge stage involves reading many files in parallel, consuming both memory and CPU resources depending on the number of threads used.

Container Profiler can inform pipeline optimization

Figure 2 summarizes the CPU utilization characteristics of different stages of the UMI RNA-seq pipeline. The CPU usage profile is consistent with our expectations. The execution of the align and merge steps are expected to be bound by CPU resources and they indeed spent the majority of the time executing source code. Download is limited by the network bandwidth and the split stage by disk I/O. Hence the cpuIdle time is highest in these stages.

Despite the fact that the align stage is expected to be limited by the CPU resources, there is significant CPU-idle time during that stage. This suggests the presence of a bottleneck that may be the target for further optimization. We collected CPU, memory, network, and disk utilization metrics at both the container and VM/host levels for the RNA sequencing analytical pipeline. These are visualized in Figure 3. Note that the x-axis depicting time in this figure encompasses the entire pipeline incorporating all stages: download, split, align, and merge. Overall our profiling results depict resource utilization patterns that we expected. The download stage consumes network resources. The split stage is the most disk intensive step. The alignment and merge stages consume the most CPU resources. Our profiling data also points to areas where resource consumption may be a problem. For example, memory usage is high for all the stages. This may be due to greedy allocation by the executables, or it may indicate that allocating more memory could benefit the pipeline. Most interesting, is CPU utilization during the alignment stage. Just before the 3 hour mark, we see a series of drops over the next 30 minutes, creating a ladder of 8 steps. The alignment stage uses up to 8 vCPUs to align different files of reads simultaneously. Near the end of the alignment stage,

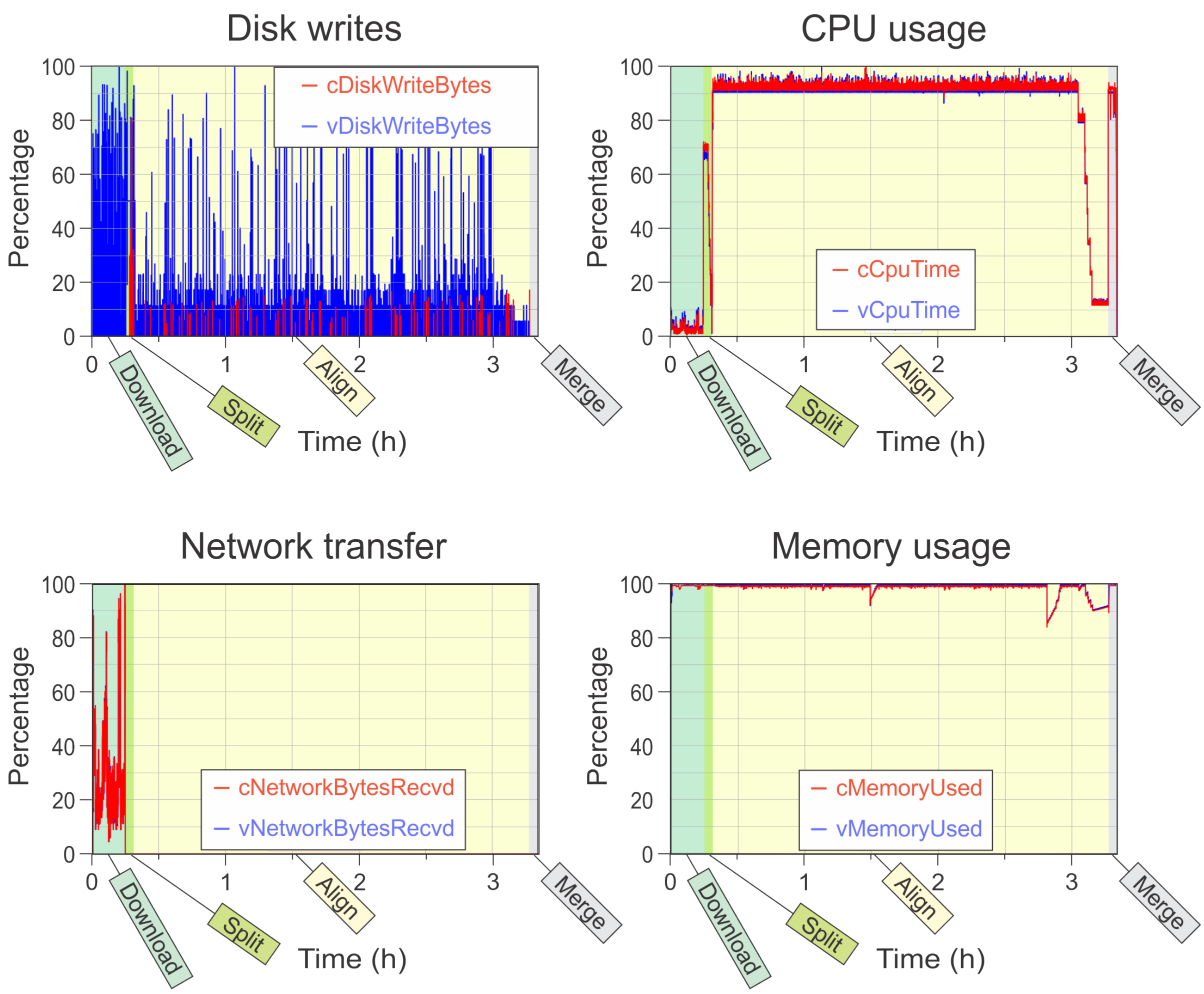

Figure 3. Output graphs comparing Container and VM (host) level metrics over time for a multi-stage RNA sequencing data pipeline. Four output graphs are shown: disk writes (top left), CPU usage (top right), network usage (bottom left) and memory usage (bottom right). In each graph, the container level metrics are shown in red and the VM (host) level metrics are shown in blue. For disk usage and memory usage, the native host metric was transformed to have the same units as the container metric. All metrics have been transformed to the same units and scaled as a percentage of the maximum observed value. The four stages of the pipeline include downloading the data (download), splitting and demultiplexing the reads (split), aligning the reads to the reference (align), and assembling the counts while removing duplicate reads (merge). We observe that the container and VM-level metrics mostly overlap in the stages. However, there are differences when there are background processes, most notably when there is considerable disk usage. The alignment stage is also notable in that we can see that the CPU usage declines near the end, probably indicating that the pipeline is waiting on some slower threads (i.e. stragglers) to finish before it can proceed, indicating this stage might be improved with better load balancing, or with smaller workloads for the threads. This is an example of how the *Container Profiler* can be used to identify portions of the pipeline that can be optimized.

most of the files will have been processed and there will be more available vCPUs than unprocessed files. As a result, the CPU utilization drops as vCPUs lie idle waiting for the final files to be processed. However, this under-utilization of resources lasts for 30 minutes indicating that the final files are rather large. This presents an opportunity to improve pipeline performance by splitting the processing into smaller files (which is an option in the split software), or by processing the largest files first. We would not have known about these potential optimizations without fine-grained profiling results from the *Container Profiler*.

Container-level metrics can provide useful additional information

A key feature of the *Container Profiler* is the ability to capture container-level metrics to describe resource utilization of only the containerized task(s). We expect these metrics to be similar, and that they could differ given that the VM/host level metrics also encompass resources being used by processes running on the host external to the container and pipeline. Since we only ran our pipeline on an dedicated test VM, the container metrics should be very similar to the VM/host metrics, which was in fact the case from our observations. However, one can see differences between the disk utilization metrics during the split and alignment stages where there are a large number of disk writes to the host file system. Docker manages these disk writes by providing the container with an internal mount point which is eventually written to a host file. The caching and management of this data is external to the container and is not captured by the container metrics, but is captured by the host metric. In addition, during the alignment stage, intermediate results from the aligner are continuously piped to another process which then re-formats the intermediate output and writes the final output to a file on the host system. Multiple threads are used, more than the available number of cores resulting in frequent context switches. The pipe management and context-switching are also handled by the operating system and are captured by the host metric and not the container metrics. The separation of container and OS based consumption can be useful for example, when trying to assess effects due to resource contention that may occur when multiple jobs are run on the same physical host, which often happens on

public clouds where the assignment of instances to hosts is controlled by the vendor.

Container Profiler can sample container and host metrics with sub-second resolution

For the *Container Profiler* to be useful, the collection of profiling metrics must have sufficiently low overhead to enable rapid sampling of resource utilization to collect many samples for time series analysis. The time required to collect the metrics limits the granularity of the profile. To achieve 1 second sampling for time series analysis requires the ability to repeatedly sample resource utilization every 1 second (1000 ms). However, profiling time is not constant, and depends on the state of resources being utilized by the containerized pipeline and the host. The variability of profiling time is shown in the histogram in Figure 4. When profiling our RNA-sequencing pipeline, VM-level and container-level profiling had a bi-modal distribution, while process-level sampling had a tri-modal distribution. The slowest profiling was observed during the stressful compute-bound alignment stage of the pipeline. For all levels of profiling verbosity, the *Container Profiler* was able to profile resource utilization in less than 100ms. The longest profiling time and highest variation was for process-level profiling as metrics are collected for each process in the pipeline. The number of processes can vary throughout the execution of complex parallel pipelines, as was the case for the align stage of our RNA-sequencing pipeline. Our RNA-sequencing pipeline featured a maximum of 85 concurrent processes during the align stage. These processes ran for approximately 39% of the duration of the align stage. The time required to capture host and container level metrics was less variable as the number of metrics collected is fixed. As shown in Figure 4, 90% of the time, the container and host level metrics were collected in less than 63 milliseconds and always under 75 milliseconds. The process metrics do take longer to collect but still less than 100 milliseconds in the worst case. Profiling at the process-level involves collecting all metrics every second. For profiling our UMI RNA-sequencing pipeline use case which required 2.5 hours to execute with one-second sampling and full profiling verbosity (process-level metrics), 9,000 JSON files were collected which required 296 MB of storage space.

Container Profiler has lower overhead than the variation in pipeline execution time on public clouds

A design objective for the *Container Profiler* is to not significantly impact the performance of the pipeline being profiled. Failing to realize this objective may result in the overhead from resource profiling impacting the collected metrics. While some overhead is unavoidable, ideally it should be lower than the inherent variations of pipeline execution time on the public cloud.

To measure the performance impact of resource utilization profiling when running the RNA-seq pipeline, we initially attempted to assess the overhead using Amazon Elastic Compute Cloud (EC2) cloud VMs. However, we discovered that the runtime of the RNA-seq pipeline varied by more than 5% on Amazon EC2, which was more than 5x greater than the overhead of the *Container Profiler*. This degree of performance variance made it difficult to evaluate the performance overhead of the *Container Profiler* since we could not easily distinguish between pipeline performance variance and profiling overhead on EC2. We then measured the performance overhead of the *Container Profiler* by profiling the pipeline using the IBM cloud bx2d-metal-96x384 server which had performance variance around

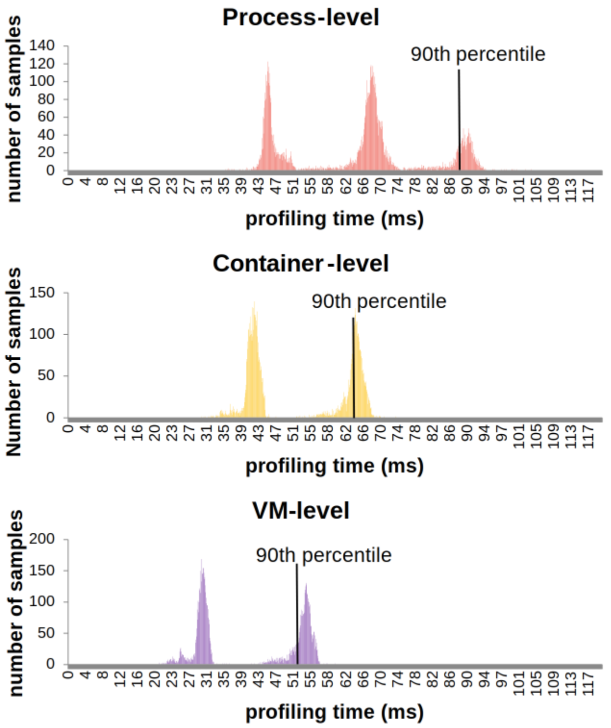

Figure 4. Distribution plot (log-scale) of time required to collect profiling data. We profiled resource utilization of the RNA-sequencing pipeline on an IBM Cloud bx2d-metal-96x384 virtual machine with dual Intel Platinum 8260 CPUs at 2.4 GHz, with 96 virtual CPU cores, 384GB of memory, and a 960 GB SATA M.2 mirrored SSD as the local boot disk). We executed the complete RNA-seq pipeline four times to profile 1) only VM/host metrics, 2) VM/host and container metrics, 3) *ALL* metrics, and no metrics by running the pipeline in the absence of the profiler. Plots depict time to collect resource utilization samples at one-second intervals with the Container Profiler while running the entire RNA-seq pipeline. Time to collect 9000 samples of each type (Process-level, Container-level, and VM-level) is shown. 99.95% of process-level samples were collected under 100 milliseconds, while all container-level samples were collected under 74 milliseconds, and all VM-level samples were collected at or under 60 milliseconds. The figure shows the process-level, container-level, and VM-level profiling time distribution over 120 milliseconds on the x-axis. The 90th percentiles for sample collection are shown.

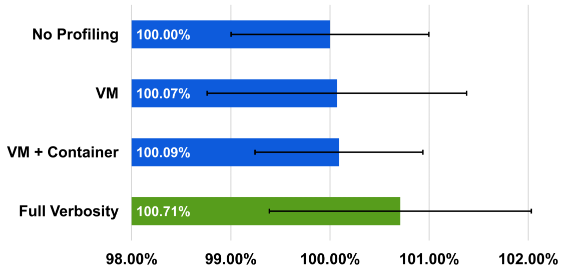

Figure 5. This figure depicts the profiling overhead of the *Container Profiler* and the resulting percentage increase in the total runtime of the entire RNA-seq pipeline. The increases in run time are very modest: Host/VM only (0.07%), Host/VM + Container (0.09%), and Host/VM + Container + Process (0.71%). Error bars depict one standard deviation from the average. Standard deviation of pipeline runtime for 5 runs of the RNA-seq pipeline on the IBM bx2d-16x64 Virtual Machine with no profiling was (1.38%), approximately 194% greater than the worst case overhead of the Container Profiler when profiling with full verbosity (i.e. collecting all metrics).

1%. Figure 5 depicts the overhead from one-second resource utilization sampling by the *Container Profiler* for the RNA-seq pipeline on the IBM metal server. IBM metal servers are private and not shared with multiple users. Running on this isolated

server greatly reduced the performance variance of running RNA-seq. We measured worst case overhead for the *Container Profiler* to be 0.71%, which equates to about 3.4 minutes for an 8-hour pipeline with full verbosity metrics collection (VM + container + process). Overhead is reduced to as little as .07% overhead, or about 20 seconds for an 8-hour pipeline when only collecting VM-level metrics. Adding container-level, and especially process-level metrics slightly increased the runtime of the RNA-seq pipeline for collecting resource utilization data. We believe that this profiling overhead is within an acceptable level and note that even at maximum profiling verbosity, it is substantially less than the observed performance variance for running our RNA-seq pipeline on a public cloud VM. Users can reflect on our reported overhead times to make informed decisions when planning to profile their own pipelines.

Methods

Implementation Details

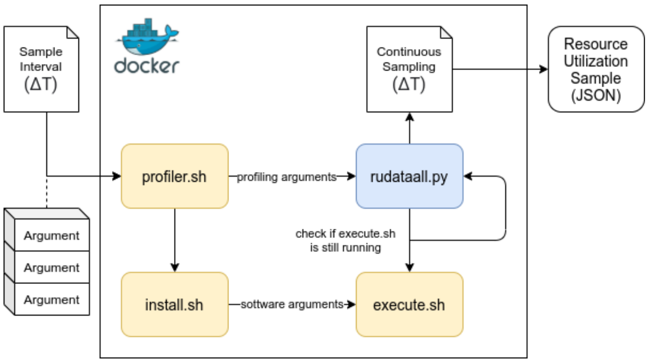

Figure 6. Profiling scripts used in the implementation of Container Profiler. All scripts are deployed inside the container alongside the software being profiled. The software to be profiled can be installed using an install script, or the Container Profiler can be installed on top of the original container image. The user provides a sampling interval and profiling arguments to initialize profiling.

The *Container Profiler* is implemented as a collection of Bash and Python scripts. Figure 6 provides an overview. There are three basic use cases for building a docker image for the Container Profiler. The first use case allows users to profile an existing Docker container by providing a Dockerfile which specifies their own setup and software installation inside the container. This is the simplest approach to profiling when the user has a working Dockerfile. The other two use cases support users who do not know how to write Docker files but are familiar with writing Bash scripts. The second use case gives users the ability to install all software inside the Docker image when the software installation becomes too complicated to put in the Dockerfile. In other words, it puts the required installation commands into a script that will be executed by the Dockerfile. For the third use case, the user provides their own executable bash script as the entry point in the Docker container. This use case can help the user simplify a set of commands they have to profile. In this case, the user just puts a set of commands into an executable script file and runs it as the entrypoint of the container. When the *Container Profiler* is executed inside a Docker container, it snapshots the resource utilization for the host (i.e. VM), container, and all processes running inside the container producing output statistics to a `.json` file. A sampling interval (e.g. once per second) is specified to configure how often resource utilization data is collected to support time series analysis of containerized applications and pipelines. Time series data can be used to train mathematical models to predict the runtime or resource requirements of applications and pipelines. Time series data can be visualized by using matplotlib Python graphing scripts that are included with the *Container Profiler*.

To improve the periodicity of time series sampling, we continuously subtract the most recent observed run time of the *Container Profiler* for sample collection from the configured sampling interval (e.g. 1 second) in `rudataall.py`. This approach notably improved the periodicity of sampling when the container was under load improving our ability to obtain samples at evenly spaced intervals. To enable addressing any potential drift of sample collection times, we capture timestamps for when each resource utilization metric is sampled in the output JSON. These timer ticks enable precise calculation of the time that transpires between resource utilization samples for each metric. This allows the rate of consumption of system resources (e.g. CPU, memory, disk/network I/O) to be precisely determined throughout the pipeline's execution. The *Container Profiler* consists of the profiling script and two supporting scripts (for installation and pipeline execution) depicted in Figure 6: `profiler.sh`, `install.sh`, and `execute.sh`.

The `profiler.sh` script is the primary script that generates profiling information in JSON format describing resource utilization of the containerized task. The `profiler.sh` script requires the user to provide a command or a set of commands along with arguments to start the profiling. This script internally invokes another Python script `rudataall.py`.

The `rudataall.py` script collects the resource utilization data. Specifically, this script takes a snapshot of the resource utilization metrics and records output to a JSON file using the time of the sample as a unique filename. The script accepts parameters `-v`, `-c`, and `-p` to inform the tool what type of data to collect: VM, container, and/or processlevel metrics respectively. The default behavior when running this script without any parameters is to collect all metrics.

The `profiler.sh` script only works if the workflow/software is already installed in the containerized environment. This means that we cannot profile workflows/software that has not been containerized. The *Container Profiler* provides an option that enables users to install software in a container using the `install.sh` script. Users provide a set of commands in the `install.sh` script to install their dependencies and software they wish to profile. Once installed, the user can run the `profiler.sh` script against the newly installed software. To profile resource utilization of a bash script, users can specify a series of commands using the optional `execute.sh` script to configure profiling.

Some users may be more familiar with editing Dockerfiles instead of bash scripts. We provide support for users to provide their own Dockerfile to build a custom container to be profiled.

Technical details using our scripts

To use the *Container Profiler* scripts with any container, a Linux based Docker container that encapsulates a script or job to run inside is required. To configure the *Container Profiler* tool to profile the container, users can optionally provide an executable script inside the *Container Profiler* which is specified during the `build.sh` script. In the executable script, the user launches the container's job or task to be profiled.

The `profiler.sh` script has four different modes: `profile`, `delta`, `csv`, and `graph`:

For the `profile` mode, there are two required parameters: the output directory specifies the location of generated profil-

Table 4. Container Profiler with four different modes

Mode	Description
profile	profile resource utilization
delta	calculate the different between two profiling samples
csv	convert a set of JSON resource utilization files into a single CSV file
graph	generate profiling graph(s) from a CSV file

ing files in JSON format, and the time interval specifies a time series sampling interval in milliseconds. The profiler generates a JSON file at the beginning and the end of the process if the sampling interval is set to zero. Otherwise, the profiler generates a JSON file at each sampling interval. The Container Profile also collects static metrics which typically describe hardware characteristics. The profiler first checks if a static information file exists (static.json). If missing, the profiler captures static parameters and writes out the static information file at the start of profiling. By default 11 static metrics are captured. They include: the host's kernel info, the host's cpu type, CPU Level 1 instruction cache size, CPU Level 1 data cache size, CPU Level 2 cache size, CPU Level 3 cache size, host boot time, host VM ID, the number of CPU cores available to the container, and the container ID.

For the `delta` mode, there are two required parameters: the input directory which contains the original raw JSON files, and the output directory where the delta JSON files will be written. The `delta| mode` also provides an option to allow users to specify the modification operator for performing the delta. The default delta operator calculates the difference between two samples (i.e. final minus initial value). The typical use case is to calculate the delta of the resource utilization between the first and last sample to capture the full resource utilization of a task or pipeline. Other operators include max, min, and average to determine the max, min, and average values of metrics from a set of JSON files.

For the `csv` mode, there are two required parameters: the input directory that contains processed JSON files in delta format, and the name of an output CSV file where all resource utilization data from the processed JSON files will be aggregated to.

For the `graph` mode, there are two required parameters: the input CSV file capturing all resource utilization data from processed JSON files, and the output directory for writing graph files. In addition, there are a few other options such as one to specify whether to plot the curves together or using separate graph files.

Visualization

The *Container Profiler* in the `graph` mode also provides an option to specify the creation of time-series graphs. The graphing configuration file supports multiple settings to specify how to generate graph(s). Each graph configuration file should start with a line that includes the components: the `###` followed by the title and the y-coordinate label. This is followed by line(s) that describe the metric(s) that users want to output in a single graph (one metric per line). As a starting point, a default graph configuration file `graph.cfg` is provided in the `cfg` directory.

Availability of supporting data and materials

- Project name: Container Profiler
- Project home page: `https://github.com/wlloyduw/ContainerProfiler`
- Contents available for download: Docker Images, Dockerfiles, installation scripts, and execution scripts.
- Operating system(s): Linux, Mac OS X.
- Programming language(s): Python, Bash
- License: MIT License

Declarations

List of abbreviations

AWS: Amazon Web Services; EC2: Elastic Compute Cloud; VM: virtual machine; CPU: central processing unit; IaaS: Infrastructure-as-a-Service; RNAseq: RNA sequencing; LINCS: Library of Integrated Network-Based Cellular Signatures; DToxS: Drug Toxicity Signature; RNA: ribonucleic acid; cgroup: container control group.

Consent for publication

Not applicable.

Competing Interests

LHH and KYY have equity interest in Biodepot LLC, which receives compensation from NCI SBIR contract numbers 75N91020C00009 and 75N91021C00022. The terms of this arrangement have been reviewed and approved by the University of Washington in accordance with its policies governing outside work and financial conflicts of interest in research.

Author's Contributions

VH, LHH, HD, RS, and DP contributed to the development of the Container Profiler. LHH implemented Docker containers for RNA-seq pipelines. VH, RS, NA, and DP conducted performance testing and empirical experiments. KYY, RS, WL, VH, and LHH drafted the manuscript. WL, KYY, and LHH designed the case study. WL provided cloud computing expertise. WL and KYY coordinated the benchmarking experiments. All authors edited the manuscript.

Acknowledgements

LHH, HD, RS, WL, and KYY are supported by the National Institutes of Health (NIH) grant R01GM126019. DP is supported by the NIH Diversity Supplement R01GM126019-02S2. LHH and KYY are also supported by NIH grants U24HG012674 and R03AI159286. WL is also supported by NSF grant OAC-1849970. We acknowledge support from the AWS Cloud Credits for Research and IBM Cloud Credits (awarded to LHH, WL, and KYY).

Supplementary Information

References

1. O'Connor BD, Yuen D, Chung V, Duncan AG, Liu XK, Patricia J, et al. The Dockstore: enabling modular, community-focused sharing of Docker-based genomics tools and workflows. F1000Research 2017;6.
2. da Veiga Leprevost F, Grüning BA, Alves Aflitos S, Röst HL, Uszkoreit J, Barsnes H, et al. BioContainers: an open-source and community-driven framework for software standardization. Bioinformatics 2017;33(16):2580–2582.
3. Dai L, Gao X, Guo Y, Xiao J, Zhang Z. Bioinformatics clouds for big data manipulation. Biology direct 2012;7(1):43.
4. Schadt EE, Linderman MD, Sorenson J, Lee L, Nolan GP. Computational solutions to large-scale data management and analysis. Nature reviews genetics 2010;11(9):647.
5. Schadt EE, Linderman MD, Sorenson J, Lee L, Nolan GP. Cloud and heterogeneous computing solutions exist today for the emerging big data problems in biology. Nature Reviews Genetics 2011;12(3):224.
6. Lau JW, Lehnert E, Sethi A, Malhotra R, Kaushik G, Onder Z, et al. The Cancer Genomics Cloud: collaborative, reproducible, and democratized—a new paradigm in large-scale computational research. Cancer research 2017;77(21):e3–e6.
7. Reynolds SM, Miller M, Lee P, Leinonen K, Paquette SM, Rodebaugh Z, et al. The ISB Cancer Genomics Cloud: a flexible cloud-based platform for cancer genomics research. Cancer research 2017;77(21):e7–e10.
8. Afgan E, Baker D, Coraor N, Goto H, Paul IM, Makova KD, et al. Harnessing cloud computing with Galaxy Cloud. Nature biotechnology 2011;29(11):972.
9. Birger C, Hanna M, Salinas E, Neff J, Saksena G, Livitz D, et al. FireCloud, a scalable cloud-based platform for collaborative genome analysis: Strategies for reducing and controlling costs. bioRxiv 2017;p. 209494.
10. Tatlow P, Piccolo SR. A cloud-based workflow to quantify transcript-expression levels in public cancer compendia. Scientific reports 2016;6:39259.
11. Lachmann A, Torre D, Keenan AB, Jagodnik KM, Lee HJ, Wang L, et al. Massive mining of publicly available RNA-seq data from human and mouse. Nature communications 2018;9(1):1366.
12. Juve G, Chervenak A, Deelman E, Bharathi S, Mehta G, Vahi K. Characterizing and profiling scientific workflows. Future Generation Computer Systems 2013;29(3):682–692.
13. Tyryshkina A, Coraor N, Nekrutenko A. Predicting runtimes of bioinformatics tools based on historical data: five years of Galaxy usage. Bioinformatics 2019;35(18):3453–3460.
14. Weingärtner R, Bräscher GB, Westphall CB. Cloud resource management: A survey on forecasting and profiling models. Journal of Network and Computer Applications 2015;47:99–106.
15. Gregg B. Thinking methodically about performance. Communications of the ACM 2013;56(2):45–51.
16. Lloyd W, David O, Arabi M, Ascough I, JC G, TR C, et al. The virtual machine (VM) scaler: an infrastructure manager supporting environmental modeling on IaaS clouds. In: Environmental Modeling International Conference Proceedings; 2014. .
17. Lloyd WJ, Pallickara S, David O, Arabi M, Wible T, Ditty J, et al. Demystifying the clouds: Harnessing resource utilization models for cost effective infrastructure alternatives. IEEE Transactions on Cloud Computing 2017;5(4):667–680.
18. 12 Best Docker Container Monitoring Tools [2022 Comparison] - Sematext;. (Accessed 02/2023). https://sematext.com/blog/docker-container-monitoring/.
19. f18m/cmonitor: A Docker/LXC/Kubernetes, database-free, lightweight container performance monitoring solution, perfect for ephemeral containers (e.g. containers used for DevOps automatic testing);. (Accessed 02/2023). https://github.com/f18m/cmonitor.
20. Ji S, Ye K, Xu CZ. Cmonitor: A monitoring and alarming platform for container-based clouds. In: International Conference on Cloud Computing Springer; 2019. p. 324–339.
21. Mathá R, Kimovski D, Zabrovskiy A, Timmerer C, Prodan R. Where to Encode: A Performance Analysis of x86 and Arm-based Amazon EC2 Instances. In: 2021 IEEE 17th International Conference on eScience (eScience) IEEE; 2021. p. 118–127.
22. Lambion D, Schmitz R, Cordingly R, Heydari N, Lloyd W. Characterizing X86 and ARM Serverless Performance Variation: A Natural Language Processing Case Study. In: Companion of the 2022 ACM/SPEC International Conference on Performance Engineering ICPE '22, New York, NY, USA: Association for Computing Machinery; 2022. p. 69–75. https://doi.org/10.1145/3491204.3543506.
23. Jiang Q, Lee YC, Zomaya AY. The power of ARM64 in public clouds. In: 2020 20th IEEE/ACM International Symposium on Cluster, Cloud and Internet Computing (CCGRID) IEEE; 2020. p. 459–468.
24. Rodola G, psutil - PyPI;. (Accessed 02/2023). https://pypi.org/project/psutil/.
25. proc(5) - Linux manual page - process information pseudo-filesystem;. (Accessed 02/2023). http://man7.org/linux/man-pages/man5/proc.5.html.
26. Linux Howtos: System -> /proc/stat explained;. (Accessed 02/2023). http://www.linuxhowtos.org/System/procstat.htm.
27. KB941772: Gathering CPU Utilization from /proc/stat;. (Accessed 02/2023). https://www.idnt.net/en-US/kb/941772.
28. /proc/cpuinfo (E.2.3.), Red Hat Enterprise Linux 6, Red Hat Customer Portal;. (Accessed 04/13/2020). https://access.redhat.com/documentation/en-us/red_hat_enterprise_linux/6/html/deployment_guide/s2-proc-cpuinfo.
29. /procfs/diskstats;. (Accessed 02/2023). https://www.kernel.org/doc/Documentation/ABI/testing/procfs-diskstats.
30. /proc/meminfo (E.2.18.), Red Hat Enterprise Linux 6, Red Hat Customer Portal;. (Accessed 02/2023). https://access.redhat.com/documentation/en-us/red_hat_enterprise_linux/6/html/deployment_guide/s2-proc-meminfo.
31. /proc/net/ (E.3.7.), Red Hat Enterprise Linux 6, Red Hat Customer Portal;. (Accessed 02/2023). https://access.redhat.com/documentation/en-us/red_hat_enterprise_linux/6/html/deployment_guide/s2-proc-dir-net.
32. /proc/loadavg (E.2.15.), Red Hat Enterprise Linux 6, Red Hat Customer Portal;. (Accessed 02/2023). https://access.redhat.com/documentation/en-us/red_hat_enterprise_linux/6/html/deployment_guide/s2-proc-loadavg.
33. cgroup/cpuacct (3.3.), Red Hat Enterprise Linux 6, Red Hat Customer Portal;. (Accessed 02/2023). https://access.redhat.com/documentation/en-us/red_hat_enterprise_linux/6/html/resource_management_guide/sec-cpuacct.
34. Chapter 3. Subsystems and Tunable Parameters, Red Hat Enterprise Linux 6, Red Hat Customer Portal;. (Accessed 02/2023). https://access.redhat.com/documentation/en-us/red_hat_enterprise_linux/6/html/resource_management_guide/ch-subsystems_and_tunable_parameters.
35. /cgroup/memory (3.7.), Red Hat Enterprise Linux 6, Red Hat Customer Portal;. (Accessed 02/2023). https://access.redhat.com/documentation/en-us/red_hat_enterprise_linux/6/html/resource_management_guide/sec-memory.
36. /proc/net/ (E.3.7.), Red Hat Enterprise Linux 6, Red Hat

Customer Portal;. (Accessed 02/2023). https://access.redhat.com/documentation/en-us/red_hat_enterprise_linux/6/html/deployment_guide/s2-proc-dir-net.
37. Linux Programmer's Manual;. (Accessed 02/2023). http://man7.org/linux/man-pages/man7/cgroups.7.html.
38. Xiong Y, Soumillon M, Wu J, Hansen J, Hu B, van Hasselt JGC, et al. A Comparison of mRNA Sequencing with Random Primed and 3′-Directed Libraries. Scientific Reports 2017;7(1):14626. https://doi.org/10.1038/s41598-017-14892-x.
39. Soumillon M, Cacchiarelli D, Semrau S, van Oudenaarden A, Mikkelsen TS. Characterization of directed differentiation by high-throughput single-cell RNA-Seq. BioRxiv 2014;p. 003236.
40. Hung LH, Lloyd W, Agumbe Sridhar R, Athmalingam Ravishankar SD, Xiong Y, Sobie E, et al. Holistic optimization of an RNA-seq workflow for multi-threaded environments. Bioinformatics 2019;35(20):4173–4175.

 Click here to access/download;Personal Cover;Container-Profiler-Resubmission-GigaScience-CoverLetter.pdf

We are writing to submit a revised manuscript as a technical note for Gigascience. Our original submission (GIGA-D-20-00159) was titled “Profiling Resource Utilization of Bioinformatics Workflows”. The revised paper’s title is now “Container Profiler: Profiling Resource Utilization of Containerized Big Data Pipelines”.

Our article describes a new tool that helps in identifying performance bottlenecks to aid in optimizing the performance and costs of cloud computing for bioinformatics applications deployed using containers. This paper presents the ContainerProfiler, a software tool that measures and records the resource usage of any containerized task. Our tool profiles the CPU, memory, disk, and network utilization of any containerized task by collecting Linux operating system metrics at the virtual machine, container, and process levels. The Container Profiler can produce utilization snapshots at multiple time points, allowing for continuous monitoring of the resources consumed by a container workflow. For this resubmission, we have developed a new version of the tool that addresses performance issues and critiques from the initial manuscript submission.

To illustrate the utility of the Container Profiler, as a case study we examine the resource utilization of a multi-stage bioinformatics analytical pipeline for RNA sequencing data using unique molecular identifiers (UMI). We examine and visualize resource utilization metrics across four stages of this pipeline including download, split, align, and merge. We measured the profiling overhead introduced by the Container Profiler to investigate its impact on runtime with different profiling verbosities.

A pre-print of our manuscript (https://arxiv.org/pdf/2005.11491.pdf) has been made available on arXiv.

Below we describe how the revised manuscript addresses feedback from the initial submission. Responses to the reviewers appear in *italic text.*

Reviewer 1

* Please modify the figures in the paper (and in the tool, if applicable) to use color palette(s) that are distinguishable to people with red/green color blindness. The current figures are not colorblind friendly.

Figure 1, Figure 2, and Figure 3 have been recreated and recolored after checking with a color-blind image tool: https://www.color-blindness.com/coblis-color-blindness-simulator/, Other images only feature limited use of colors.

* In Figure 2, there is no reason for the graphs to be 3 dimensional. In fact, it makes it more difficult to see the sizes of the subsets and to compare the bars (see https://serialmentor.com/dataviz/no-3d.html). It is also difficult (for me) to see the colors in the legend of the graph, so it's difficult to interpret the meaning of the bars.

Figure 2 has been recreated and recolored and is no longer 3-dimensional. The size of the color boxes in the legend has been quadrupled. Colors are left-to-right in the legend, and top-to-bottom in the graph.

* It would be helpful to discuss the tradeoff between frequent polling and data-storage issues. Is all the profiling data stored in memory? For example, how much memory is required to store data that are collected at 1-second intervals for an hour-long process. At what point should users be concerned about memory usage (or the size of the resulting JSON files)?

We have included details in the Results section under the subheading: 'Container Profiler can sample container and host metrics with sub-second resolution'.

We profiled our UMI RNA-sequencing pipeline use case which required ~2.5 hours to execute. This pipeline featured up to 85-concurrent processes. Profiling with one-second sampling and full profiling verbosity (collection of all metrics: process-level + container-level + host-level), produced ~9,000 JSON files requiring 296 MB of storage space. We believe 296 MB is not a significant amount of storage space given the size of modern day storage systems.

* The GitHub repository should have a file that indicates the terms of the open-source license. As it is, the README file just says "Copyright." But the terms of reuse or liability are unclear. GitHub provides options for selecting a license.

The MIT License is now included.

* It would be very helpful if the README page on GitHub had a brief tutorial about how to install and use the tool. The paper provides some insight on this, but it would be helpful to provide more detailed and specific instructions there.

The GitHub description has been expanded to include instructions on how to install and use the tool. In addition a series of videos have been posted on a YouTube channel which is linked from the project's GitHub.

Container Profiler YouTube Channel:
https://www.youtube.com/@containerprofiler6371

* I was not able to test the app because I was unclear on how to do it. The paper mentions scripts called processpack.sh, runDockerProfile.sh, ru_profiler.sh, and rudataall.sh. But I do not see all of these in the GitHub repository. Although the functionality sounds very interesting in principle, I cannot be confident that this tool is useful to the research community until I can try it myself. It would be very useful to provide a full working example of using Container Profiler for one or more real-world data-processing tasks, such as the one described in the paper. (Also, there are some extra files in the GitHub repo that seem unnecessary…like an empty file called "1", etc.)

The tool has been redesigned for the paper resubmission. Figure 6 now describes the structure. The GitHub includes installation instructions as well as profiling examples to help users carry out a variety of profiling tasks depending on the profiling use case.

* The paper mentions the ability to produce visualizations, but there are few details about how to do this. I am also unsure about this because I am not sure what software is used to create the visualizations or how to install those (or whether there is some way of doing this within a Docker container). Please provide information on this so that I and others can do that.

The GitHub now includes instructions on how to generate graphs. In addition, a YouTube video has been created to demonstrate and describe how to generate graphs from the ContainerProfiler: ***https://youtu.be/cI8D4JRuyjw***

Reviewer 2

While resource monitoring is certainly an important technique, this paper does not present any new method. Essentially, the monitoring is already done in the Linux kernel, and the provided tool simply reads out those values and reports them without any further analysis. There is no discussion of any particular challenge or problem encountered in doing so.

While the Linux kernel provides profiling metrics, they are not organized, archived, or aggregated in a usable way. In particular, profiling metrics are not accessible to novice or casual users, those who may seek to profile bioinformatics pipelines. The paper discusses the requirement to have a fast tool which is capable of time series sampling at a one second interval. The tool must be able to profile all metrics in under one second while also not increasing the execution time of the pipeline being profiled.

The performance is quite slow (typically ~1s per sample) and the paper does not explore or explain why over 10% of the process-level measurements take ~9s. The reader may guess that part of the problem lies in invoking a new shell script that invokes a new python process to open and access a large number of files.

To address performance issues with the ContainerProfiler in the previous paper submission, we have rewritten the tool reducing samping time from 9,000 milliseconds for full verbosity sampling (process + container + vm metrics) to under 100 milliseconds. ***We provide a 90x performance improvement over the previous version.*** *The tool can profile any containerized pipeline while sustaining one-second periodicity of the sampling interval.*

Further, a quick google search for "container resource monitoring" shows a large number of commercial solutions in this space, which are not discussed. There are a number of open problems related to monitoring at high frequencies, monitoring short processes, monitoring policy violations, and the like, but this paper does not explore them.

While there are a number of commercial and open source tools, the majority of the available tools require the setup and maintenance of a complete monitoring application including a time-series database and web server. This effort can be considerable, creating a technical hurdle for users. Our intent is to offer a profiling solution which is lightweight with minimal-to-no setup

required. Biomedical scientists seeking to profile computational pipelines should not require extensive database or web server administration skills. These burdens will impact the likelihood that biomedical scientists will successfully profile their pipelines, preventing them from gaining a better understanding regarding how to optimize them for deployment to clouds and clusters.

The second half of the paper gives a suggestion as to what can be done with resource measurements, which is a more promising area to focus on research. A case study is given in which CPU utilization is much lower than expected for over two hours, and this is indicated as a problem. It may very well be, but any adjustment to the configuration of a program always involves a tradeoff between time, scale, and other resources consumed. This is a complex multi-constraint problem worthy of some study, but this paper does not address it.

Since the initial submission of the paper, errors in the merge step of the UMI RNA-sequencing use case have been corrected. The multi-hour period of CPU idle time is gone. The pipeline now only has a short idle period at the end of the alignment phase before completing the merge phase which is also shorter.

Reviewer 3

Container Profiler is implemented as a collection of bash and python scripts to collect performance data, including CPU, memory, disk, and network-related monitoring metrics. However, cache misses, branch predict, instructions per cycle, and other performance metrics of the hardware layer also play a key role in detecting the bottlenecks of the programs. Performance collection tool such as Perf [1] has included many metrics mentioned in the paper. It is recommended to refer to Perf for optimizing Container Profiler.

Process-level, container-level, and host-level memory page faults and major page faults are reported. Profiling cache misses, branch predictions, and instructions per cycle are primarily considered a low-level code profiling tasks which are performed at the source level and not at the container level. Providing simultaneous profiling of these metrics for all processes running concurrently inside a container is beyond the scope of the tool. A developer looking for this information can consider running the Perf tool to directly profile their code.

Container Profiler is a tool for online data collection and offline visual analysis. It is recommended to add visualization functions such as real-time display of CPU utilization and memory utilization. For details, please refer to the GPU performance tool Nvprof [2].

Adding real-time display features is not presently considered a goal or objective for the Container Profiler. Our focus has been to enable profiling total resource utilization deltas to observe the total resource footprint and also to perform time series sampling of computational pipelines. Real-time display of metrics is possible, but creation of a GUI and display add-on is beyond the scope of this paper.

The authors demonstrate the Container Profiler only using one workload. Is it validated on other workloads? Only one dataset may not be convincing enough.

Yes, we have validated the Container Profiler on multiple computational tasks (or workloads). Specifically, the ContainerProfiler is used to profile a four-stage unique molecular identifier (UMI) RNA sequencing analytical pipeline including distinct tasks: download, split, alignment, and merge. Each of these stages is implemented using a separate container and represents a distinct computational step in the analysis pipeline. Additionally, we have applied the Container Profiler to assess common Linux benchmarks including sysbench and pgbench. These benchmarks are presented as examples in the GitHub instructions and videos. In light of this comment, we defined the terms "pipelines" and "workflows" in the first paragraph of the paper.

The profiling tool saves the collected data in JSON files. If the workload runs for a long time and the sampling time interval is 1s, how to deal with the case of JSON text length overflow? It is recommended to save the data directly to the database or periodically clean the old data (or useless data) in JSON files.

The maximum supported size for a single JSON file is approximately 4 GB. On average, JSON files produced by the Container Profiler with full verbosity (host + container + process) are approximately 33 KB. JSON text overflow should not be an issue. The Container Profiler includes an option to purge existing files when repeatedly running an identical pipeline. For profiling our full UMI RNA-sequencing pipeline with full profiling verbosity (collection of all metrics: process-level + container-level + host-level) and one-second sampling, ~9,000 JSON files were collected requiring 296 MB of storage space. This pipeline featured up to 85 concurrent processes. For a 24-hour pipeline with a similar number of concurrent processes we estimate a data storage requirement of ~3GB with full verbosity. For long running pipelines, to reduce the storage requirements of profiling, the user can adjust the profiling interval from 1-second to 1-minute. This would reduce the storage requirement for a 24-hour job to approximately 50 MB.

The authors evaluate the overhead of the profiling tool, but they only verify the impact on the running time of the workload. Will it interfere with other metrics such as CPU or memory utilization?

We did not measure the % increase in CPU, disk, or network metrics. To determine baseline CPU, disk, and network utilization still requires a profiling tool. Perhaps it would be possible to create a tool that only measures one metric, and then repeat the profiling task many times to check the % increase in metrics from the baseline 1-metric-tool measurement vs. the Container Profiler. However, for an 8-hour dataset, the increase in runtime for the UMI RNA-Seq pipeline with full profiling verbosity and one-second sampling added just 3.4 minutes to the pipeline execution time, which is an increase of only 0.71%. We do not believe the effort to measure % increase in metric utilization from baseline will be particularly interesting. Utilization will increase, but likely not very much if runtime increases less than 1%. The goal of the ContainerProfiler here has been to profile containerized genomics computational pipelines.

Such pipelines typically consist of compute-bound and data I/O-bound steps which will feature much greater resource utilization than the profiling tasks performed by the ContainerProfiler.

Thank you for your consideration,

Wes J. Lloyd

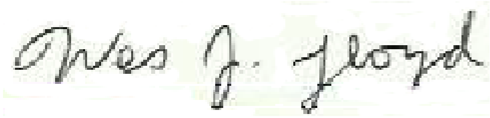

Associate Professor
Computer Science and Systems
School of Engineering and Technology
University of Washington Tacoma
