# Supplementary material for: Container Profiler: Profiling resource utilization of containerized big data pipelines
Source: Gigascience. 2023 Aug 25;12:giad069. doi: 10.1093/gigascience/giad069 (PMC10452954; doi:10.1093/gigascience/giad069)
Supplement: giad069_GIGA-D-23-00052_Revision_1 [file giad069_giga-d-23-00052_revision_1.pdf]

GigaScience

Container Profiler: Profiling Resource Utilization of Containerized Big Data Pipelines

--Manuscript Draft--

**Manuscript Number:**	GIGA-D-23-00052R1
**Full Title:**	Container Profiler: Profiling Resource Utilization of Containerized Big Data Pipelines
**Article Type:**	Technical Note
**Funding Information:**	National Institute of General Medical Sciences (R01GM126019) — Dr. Ka Yee Yeung<br>National Human Genome Research Institute (U24HG012674) — Dr. Ka Yee Yeung<br>Division of Microbiology and Infectious Diseases, National Institute of Allergy and Infectious Diseases (R03AI159286) — Dr. Ka Yee Yeung<br>Office of Advanced Cyberinfrastructure (1849970) — Dr. Wes Lloyd

Abstract:

Background This paper presents the Container Profiler, a software tool that measures and records the resource usage of any containerized task. Our tool profiles the CPU, memory, disk, and network utilization of containerized tasks collecting over fifty Linux operating system metrics at the virtual machine, container, and process levels. The Container Profiler supports performing time series profiling at a configurable sampling interval to enable continuous monitoring of the resources consumed by containerized tasks and pipelines.

Results To investigate the utility of the Container Profiler, we profile the resource utilization requirements of a multi-stage bioinformatics analytical pipeline (RNA sequencing using unique molecular identifiers). We examine profiling metrics to assess patterns of CPU, disk, and network resource utilization across the different stages of the pipeline. We also quantify the profiling overhead of our Container Profiler tool to assess the impact of profiling a running pipeline with different levels of profiling granularity verifying that impacts are negligible.

Conclusions The Container Profiler provides a useful tool that can be used to continuously monitor the resource consumption of long and complex containerized applications that run locally or on the cloud. This can help identify bottlenecks where more resources are needed to improve performance.

**Corresponding Author:**	Wes Lloyd<br>University of Washington, Tacoma<br>Tacoma, WA UNITED STATES
**Corresponding Author Secondary Information:**	
**Corresponding Author's Institution:**	University of Washington, Tacoma
**Corresponding Author's Secondary Institution:**	
**First Author:**	Varik Hoang
**First Author Secondary Information:**	
**Order of Authors:**	Varik Hoang<br>Ling-Hong Hung<br>David Perez<br>Huazeng Deng

Powered by Editorial Manager® and ProduXion Manager® from Aries Systems Corporation

	Raymond Schooley
	Niharika Arumilli
	Ka Yee Yeung
	Wes Lloyd
**Order of Authors Secondary Information:**	
**Response to Reviewers:**	All of the requested changes from Reviewer #1 have involved updates to the project's GitHub repository. These have been made.<br>The reviewers did not identify specific changes to the manuscript.<br>The authors have worked with the GigaScience editor to meet a number of other requirements including submitting data to GigaDB, providing funding information, ORCIDs, and registering the tool to obtain an RRID and bio.tools ID.<br>Please let us know of any further updates or modifications or additional information that is required.<br><br>Thank you !<br>Wes
**Additional Information:**	
**Question**	**Response**
Are you submitting this manuscript to a special series or article collection?	No
**Experimental design and statistics**<br><br>Full details of the experimental design and statistical methods used should be given in the Methods section, as detailed in our Minimum Standards Reporting Checklist. Information essential to interpreting the data presented should be made available in the figure legends.<br><br>Have you included all the information requested in your manuscript?	Yes
**Resources**<br><br>A description of all resources used, including antibodies, cell lines, animals and software tools, with enough information to allow them to be uniquely identified, should be included in the Methods section. Authors are strongly encouraged to cite Research Resource Identifiers (RRIDs) for antibodies, model organisms and tools, where possible.<br><br>Have you included the information	Yes

requested as detailed in our Minimum Standards Reporting Checklist?	
**Availability of data and materials**<br><br>All datasets and code on which the conclusions of the paper rely must be either included in your submission or deposited in publicly available repositories (where available and ethically appropriate), referencing such data using a unique identifier in the references and in the “Availability of Data and Materials” section of your manuscript.<br><br>Have you have met the above requirement as detailed in our Minimum Standards Reporting Checklist?	Yes

Powered by Editorial Manager® and ProduXion Manager® from Aries Systems Corporation

Click here to access/download;LaTeX - Main Document (TeX file);main.tex

```
This is pdfTeX, Version 3.141592653-2.6-1.40.24 (TeX Live 2022)
(preloaded format=pdflatex 2023.3.8)  2 AUG 2023 01:42
entering extended mode
 restricted \write18 enabled.
 %&-line parsing enabled.
**main.tex
(./main.tex
LaTeX2e <2022-11-01> patch level 1
L3 programming layer <2023-02-22> (./oup-contemporary.cls
Document Class: oup-contemporary 2017/06/28, v1.1
(c:/TeXLive/2022/texmf-dist/tex/latex/base/article.cls
Document Class: article 2022/07/02 v1.4n Standard LaTeX document class
(c:/TeXLive/2022/texmf-dist/tex/latex/base/size10.clo
File: size10.clo 2022/07/02 v1.4n Standard LaTeX file (size option)
)
\c@part=\count185
\c@section=\count186
\c@subsection=\count187
\c@subsubsection=\count188
\c@paragraph=\count189
\c@subparagraph=\count190
\c@figure=\count191
\c@table=\count192
\abovecaptionskip=\skip48
\belowcaptionskip=\skip49
\bibindent=\dimen140
) (c:/TeXLive/2022/texmf-dist/tex/latex/base/inputenc.sty
Package: inputenc 2021/02/14 v1.3d Input encoding file
\inpenc@prehook=\toks16
\inpenc@posthook=\toks17
) (c:/TeXLive/2022/texmf-dist/tex/latex/base/fontenc.sty
Package: fontenc 2021/04/29 v2.0v Standard LaTeX package
) (c:/TeXLive/2022/texmf-dist/tex/generic/iftex/ifpdf.sty
Package: ifpdf 2019/10/25 v3.4 ifpdf legacy package. Use iftex instead.
(c:/TeXLive/2022/texmf-dist/tex/generic/iftex/iftex.sty
Package: iftex 2022/02/03 v1.0f TeX engine tests
)) (c:/TeXLive/2022/texmf-dist/tex/latex/microtype/microtype.sty
Package: microtype 2023/03/13 v3.1a Micro-typographical refinements (RS)
(c:/TeXLive/2022/texmf-dist/tex/latex/graphics/keyval.sty
Package: keyval 2022/05/29 v1.15 key=value parser (DPC)
\KV@toks@=\toks18
) (c:/TeXLive/2022/texmf-dist/tex/latex/etoolbox/etoolbox.sty
Package: etoolbox 2020/10/05 v2.5k e-TeX tools for LaTeX (JAW)
\etb@tempcnta=\count193
)
\MT@toks=\toks19
\MT@tempbox=\box51
\MT@count=\count194
LaTeX Info: Redefining \noprotrusionifhmode on input line 1059.
LaTeX Info: Redefining \leftprotrusion on input line 1060.
\MT@prot@toks=\toks20
LaTeX Info: Redefining \rightprotrusion on input line 1078.
LaTeX Info: Redefining \textls on input line 1368.
\MT@outer@kern=\dimen141
```

```
LaTeX Info: Redefining \textmicrotypecontext on input line 1988.
\MT@listname@count=\count195
(c:/TeXLive/2022/texmf-dist/tex/latex/microtype/microtype-pdftex.def
File: microtype-pdftex.def 2023/03/13 v3.1a Definitions specific to
pdftex (RS)

LaTeX Info: Redefining \lsstyle on input line 902.
LaTeX Info: Redefining \lslig on input line 902.
\MT@outer@space=\skip50
)
Package microtype Info: Loading configuration file microtype.cfg.
(c:/TeXLive/2022/texmf-dist/tex/latex/microtype/microtype.cfg
File: microtype.cfg 2023/03/13 v3.1a microtype main configuration file
(RS)
)) (c:/TeXLive/2022/texmf-dist/tex/latex/euler/euler.sty
Package: euler 1995/03/05 v2.5
Package: `euler' v2.5 <1995/03/05> (FJ and FMi)
LaTeX Font Info:    Redeclaring symbol font `letters' on input line 35.
LaTeX Font Info:    Encoding `OML' has changed to `U' for symbol font
(Font)              `letters' in the math version `normal' on input line
35.
LaTeX Font Info:    Overwriting symbol font `letters' in version `normal'
(Font)                  OML/cmm/m/it --> U/eur/m/n on input line 35.
LaTeX Font Info:    Encoding `OML' has changed to `U' for symbol font
(Font)              `letters' in the math version `bold' on input line
35.
LaTeX Font Info:    Overwriting symbol font `letters' in version `bold'
(Font)                  OML/cmm/b/it --> U/eur/m/n on input line 35.
LaTeX Font Info:    Overwriting symbol font `letters' in version `bold'
(Font)                  U/eur/m/n --> U/eur/b/n on input line 36.
LaTeX Font Info:    Redeclaring math symbol \Gamma on input line 47.
LaTeX Font Info:    Redeclaring math symbol \Delta on input line 48.
LaTeX Font Info:    Redeclaring math symbol \Theta on input line 49.
LaTeX Font Info:    Redeclaring math symbol \Lambda on input line 50.
LaTeX Font Info:    Redeclaring math symbol \Xi on input line 51.
LaTeX Font Info:    Redeclaring math symbol \Pi on input line 52.
LaTeX Font Info:    Redeclaring math symbol \Sigma on input line 53.
LaTeX Font Info:    Redeclaring math symbol \Upsilon on input line 54.
LaTeX Font Info:    Redeclaring math symbol \Phi on input line 55.
LaTeX Font Info:    Redeclaring math symbol \Psi on input line 56.
LaTeX Font Info:    Redeclaring math symbol \Omega on input line 57.
\symEulerFraktur=\mathgroup4
LaTeX Font Info:    Overwriting symbol font `EulerFraktur' in version
`bold'
(Font)                  U/euf/m/n --> U/euf/b/n on input line 63.
LaTeX Info: Redefining \oldstylenums on input line 85.
\symEulerScript=\mathgroup5
LaTeX Font Info:    Overwriting symbol font `EulerScript' in version
`bold'
(Font)                  U/eus/m/n --> U/eus/b/n on input line 93.
LaTeX Font Info:    Redeclaring math symbol \aleph on input line 97.
LaTeX Font Info:    Redeclaring math symbol \Re on input line 98.
LaTeX Font Info:    Redeclaring math symbol \Im on input line 99.
LaTeX Font Info:    Redeclaring math delimiter \vert on input line 101.
```

```
LaTeX Font Info:    Redeclaring math delimiter \backslash on input line
103.
LaTeX Font Info:    Redeclaring math symbol \neg on input line 106.
LaTeX Font Info:    Redeclaring math symbol \wedge on input line 108.
LaTeX Font Info:    Redeclaring math symbol \vee on input line 110.
LaTeX Font Info:    Redeclaring math symbol \setminus on input line 112.
LaTeX Font Info:    Redeclaring math symbol \sim on input line 113.
LaTeX Font Info:    Redeclaring math symbol \mid on input line 114.
LaTeX Font Info:    Redeclaring math delimiter \arrowvert on input line
116.
LaTeX Font Info:    Redeclaring math symbol \mathsection on input line
117.
\symEulerExtension=\mathgroup6
LaTeX Font Info:    Redeclaring math symbol \coprod on input line 125.
LaTeX Font Info:    Redeclaring math symbol \prod on input line 125.
LaTeX Font Info:    Redeclaring math symbol \sum on input line 125.
LaTeX Font Info:    Redeclaring math symbol \intop on input line 130.
LaTeX Font Info:    Redeclaring math symbol \ointop on input line 131.
LaTeX Font Info:    Redeclaring math symbol \braceld on input line 132.
LaTeX Font Info:    Redeclaring math symbol \bracerd on input line 133.
LaTeX Font Info:    Redeclaring math symbol \bracelu on input line 134.
LaTeX Font Info:    Redeclaring math symbol \braceru on input line 135.
LaTeX Font Info:    Redeclaring math symbol \infty on input line 136.
LaTeX Font Info:    Redeclaring math symbol \nearrow on input line 153.
LaTeX Font Info:    Redeclaring math symbol \searrow on input line 154.
LaTeX Font Info:    Redeclaring math symbol \nwarrow on input line 155.
LaTeX Font Info:    Redeclaring math symbol \swarrow on input line 156.
LaTeX Font Info:    Redeclaring math symbol \Leftrightarrow on input line
157.
LaTeX Font Info:    Redeclaring math symbol \Leftarrow on input line 158.
LaTeX Font Info:    Redeclaring math symbol \Rightarrow on input line
159.
LaTeX Font Info:    Redeclaring math symbol \leftrightarrow on input line
160.
LaTeX Font Info:    Redeclaring math symbol \leftarrow on input line 161.
LaTeX Font Info:    Redeclaring math symbol \rightarrow on input line
163.
LaTeX Font Info:    Redeclaring math delimiter \uparrow on input line
166.
LaTeX Font Info:    Redeclaring math delimiter \downarrow on input line
168.
LaTeX Font Info:    Redeclaring math delimiter \updownarrow on input line
170.
LaTeX Font Info:    Redeclaring math delimiter \Uparrow on input line
172.
LaTeX Font Info:    Redeclaring math delimiter \Downarrow on input line
174.
LaTeX Font Info:    Redeclaring math delimiter \Updownarrow on input line
176.
LaTeX Font Info:    Redeclaring math symbol \leftharpoonup on input line
177.
LaTeX Font Info:    Redeclaring math symbol \leftharpoondown on input
line 178.
```

```
LaTeX Font Info:    Redeclaring math symbol \rightharpoonup on input line 
179.
LaTeX Font Info:    Redeclaring math symbol \rightharpoondown on input 
line 180
.
LaTeX Font Info:    Redeclaring math delimiter \lbrace on input line 182.
LaTeX Font Info:    Redeclaring math delimiter \rbrace on input line 184.
\symcmmigroup=\mathgroup7
LaTeX Font Info:    Overwriting symbol font `cmmigroup' in version `bold'
(Font)                  OML/cmm/m/it --> OML/cmm/b/it on input line 200.
LaTeX Font Info:    Redeclaring math accent \vec on input line 201.
LaTeX Font Info:    Redeclaring math symbol \triangleleft on input line 
202.
LaTeX Font Info:    Redeclaring math symbol \triangleright on input line 
203.
LaTeX Font Info:    Redeclaring math symbol \star on input line 204.
LaTeX Font Info:    Redeclaring math symbol \lhook on input line 205.
LaTeX Font Info:    Redeclaring math symbol \rhook on input line 206.
LaTeX Font Info:    Redeclaring math symbol \flat on input line 207.
LaTeX Font Info:    Redeclaring math symbol \natural on input line 208.
LaTeX Font Info:    Redeclaring math symbol \sharp on input line 209.
LaTeX Font Info:    Redeclaring math symbol \smile on input line 210.
LaTeX Font Info:    Redeclaring math symbol \frown on input line 211.
LaTeX Font Info:    Redeclaring math accent \grave on input line 245.
LaTeX Font Info:    Redeclaring math accent \acute on input line 246.
LaTeX Font Info:    Redeclaring math accent \tilde on input line 247.
LaTeX Font Info:    Redeclaring math accent \ddot on input line 248.
LaTeX Font Info:    Redeclaring math accent \check on input line 249.
LaTeX Font Info:    Redeclaring math accent \breve on input line 250.
LaTeX Font Info:    Redeclaring math accent \bar on input line 251.
LaTeX Font Info:    Redeclaring math accent \dot on input line 252.
LaTeX Font Info:    Redeclaring math accent \hat on input line 254.
) (c:/TeXLive/2022/texmf-dist/tex/latex/merriweather/merriweather.sty
Package: merriweather 2022/09/20 (Bob Tennent) Supports 
Merriweather(Sans) font
s for all LaTeX engines.
(c:/TeXLive/2022/texmf-dist/tex/generic/iftex/ifxetex.sty
Package: ifxetex 2019/10/25 v0.7 ifxetex legacy package. Use iftex 
instead.
) (c:/TeXLive/2022/texmf-dist/tex/generic/iftex/ifluatex.sty
Package: ifluatex 2019/10/25 v1.5 ifluatex legacy package. Use iftex 
instead.
) (c:/TeXLive/2022/texmf-dist/tex/latex/base/textcomp.sty
Package: textcomp 2020/02/02 v2.0n Standard LaTeX package
) (c:/TeXLive/2022/texmf-dist/tex/latex/xkeyval/xkeyval.sty
Package: xkeyval 2022/06/16 v2.9 package option processing (HA)
(c:/TeXLive/2022/texmf-dist/tex/generic/xkeyval/xkeyval.tex
(c:/TeXLive/2022/te
xmf-dist/tex/generic/xkeyval/xkvutils.tex
\XKV@toks=\toks21
\XKV@tempa@toks=\toks22
)
\XKV@depth=\count196
File: xkeyval.tex 2014/12/03 v2.7a key=value parser (HA)
```

```
)) (c:/TeXLive/2022/texmf-dist/tex/latex/base/fontenc.sty
Package: fontenc 2021/04/29 v2.0v Standard LaTeX package
) (c:/TeXLive/2022/texmf-dist/tex/latex/fontaxes/fontaxes.sty
Package: fontaxes 2020/07/21 v1.0e Font selection axes
LaTeX Info: Redefining \upshape on input line 29.
LaTeX Info: Redefining \itshape on input line 31.
LaTeX Info: Redefining \slshape on input line 33.
LaTeX Info: Redefining \swshape on input line 35.
LaTeX Info: Redefining \scshape on input line 37.
LaTeX Info: Redefining \sscshape on input line 39.
LaTeX Info: Redefining \ulcshape on input line 41.
LaTeX Info: Redefining \textsw on input line 47.
LaTeX Info: Redefining \textssc on input line 48.
LaTeX Info: Redefining \textulc on input line 49.
)) (c:/TeXLive/2022/texmf-dist/tex/latex/mathastext/mathastext.sty
Package: mathastext 2022/11/04 v1.3y Use the text font in math mode (JFB)
\mst@exists@muskip=\muskip16
\mst@forall@muskip=\muskip17
\mst@prime@muskip=\muskip18
\mst@do@nonletters=\toks23
\mst@do@easynonletters=\toks24
\mst@do@az=\toks25
\mst@do@AZ=\toks26
\symmtoperatorfont=\mathgroup8
\symmtletterfont=\mathgroup9
** ! and ?
** punctuation: , . : ; and \colon
LaTeX Info: Redefining \relbar on input line 844.
LaTeX Info: Redefining \rightarrowfill on input line 847.
LaTeX Info: Redefining \leftarrowfill on input line 852.
** + and =
LaTeX Info: Redefining \Relbar on input line 943.
** adding = ; and + to \nfss@catcodes
** parentheses ( ) [ ] and slash /
** alldelims: < > \backslash \setminus | \vert \mid \{ and \}
LaTeX Font Info:    Redeclaring math delimiter \backslash on input line
989.
LaTeX Font Info:    Redeclaring math symbol \setminus on input line 1001.
LaTeX Info: Redefining \models on input line 1010.
** \# \mathdollar \% \&
** \imath and \jmath
LaTeX Font Info:    Overwriting math alphabet `\mathnormalbold' in
version `nor
mal'
(Font)                  T1/Merriwthr-OsF/b/it --> T1/Merriwthr-OsF/b/it
on inpu
t line 2370.
LaTeX Font Info:    Overwriting math alphabet `\mathnormalbold' in
version `bol
d'
(Font)                  T1/Merriwthr-OsF/b/it --> T1/Merriwthr-OsF/b/it
on inpu
t line 2370.
```

```
LaTeX Font Info:    Overwriting symbol font `mtletterfont' in version
`normal'
(Font)                  T1/Merriwthr-OsF/m/it --> T1/Merriwthr-OsF/m/it
on inpu
t line 2370.
LaTeX Font Info:    Overwriting symbol font `mtletterfont' in version
`bold'
(Font)                  T1/Merriwthr-OsF/m/it --> T1/Merriwthr-OsF/b/it
on inpu
t line 2370.
LaTeX Font Info:    Overwriting symbol font `mtoperatorfont' in version
`normal
'
(Font)                  T1/Merriwthr-OsF/m/n --> T1/Merriwthr-OsF/m/n on
input
line 2370.
LaTeX Font Info:    Overwriting symbol font `mtoperatorfont' in version
`bold'
(Font)                  T1/Merriwthr-OsF/m/n --> T1/Merriwthr-OsF/b/n on
input
line 2370.
LaTeX Font Info:    Overwriting math alphabet `\Mathbf' in version
`normal'
(Font)                  T1/Merriwthr-OsF/b/n --> T1/Merriwthr-OsF/b/n on
input
line 2370.
LaTeX Font Info:    Overwriting math alphabet `\Mathbf' in version `bold'
(Font)                  T1/Merriwthr-OsF/b/n --> T1/Merriwthr-OsF/b/n on
input
line 2370.
LaTeX Font Info:    Overwriting math alphabet `\Mathit' in version
`normal'
(Font)                  T1/Merriwthr-OsF/m/it --> T1/Merriwthr-OsF/m/it
on inpu
t line 2370.
LaTeX Font Info:    Overwriting math alphabet `\Mathit' in version `bold'
(Font)                  T1/Merriwthr-OsF/m/it --> T1/Merriwthr-OsF/b/it
on inpu
t line 2370.
LaTeX Font Info:    Overwriting math alphabet `\Mathsf' in version
`normal'
(Font)                  T1/MerriwthrSans-OsF/m/n --> T1/MerriwthrSans-
OsF/m/n o
n input line 2370.
LaTeX Font Info:    Overwriting math alphabet `\Mathsf' in version `bold'
(Font)                  T1/MerriwthrSans-OsF/m/n --> T1/MerriwthrSans-
OsF/b/n o
n input line 2370.
LaTeX Font Info:    Overwriting math alphabet `\Mathtt' in version
`normal'
(Font)                  T1/lmtt/m/n --> T1/lmtt/m/n on input line 2370.
LaTeX Font Info:    Overwriting math alphabet `\Mathtt' in version `bold'
(Font)                  T1/lmtt/m/n --> T1/lmtt/b/n on input line 2370.
** Latin letters in the `normal' (resp. `bold') math versions are now
```

```
** set up to use the fonts T1/Merriwthr-OsF/m(b)/it
** Other characters (digits, ...) and \log-like names will be
** typeset with the n shape.
** \hbar
** minus as endash
** \HUGE has been (re)-defined.
** mathastext has declared larger sizes for subscripts.
** To keep LaTeX defaults, use option `defaultmathsizes'.
) (c:/TeXLive/2022/texmf-dist/tex/latex/relsize/relsize.sty
Package: relsize 2013/03/29 ver 4.1
) (c:/TeXLive/2022/texmf-dist/tex/latex/ragged2e/ragged2e.sty
Package: ragged2e 2023/02/25 v3.4 ragged2e Package
\CenteringLeftskip=\skip51
\RaggedLeftLeftskip=\skip52
\RaggedRightLeftskip=\skip53
\CenteringRightskip=\skip54
\RaggedLeftRightskip=\skip55
\RaggedRightRightskip=\skip56
\CenteringParfillskip=\skip57
\RaggedLeftParfillskip=\skip58
\RaggedRightParfillskip=\skip59
\JustifyingParfillskip=\skip60
\CenteringParindent=\skip61
\RaggedLeftParindent=\skip62
\RaggedRightParindent=\skip63
\JustifyingParindent=\skip64
) (c:/TeXLive/2022/texmf-dist/tex/latex/xcolor/xcolor.sty
Package: xcolor 2022/06/12 v2.14 LaTeX color extensions (UK)
(c:/TeXLive/2022/texmf-dist/tex/latex/graphics-cfg/color.cfg
File: color.cfg 2016/01/02 v1.6 sample color configuration
)
Package xcolor Info: Driver file: pdftex.def on input line 227.
(c:/TeXLive/2022/texmf-dist/tex/latex/graphics-def/pdftex.def
File: pdftex.def 2022/09/22 v1.2b Graphics/color driver for pdftex
) (c:/TeXLive/2022/texmf-dist/tex/latex/graphics/mathcolor.ltx)
Package xcolor Info: Model `cmy' substituted by `cmy0' on input line
1353.
Package xcolor Info: Model `hsb' substituted by `rgb' on input line 1357.
Package xcolor Info: Model `RGB' extended on input line 1369.
Package xcolor Info: Model `HTML' substituted by `rgb' on input line
1371.
Package xcolor Info: Model `Hsb' substituted by `hsb' on input line 1372.
Package xcolor Info: Model `tHsb' substituted by `hsb' on input line
1373.
Package xcolor Info: Model `HSB' substituted by `hsb' on input line 1374.
Package xcolor Info: Model `Gray' substituted by `gray' on input line
1375.
Package xcolor Info: Model `wave' substituted by `hsb' on input line
1376.
) (c:/TeXLive/2022/texmf-dist/tex/latex/colortbl/colortbl.sty
Package: colortbl 2022/06/20 v1.0f Color table columns (DPC)
(c:/TeXLive/2022/texmf-dist/tex/latex/tools/array.sty
Package: array 2022/09/04 v2.5g Tabular extension package (FMi)
\col@sep=\dimen142
```

```
\ar@mcellbox=\box52
\extrarowheight=\dimen143
\NC@list=\toks27
\extratabsurround=\skip65
\backup@length=\skip66
\ar@cellbox=\box53
)
\everycr=\toks28
\minrowclearance=\skip67
\rownum=\count197
) (c:/TeXLive/2022/texmf-dist/tex/latex/graphics/graphicx.sty
Package: graphicx 2021/09/16 v1.2d Enhanced LaTeX Graphics (DPC,SPQR)
(c:/TeXLive/2022/texmf-dist/tex/latex/graphics/graphics.sty
Package: graphics 2022/03/10 v1.4e Standard LaTeX Graphics (DPC,SPQR)
(c:/TeXLive/2022/texmf-dist/tex/latex/graphics/trig.sty
Package: trig 2021/08/11 v1.11 sin cos tan (DPC)
) (c:/TeXLive/2022/texmf-dist/tex/latex/graphics-cfg/graphics.cfg
File: graphics.cfg 2016/06/04 v1.11 sample graphics configuration
)
Package graphics Info: Driver file: pdftex.def on input line 107.
)
\Gin@req@height=\dimen144
\Gin@req@width=\dimen145
) (c:/TeXLive/2022/texmf-dist/tex/latex/xpatch/xpatch.sty
(c:/TeXLive/2022/texm
f-dist/tex/latex/l3kernel/expl3.sty
Package: expl3 2023-02-22 L3 programming layer (loader)
(c:/TeXLive/2022/texmf-dist/tex/latex/l3backend/l3backend-pdftex.def
File: l3backend-pdftex.def 2023-01-16 L3 backend support: PDF output
(pdfTeX)
\l__color_backend_stack_int=\count198
\l__pdf_internal_box=\box54
))
Package: xpatch 2020/03/25 v0.3a Extending etoolbox patching commands
(c:/TeXLive/2022/texmf-dist/tex/latex/l3packages/xparse/xparse.sty
Package: xparse 2023-02-02 L3 Experimental document command parser
)) (c:/TeXLive/2022/texmf-dist/tex/latex/environ/environ.sty
Package: environ 2014/05/04 v0.3 A new way to define environments
(c:/TeXLive/2022/texmf-dist/tex/latex/trimspaces/trimspaces.sty
Package: trimspaces 2009/09/17 v1.1 Trim spaces around a token list
)
\@envbody=\toks29
) (c:/TeXLive/2022/texmf-dist/tex/latex/lastpage/lastpage.sty
Package: lastpage 2023/03/07 v2.0a lastpage: 2.09 or 2e? (HMM)
(c:/TeXLive/2022/texmf-dist/tex/latex/lastpage/lastpage2e.sty
Package: lastpage2e 2023/03/07 v2.0a Decide which 2e lastpage version to
use (H
MM)
(c:/TeXLive/2022/texmf-dist/tex/latex/lastpage/lastpagemodern.sty
Package: lastpagemodern 2023-03-07 v2.0a Refers to last page's name (HMM;
JPG)
)
)) (c:/TeXLive/2022/texmf-dist/tex/latex/graphics/rotating.sty
Package: rotating 2016/08/11 v2.16d rotated objects in LaTeX
```

```
(c:/TeXLive/2022/texmf-dist/tex/latex/base/ifthen.sty
Package: ifthen 2022/04/13 v1.1d Standard LaTeX ifthen package (DPC)
)
\c@r@tfl@t=\count199
\rotFPtop=\skip68
\rotFPbot=\skip69
\rot@float@box=\box55
\rot@mess@toks=\toks30
) (c:/TeXLive/2022/texmf-dist/tex/latex/graphics/lscape.sty
Package: lscape 2020/05/28 v3.02 Landscape Pages (DPC)
) (c:/TeXLive/2022/texmf-dist/tex/latex/tools/afterpage.sty
Package: afterpage 2014/10/28 v1.08 After-Page Package (DPC)
\AP@output=\toks31
\AP@partial=\box56
\AP@footins=\box57
) (c:/TeXLive/2022/texmf-dist/tex/latex/textpos/textpos.sty
Package: textpos 2022/07/23 v1.10.1
Package textpos Info: choosing support for LaTeX3 on input line 60.
\TP@textbox=\box58
\TP@holdbox=\box59
\TPHorizModule=\dimen146
\TPVertModule=\dimen147
\TP@margin=\dimen148
\TP@absmargin=\dimen149
Grid set 16 x 16 = 37.34424pt x 52.81541pt
\TPboxrulesize=\dimen150
\TP@ox=\dimen151
\TP@oy=\dimen152
\TP@tbargs=\toks32
TextBlockOrigin set to 0pt x 0pt
) (c:/TeXLive/2022/texmf-dist/tex/latex/url/url.sty
\Urlmuskip=\muskip19
Package: url 2013/09/16  ver 3.4  Verb mode for urls, etc.
) (c:/TeXLive/2022/texmf-dist/tex/latex/newfloat/newfloat.sty
Package: newfloat 2019/09/02 v1.1l Defining new floating environments
(AR)
Package newfloat Info: `rotating' package detected.
) (c:/TeXLive/2022/texmf-dist/tex/latex/mdframed/mdframed.sty
Package: mdframed 2013/07/01 1.9b: mdframed
(c:/TeXLive/2022/texmf-dist/tex/latex/kvoptions/kvoptions.sty
Package: kvoptions 2022-06-15 v3.15 Key value format for package options
(HO)
(c:/TeXLive/2022/texmf-dist/tex/generic/ltxcmds/ltxcmds.sty
Package: ltxcmds 2020-05-10 v1.25 LaTeX kernel commands for general use
(HO)
) (c:/TeXLive/2022/texmf-dist/tex/latex/kvsetkeys/kvsetkeys.sty
Package: kvsetkeys 2022-10-05 v1.19 Key value parser (HO)
)) (c:/TeXLive/2022/texmf-dist/tex/latex/zref/zref-abspage.sty
Package: zref-abspage 2022-04-07 v2.34 Module abspage for zref (HO)
(c:/TeXLive/2022/texmf-dist/tex/latex/zref/zref-base.sty
Package: zref-base 2022-04-07 v2.34 Module base for zref (HO)
(c:/TeXLive/2022/texmf-dist/tex/generic/infwarerr/infwarerr.sty
Package: infwarerr 2019/12/03 v1.5 Providing info/warning/error messages
(HO)
```

```
) (c:/TeXLive/2022/texmf-dist/tex/generic/kvdefinekeys/kvdefinekeys.sty
Package: kvdefinekeys 2019-12-19 v1.6 Define keys (HO)
) (c:/TeXLive/2022/texmf-dist/tex/generic/pdftexcmds/pdftexcmds.sty
Package: pdftexcmds 2020-06-27 v0.33 Utility functions of pdfTeX for
LuaTeX (HO
)
Package pdftexcmds Info: \pdf@primitive is available.
Package pdftexcmds Info: \pdf@ifprimitive is available.
Package pdftexcmds Info: \pdfdraftmode found.
) (c:/TeXLive/2022/texmf-dist/tex/generic/etexcmds/etexcmds.sty
Package: etexcmds 2019/12/15 v1.7 Avoid name clashes with e-TeX commands
(HO)
) (c:/TeXLive/2022/texmf-dist/tex/latex/auxhook/auxhook.sty
Package: auxhook 2019-12-17 v1.6 Hooks for auxiliary files (HO)
)
Package zref Info: New property list: main on input line 767.
Package zref Info: New property: default on input line 768.
Package zref Info: New property: page on input line 769.
) (c:/TeXLive/2022/texmf-dist/tex/latex/base/atbegshi-ltx.sty
Package: atbegshi-ltx 2021/01/10 v1.0c Emulation of the original atbegshi
package with kernel methods
)
\c@abspage=\count266
Package zref Info: New property: abspage on input line 65.
) (c:/TeXLive/2022/texmf-dist/tex/latex/needspace/needspace.sty
Package: needspace 2010/09/12 v1.3d reserve vertical space
)
\mdf@templength=\skip70
\c@mdf@globalstyle@cnt=\count267
\mdf@skipabove@length=\skip71
\mdf@skipbelow@length=\skip72
\mdf@leftmargin@length=\skip73
\mdf@rightmargin@length=\skip74
\mdf@innerleftmargin@length=\skip75
\mdf@innerrightmargin@length=\skip76
\mdf@innertopmargin@length=\skip77
\mdf@innerbottommargin@length=\skip78
\mdf@splittopskip@length=\skip79
\mdf@splitbottomskip@length=\skip80
\mdf@outermargin@length=\skip81
\mdf@innermargin@length=\skip82
\mdf@linewidth@length=\skip83
\mdf@innerlinewidth@length=\skip84
\mdf@middlelinewidth@length=\skip85
\mdf@outerlinewidth@length=\skip86
\mdf@roundcorner@length=\skip87
\mdf@footenotedistance@length=\skip88
\mdf@userdefinedwidth@length=\skip89
\mdf@needspace@length=\skip90
\mdf@frametitleaboveskip@length=\skip91
\mdf@frametitlebelowskip@length=\skip92
\mdf@frametitlerulewidth@length=\skip93
\mdf@frametitleleftmargin@length=\skip94
\mdf@frametitlerightmargin@length=\skip95
```

```
\mdf@shadowsize@length=\skip96
\mdf@extratopheight@length=\skip97
\mdf@subtitleabovelinewidth@length=\skip98
\mdf@subtitlebelowlinewidth@length=\skip99
\mdf@subtitleaboveskip@length=\skip100
\mdf@subtitlebelowskip@length=\skip101
\mdf@subtitleinneraboveskip@length=\skip102
\mdf@subtitleinnerbelowskip@length=\skip103
\mdf@subsubtitleabovelinewidth@length=\skip104
\mdf@subsubtitlebelowlinewidth@length=\skip105
\mdf@subsubtitleaboveskip@length=\skip106
\mdf@subsubtitlebelowskip@length=\skip107
\mdf@subsubtitleinneraboveskip@length=\skip108
\mdf@subsubtitleinnerbelowskip@length=\skip109
(c:/TeXLive/2022/texmf-dist/tex/latex/mdframed/md-frame-0.mdf
File: md-frame-0.mdf 2013/07/01\ 1.9b: md-frame-0
)
\mdf@frametitlebox=\box60
\mdf@footnotebox=\box61
\mdf@splitbox@one=\box62
\mdf@splitbox@two=\box63
\mdf@splitbox@save=\box64
\mdfsplitboxwidth=\skip110
\mdfsplitboxtotalwidth=\skip111
\mdfsplitboxheight=\skip112
\mdfsplitboxdepth=\skip113
\mdfsplitboxtotalheight=\skip114
\mdfframetitleboxwidth=\skip115
\mdfframetitleboxtotalwidth=\skip116
\mdfframetitleboxheight=\skip117
\mdfframetitleboxdepth=\skip118
\mdfframetitleboxtotalheight=\skip119
\mdffootnoteboxwidth=\skip120
\mdffootnoteboxtotalwidth=\skip121
\mdffootnoteboxheight=\skip122
\mdffootnoteboxdepth=\skip123
\mdffootnoteboxtotalheight=\skip124
\mdftotallinewidth=\skip125
\mdfboundingboxwidth=\skip126
\mdfboundingboxtotalwidth=\skip127
\mdfboundingboxheight=\skip128
\mdfboundingboxdepth=\skip129
\mdfboundingboxtotalheight=\skip130
\mdf@freevspace@length=\skip131
\mdf@horizontalwidthofbox@length=\skip132
\mdf@verticalmarginwhole@length=\skip133
\mdf@horizontalspaceofbox=\skip134
\mdfsubtitleheight=\skip135
\mdfsubsubtitleheight=\skip136
\c@mdfcountframes=\count268

****** mdframed patching \endmdf@trivlist

****** -- success******
```

```
\mdf@envdepth=\count269
\c@mdf@env@i=\count270
\c@mdf@env@ii=\count271
\c@mdf@zref@counter=\count272
Package zref Info: New property: mdf@pagevalue on input line 895.
) (c:/TeXLive/2022/texmf-dist/tex/latex/titlesec/titlesec.sty
Package: titlesec 2021/07/05 v2.14 Sectioning titles
\ttl@box=\box65
\beforetitleunit=\skip137
\aftertitleunit=\skip138
\ttl@plus=\dimen153
\ttl@minus=\dimen154
\ttl@toksa=\toks33
\titlewidth=\dimen155
\titlewidthlast=\dimen156
\titlewidthfirst=\dimen157
) (c:/TeXLive/2022/texmf-dist/tex/latex/koma-script/scrextend.sty
Package: scrextend 2022/10/12 v3.38 KOMA-Script package (extend other
classes w
ith features of KOMA-Script classes)
(c:/TeXLive/2022/texmf-dist/tex/latex/koma-script/scrkbase.sty
Package: scrkbase 2022/10/12 v3.38 KOMA-Script package (KOMA-Script-
dependent b
asics and keyval usage)
(c:/TeXLive/2022/texmf-dist/tex/latex/koma-script/scrbase.sty
Package: scrbase 2022/10/12 v3.38 KOMA-Script package (KOMA-Script-
independent
basics and keyval usage)
(c:/TeXLive/2022/texmf-dist/tex/latex/koma-script/scrlfile.sty
Package: scrlfile 2022/10/12 v3.38 KOMA-Script package (file load hooks)
(c:/TeXLive/2022/texmf-dist/tex/latex/koma-script/scrlfile-hook.sty
Package: scrlfile-hook 2022/10/12 v3.38 KOMA-Script package (using LaTeX
hooks)

(c:/TeXLive/2022/texmf-dist/tex/latex/koma-script/scrlogo.sty
Package: scrlogo 2022/10/12 v3.38 KOMA-Script package (logo)
)))
Applying: [2021/05/01] Usage of raw or classic option list on input line
252.
Already applied: [0000/00/00] Usage of raw or classic option list on
input line
 368.
))
Package scrextend Info: unexpected definition of `\@makefnmark'.
(scrextend)             Trying to patch it on input line 1709.
Package scrextend Info: patch seems to be successfull on input line 1709.
)

LaTeX Font Warning: Font shape `T1/cmr/m/n' in size <7.5> not available
(Font)              size <7> substituted on input line 65.

(c:/TeXLive/2022/texmf-dist/tex/latex/tools/calc.sty
Package: calc 2017/05/25 v4.3 Infix arithmetic (KKT,FJ)
```

```
\calc@Acount=\count273
\calc@Bcount=\count274
\calc@Adimen=\dimen158
\calc@Bdimen=\dimen159
\calc@Askip=\skip139
\calc@Bskip=\skip140
LaTeX Info: Redefining \setlength on input line 80.
LaTeX Info: Redefining \addtolength on input line 81.
\calc@Ccount=\count275
\calc@Cskip=\skip141
) (c:/TeXLive/2022/texmf-dist/tex/latex/geometry/geometry.sty
Package: geometry 2020/01/02 v5.9 Page Geometry
(c:/TeXLive/2022/texmf-dist/tex/generic/iftex/ifvtex.sty
Package: ifvtex 2019/10/25 v1.7 ifvtex legacy package. Use iftex instead.
)
\Gm@cnth=\count276
\Gm@cntv=\count277
\c@Gm@tempcnt=\count278
\Gm@bindingoffset=\dimen160
\Gm@wd@mp=\dimen161
\Gm@odd@mp=\dimen162
\Gm@even@mp=\dimen163
\Gm@layoutwidth=\dimen164
\Gm@layoutheight=\dimen165
\Gm@layouthoffset=\dimen166
\Gm@layoutvoffset=\dimen167
\Gm@dimlist=\toks34
) (c:/TeXLive/2022/texmf-dist/tex/latex/hyperref/hyperref.sty
Package: hyperref 2023-02-07 v7.00v Hypertext links for LaTeX
(c:/TeXLive/2022/texmf-dist/tex/generic/pdfescape/pdfescape.sty
Package: pdfescape 2019/12/09 v1.15 Implements pdfTeX's escape features
(HO)
) (c:/TeXLive/2022/texmf-dist/tex/latex/hycolor/hycolor.sty
Package: hycolor 2020-01-27 v1.10 Color options for hyperref/bookmark
(HO)
) (c:/TeXLive/2022/texmf-dist/tex/latex/letltxmacro/letltxmacro.sty
Package: letltxmacro 2019/12/03 v1.6 Let assignment for LaTeX macros (HO)
) (c:/TeXLive/2022/texmf-dist/tex/latex/hyperref/nameref.sty
Package: nameref 2022-05-17 v2.50 Cross-referencing by name of section
(c:/TeXLive/2022/texmf-dist/tex/latex/refcount/refcount.sty
Package: refcount 2019/12/15 v3.6 Data extraction from label references
(HO)
) (c:/TeXLive/2022/texmf-
dist/tex/generic/gettitlestring/gettitlestring.sty
Package: gettitlestring 2019/12/15 v1.6 Cleanup title references (HO)
)
\c@section@level=\count279
)
\@linkdim=\dimen168
\Hy@linkcounter=\count280
\Hy@pagecounter=\count281
(c:/TeXLive/2022/texmf-dist/tex/latex/hyperref/pd1enc.def
File: pd1enc.def 2023-02-07 v7.00v Hyperref: PDFDocEncoding definition
(HO)
```

```
Now handling font encoding PD1 ...
... no UTF-8 mapping file for font encoding PD1
) (c:/TeXLive/2022/texmf-dist/tex/generic/intcalc/intcalc.sty
Package: intcalc 2019/12/15 v1.3 Expandable calculations with integers
(HO)
)
\Hy@SavedSpaceFactor=\count282
(c:/TeXLive/2022/texmf-dist/tex/latex/hyperref/puenc.def
File: puenc.def 2023-02-07 v7.00v Hyperref: PDF Unicode definition (HO)
Now handling font encoding PU ...
... no UTF-8 mapping file for font encoding PU
)
Package hyperref Info: Option `colorlinks' set `true' on input line 4060.
Package hyperref Info: Hyper figures OFF on input line 4177.
Package hyperref Info: Link nesting OFF on input line 4182.
Package hyperref Info: Hyper index ON on input line 4185.
Package hyperref Info: Plain pages OFF on input line 4192.
Package hyperref Info: Backreferencing OFF on input line 4197.
Package hyperref Info: Implicit mode ON; LaTeX internals redefined.
Package hyperref Info: Bookmarks ON on input line 4425.
\c@Hy@tempcnt=\count283
LaTeX Info: Redefining \url on input line 4763.
\XeTeXLinkMargin=\dimen169
(c:/TeXLive/2022/texmf-dist/tex/generic/bitset/bitset.sty
Package: bitset 2019/12/09 v1.3 Handle bit-vector datatype (HO)
(c:/TeXLive/2022/texmf-dist/tex/generic/bigintcalc/bigintcalc.sty
Package: bigintcalc 2019/12/15 v1.5 Expandable calculations on big
integers (HO
)
))
\Fld@menulength=\count284
\Field@Width=\dimen170
\Fld@charsize=\dimen171
Package hyperref Info: Hyper figures OFF on input line 6042.
Package hyperref Info: Link nesting OFF on input line 6047.
Package hyperref Info: Hyper index ON on input line 6050.
Package hyperref Info: backreferencing OFF on input line 6057.
Package hyperref Info: Link coloring ON on input line 6060.
Package hyperref Info: Link coloring with OCG OFF on input line 6067.
Package hyperref Info: PDF/A mode OFF on input line 6072.
\Hy@abspage=\count285
\c@Item=\count286
\c@Hfootnote=\count287
)
Package hyperref Info: Driver (autodetected): hpdftex.
(c:/TeXLive/2022/texmf-dist/tex/latex/hyperref/hpdftex.def
File: hpdftex.def 2023-02-07 v7.00v Hyperref driver for pdfTeX
(c:/TeXLive/2022/texmf-dist/tex/latex/base/atveryend-ltx.sty
Package: atveryend-ltx 2020/08/19 v1.0a Emulation of the original
atveryend pac
kage
with kernel methods
)
\HyAnn@Count=\count288
```

```
\Fld@listcount=\count289
\c@bookmark@seq@number=\count290
(c:/TeXLive/2022/texmf-dist/tex/latex/rerunfilecheck/rerunfilecheck.sty
Package: rerunfilecheck 2022-07-10 v1.10 Rerun checks for auxiliary files
(HO)
(c:/TeXLive/2022/texmf-dist/tex/generic/uniquecounter/uniquecounter.sty
Package: uniquecounter 2019/12/15 v1.4 Provide unlimited unique counter
(HO)
)
Package uniquecounter Info: New unique counter `rerunfilecheck' on input
line 2
85.
)
\Hy@SectionHShift=\skip142
) (c:/TeXLive/2022/texmf-dist/tex/latex/preprint/authblk.sty
Package: authblk 2001/02/27 1.3 (PWD)
\affilsep=\skip143
\@affilsep=\skip144
\c@Maxaffil=\count291
\c@authors=\count292
\c@affil=\count293
) (c:/TeXLive/2022/texmf-dist/tex/latex/footmisc/footmisc.sty
Package: footmisc 2022/03/08 v6.0d a miscellany of footnote facilities
\FN@temptoken=\toks35
\footnotemargin=\dimen172
\@outputbox@depth=\dimen173
Package footmisc Info: Declaring symbol style bringhurst on input line
695.
Package footmisc Info: Declaring symbol style chicago on input line 703.
Package footmisc Info: Declaring symbol style wiley on input line 712.
Package footmisc Info: Declaring symbol style lamport-robust on input
line 723.

Package footmisc Info: Declaring symbol style lamport* on input line 743.
Package footmisc Info: Declaring symbol style lamport*-robust on input
line 764
.
) (c:/TeXLive/2022/texmf-dist/tex/latex/fancyhdr/fancyhdr.sty
Package: fancyhdr 2022/11/09 v4.1 Extensive control of page headers and
footers

\f@nch@headwidth=\skip145
\f@nch@O@elh=\skip146
\f@nch@O@erh=\skip147
\f@nch@O@olh=\skip148
\f@nch@O@orh=\skip149
\f@nch@O@elf=\skip150
\f@nch@O@erf=\skip151
\f@nch@O@olf=\skip152
\f@nch@O@orf=\skip153
) (c:/TeXLive/2022/texmf-dist/tex/generic/alphalph/alphalph.sty
Package: alphalph 2019/12/09 v2.6 Convert numbers to letters (HO)
)
\c@authorfn=\count294
```

```
(c:/TeXLive/2022/texmf-dist/tex/latex/abstract/abstract.sty
Package: abstract 2009/06/08 v1.2a configurable abstracts
\abstitleskip=\skip154
\absleftindent=\skip155
\absrightindent=\skip156
\absparindent=\skip157
\absparsep=\skip158
)
Package newfloat Info: New float `keypoints' with options
`placement=t!,name=kp
t' on input line 286.
\c@keypoints=\count295
\newfloat@ftype=\count296
Package newfloat Info: float type `keypoints'=8 on input line 286.
(c:/TeXLive/2022/texmf-dist/tex/latex/enumitem/enumitem.sty
Package: enumitem 2019/06/20 v3.9 Customized lists
\labelindent=\skip159
\enit@outerparindent=\dimen174
\enit@toks=\toks36
\enit@inbox=\box66
\enit@count@id=\count297
\enitdp@description=\count298
) (c:/TeXLive/2022/texmf-dist/tex/latex/quoting/quoting.sty
Package: quoting 2014/01/28 v0.1c Consolidated environment for displayed
text
\quo@toppartop=\skip160
) (c:/TeXLive/2022/texmf-dist/tex/latex/sttools/stfloats.sty
Package: stfloats 2017/03/27 v3.3 Improve float mechanism and
baselineskip sett
ings
\@dblbotnum=\count299
\c@dblbotnumber=\count300
) (c:/TeXLive/2022/texmf-dist/tex/latex/booktabs/booktabs.sty
Package: booktabs 2020/01/12 v1.61803398 Publication quality tables
\heavyrulewidth=\dimen175
\lightrulewidth=\dimen176
\cmidrulewidth=\dimen177
\belowrulesep=\dimen178
\belowbottomsep=\dimen179
\aboverulesep=\dimen180
\abovetopsep=\dimen181
\cmidrulesep=\dimen182
\cmidrulekern=\dimen183
\defaultaddspace=\dimen184
\@cmidla=\count301
\@cmidlb=\count302
\@aboverulesep=\dimen185
\@belowrulesep=\dimen186
\@thisruleclass=\count303
\@lastruleclass=\count304
\@thisrulewidth=\dimen187
) (c:/TeXLive/2022/texmf-dist/tex/latex/tools/tabularx.sty
Package: tabularx 2020/01/15 v2.11c `tabularx' package (DPC)
\TX@col@width=\dimen188
```

```
\TX@old@table=\dimen189
\TX@old@col=\dimen190
\TX@target=\dimen191
\TX@delta=\dimen192
\TX@cols=\count305
\TX@ftn=\toks37
)
\enitdp@tablenotes=\count306
(c:/TeXLive/2022/texmf-dist/tex/latex/caption/caption.sty
Package: caption 2022/03/01 v3.6b Customizing captions (AR)
(c:/TeXLive/2022/texmf-dist/tex/latex/caption/caption3.sty
Package: caption3 2022/03/17 v2.3b caption3 kernel (AR)
\caption@tempdima=\dimen193
\captionmargin=\dimen194
\caption@leftmargin=\dimen195
\caption@rightmargin=\dimen196
\caption@width=\dimen197
\caption@indent=\dimen198
\caption@parindent=\dimen199
\caption@hangindent=\dimen256
Package caption Info: Standard document class detected.
)
\c@caption@flags=\count307
\c@continuedfloat=\count308
Package caption Info: hyperref package is loaded.
Package caption Info: rotating package is loaded.
) (c:/TeXLive/2022/texmf-dist/tex/latex/natbib/natbib.sty
Package: natbib 2010/09/13 8.31b (PWD, AO)
\bibhang=\skip161
\bibsep=\skip162
LaTeX Info: Redefining \cite on input line 694.
\c@NAT@ctr=\count309
)) (c:/TeXLive/2022/texmf-dist/tex/latex/siunitx/siunitx.sty
Package: siunitx 2023-03-04 v3.2.2 A comprehensive (SI) units package
\l__siunitx_angle_tmp_dim=\dimen257
\l__siunitx_angle_marker_box=\box67
\l__siunitx_angle_unit_box=\box68
\l__siunitx_compound_count_int=\count310
(c:/TeXLive/2022/texmf-dist/tex/latex/translations/translations.sty
Package: translations 2022/02/05 v1.12 internationalization of LaTeX2e
packages
 (CN)
)
\l__siunitx_number_exponent_fixed_int=\count311
\l__siunitx_number_min_decimal_int=\count312
\l__siunitx_number_min_integer_int=\count313
\l__siunitx_number_round_precision_int=\count314
\l__siunitx_number_lower_threshold_int=\count315
\l__siunitx_number_upper_threshold_int=\count316
\l__siunitx_number_group_first_int=\count317
\l__siunitx_number_group_size_int=\count318
\l__siunitx_number_group_minimum_int=\count319
(c:/TeXLive/2022/texmf-dist/tex/latex/amsmath/amstext.sty
Package: amstext 2021/08/26 v2.01 AMS text
```

```
(c:/TeXLive/2022/texmf-dist/tex/latex/amsmath/amsgen.sty
File: amsgen.sty 1999/11/30 v2.0 generic functions
\@emptytoks=\toks38
\ex@=\dimen258
))
\l__siunitx_table_tmp_box=\box69
\l__siunitx_table_tmp_dim=\dimen259
\l__siunitx_table_column_width_dim=\dimen260
\l__siunitx_table_integer_box=\box70
\l__siunitx_table_decimal_box=\box71
\l__siunitx_table_uncert_box=\box72
\l__siunitx_table_before_box=\box73
\l__siunitx_table_after_box=\box74
\l__siunitx_table_before_dim=\dimen261
\l__siunitx_table_carry_dim=\dimen262
\l__siunitx_unit_tmp_int=\count320
\l__siunitx_unit_position_int=\count321
\l__siunitx_unit_total_int=\count322
) (c:/TeXLive/2022/texmf-dist/tex/latex/floatrow/floatrow.sty
Package: floatrow 2008/08/02 v0.3b floatrow: float package extension
\c@float@type=\count323
\float@exts=\toks39
\float@box=\box75
\@floatcapt=\box76
Package floatrow Info: Modified float package code loaded on input line
455.
Package floatrow Info: Modified rotfloat package code loaded on input
line 473.

\FR@everyfloat=\toks40
\flrow@foot=\insert252
\FB@wd=\dimen263
\FBo@wd=\dimen264
\FBc@wd=\dimen265
\FBo@ht=\skip163
\FBc@ht=\skip164
\FBf@ht=\skip165
\FBo@max=\skip166
\FBc@max=\skip167
\FBf@max=\skip168
\c@FBl@b=\count324
\floatbox@depth=\count325
\c@FRobj=\count326
\c@FRsobj=\count327
\Xhsize=\skip169
\sXhsize=\skip170
\Zhsize=\skip171
\sZhsize=\skip172
\flrow@rowbox=\box77
\FR@Zunitlength=\dimen266
\c@FBcnt=\count328
\FPOScnt=\count329
\LTleft=\skip173
\LTright=\skip174
```

```
\LTleft=\skip175
\LTright=\skip176
\flrow@types=\toks41
)
Package translations Info: No language package found. I am going to use
`englis
h' as default language. on input line 71.
LaTeX Font Info:    Trying to load font information for T1+Merriwthr-OsF
on inp
ut line 71.
(c:/TeXLive/2022/texmf-dist/tex/latex/merriweather/T1Merriwthr-OsF.fd
File: T1Merriwthr-OsF.fd 2020/08/30 (autoinst) Font definitions for
T1/Merriwth
r-OsF.
)
LaTeX Font Info:    Font shape `T1/Merriwthr-OsF/m/n' will be
(Font)              scaled to size 7.5pt on input line 71.
(./main.aux)
\openout1 = `main.aux'.

LaTeX Font Info:    Checking defaults for OML/cmm/m/it on input line 71.
LaTeX Font Info:    ... okay on input line 71.
LaTeX Font Info:    Checking defaults for OMS/cmsy/m/n on input line 71.
LaTeX Font Info:    ... okay on input line 71.
LaTeX Font Info:    Checking defaults for OT1/cmr/m/n on input line 71.
LaTeX Font Info:    ... okay on input line 71.
LaTeX Font Info:    Checking defaults for T1/cmr/m/n on input line 71.
LaTeX Font Info:    ... okay on input line 71.
LaTeX Font Info:    Checking defaults for TS1/cmr/m/n on input line 71.
LaTeX Font Info:    ... okay on input line 71.
LaTeX Font Info:    Checking defaults for OMX/cmex/m/n on input line 71.
LaTeX Font Info:    ... okay on input line 71.
LaTeX Font Info:    Checking defaults for U/cmr/m/n on input line 71.
LaTeX Font Info:    ... okay on input line 71.
LaTeX Font Info:    Checking defaults for PD1/pdf/m/n on input line 71.
LaTeX Font Info:    ... okay on input line 71.
LaTeX Font Info:    Checking defaults for PU/pdf/m/n on input line 71.
LaTeX Font Info:    ... okay on input line 71.
LaTeX Info: Redefining \microtypecontext on input line 71.
Package microtype Info: Applying patch `item' on input line 71.
Package microtype Info: Applying patch `toc' on input line 71.
Package microtype Info: Applying patch `eqnum' on input line 71.

Package microtype Warning: Unable to apply patch `footnote' on input line
71.

Package microtype Info: Applying patch `verbatim' on input line 71.
Package microtype Info: Generating PDF output.
Package microtype Info: Character protrusion enabled (level 2).
Package microtype Info: Using default protrusion set `alltext'.
Package microtype Info: Automatic font expansion enabled (level 2),
(microtype)             stretch: 20, shrink: 20, step: 1, non-selected.
Package microtype Info: Using default expansion set `alltext-nott'.
LaTeX Info: Redefining \showhyphens on input line 71.
```

```
Package microtype Info: No adjustment of tracking.
Package microtype Info: No adjustment of interword spacing.
Package microtype Info: No adjustment of character kerning.
Package microtype Info: Loading generic protrusion settings for font
family
(microtype)                `Merriwthr-OsF' (encoding: T1).
(microtype)                For optimal results, create family-specific
settings.
(microtype)                See the microtype manual for details.
LaTeX Font Info:    Redeclaring symbol font `operators' on input line 71.
LaTeX Font Info:    Encoding `OT1' has changed to `T1' for symbol font
(Font)              `operators' in the math version `normal' on input
line 71.
LaTeX Font Info:    Overwriting symbol font `operators' in version
`normal'
(Font)                  OT1/cmr/m/n --> T1/Merriwthr-OsF/m/up on input
line 71.

LaTeX Font Info:    Encoding `OT1' has changed to `T1' for symbol font
(Font)              `operators' in the math version `bold' on input line
71.
LaTeX Font Info:    Overwriting symbol font `operators' in version `bold'
(Font)                  OT1/cmr/bx/n --> T1/Merriwthr-OsF/m/up on input
line 71
.
LaTeX Font Info:    Overwriting symbol font `operators' in version `bold'
(Font)                  T1/Merriwthr-OsF/m/up --> T1/Merriwthr-OsF/b/up
on inpu
t line 71.
LaTeX Font Info:    Redeclaring math alphabet \mathbf on input line 71.
LaTeX Font Info:    Overwriting math alphabet `\mathbf' in version
`normal'
(Font)                  OT1/cmr/bx/n --> T1/Merriwthr-OsF/b/up on input
line 71
.
LaTeX Font Info:    Overwriting math alphabet `\mathbf' in version `bold'
(Font)                  OT1/cmr/bx/n --> T1/Merriwthr-OsF/b/up on input
line 71
.
LaTeX Font Info:    Redeclaring math alphabet \mathsf on input line 71.
LaTeX Font Info:    Overwriting math alphabet `\mathsf' in version
`normal'
(Font)                  OT1/cmss/m/n --> T1/MerriwthrSans-OsF/m/up on
input lin
e 71.
LaTeX Font Info:    Overwriting math alphabet `\mathsf' in version `bold'
(Font)                  OT1/cmss/bx/n --> T1/MerriwthrSans-OsF/m/up on
input li
ne 71.
LaTeX Font Info:    Redeclaring math alphabet \mathit on input line 71.
LaTeX Font Info:    Overwriting math alphabet `\mathit' in version
`normal'
(Font)                  OT1/cmr/m/it --> T1/Merriwthr-OsF/m/it on input
line 71
```

```
.
LaTeX Font Info:    Overwriting math alphabet `\mathit' in version `bold'
(Font)                  OT1/cmr/bx/it --> T1/Merriwthr-OsF/m/it on input
line 7
1.
LaTeX Font Info:    Redeclaring math alphabet \mathtt on input line 71.
LaTeX Font Info:    Overwriting math alphabet `\mathtt' in version
`normal'
(Font)                  OT1/cmtt/m/n --> T1/lmtt/m/up on input line 71.
LaTeX Font Info:    Overwriting math alphabet `\mathtt' in version `bold'
(Font)                  OT1/cmtt/m/n --> T1/lmtt/m/up on input line 71.
LaTeX Font Info:    Overwriting math alphabet `\mathsf' in version `bold'
(Font)                  T1/MerriwthrSans-OsF/m/up --> T1/MerriwthrSans-
OsF/b/up
 on input line 71.
LaTeX Font Info:    Overwriting math alphabet `\mathit' in version `bold'
(Font)                  T1/Merriwthr-OsF/m/it --> T1/Merriwthr-OsF/b/it
on inpu
t line 71.
\c@mv@tabular=\count330
\c@mv@boldtabular=\count331
(c:/TeXLive/2022/texmf-dist/tex/context/base/mkii/supp-pdf.mkii
[Loading MPS to PDF converter (version 2006.09.02).]
\scratchcounter=\count332
\scratchdimen=\dimen267
\scratchbox=\box78
\nofMPsegments=\count333
\nofMParguments=\count334
\everyMPshowfont=\toks42
\MPscratchCnt=\count335
\MPscratchDim=\dimen268
\MPnumerator=\count336
\makeMPintoPDFobject=\count337
\everyMPtoPDFconversion=\toks43
) (c:/TeXLive/2022/texmf-dist/tex/latex/epstopdf-pkg/epstopdf-base.sty
Package: epstopdf-base 2020-01-24 v2.11 Base part for package epstopdf
Package epstopdf-base Info: Redefining graphics rule for `.eps' on input
line 4
85.
(c:/TeXLive/2022/texmf-dist/tex/latex/latexconfig/epstopdf-sys.cfg
File: epstopdf-sys.cfg 2010/07/13 v1.3 Configuration of (r)epstopdf for
TeX Liv
e
))
Package newfloat Info: `float' package detected.
*geometry* driver: auto-detecting
*geometry* detected driver: pdftex
*geometry* verbose mode - [ preamble ] result:
* driver: pdftex
* paper: a4paper
* layout: <same size as paper>
* layoutoffset:(h,v)=(0.0pt,0.0pt)
* modes: includefoot twoside
* h-part:(L,W,R)=(54.64pt, 488.22787pt, 54.64pt)
```

```
* v-part:(T,H,B)=(66.0pt, 745.04684pt, 34.0pt)
* \paperwidth=597.50787pt
* \paperheight=845.04684pt
* \textwidth=488.22787pt
* \textheight=715.04684pt
* \oddsidemargin=-17.62999pt
* \evensidemargin=-17.62999pt
* \topmargin=-47.76999pt
* \headheight=17.5pt
* \headsep=24.0pt
* \topskip=10.0pt
* \footskip=30.0pt
* \marginparwidth=48.0pt
* \marginparsep=10.0pt
* \columnsep=18.0pt
* \skip\footins=22.0pt plus 2.0pt
* \hoffset=0.0pt
* \voffset=0.0pt
* \mag=1000
* \@twocolumntrue
* \@twosidetrue
* \@mparswitchtrue
* \@reversemarginfalse
* (1in=72.27pt=25.4mm, 1cm=28.453pt)

Package hyperref Info: Link coloring ON on input line 71.
(./main.out) (./main.out)
\@outlinefile=\write3
\openout3 = `main.out'.

\@gscitedetails=\box79
\@gscitedetailsheight=\skip177
\@gsheadbox=\box80
\@gsheadboxheight=\skip178
LaTeX Font Info:    Font shape `T1/Merriwthr-OsF/b/n' will be
(Font)              scaled to size 6.5pt on input line 71.
LaTeX Font Info:    Calculating math sizes for size <7.5> on input line
71.

LaTeX Font Warning: Font shape `T1/Merriwthr-OsF/m/up' undefined
(Font)              using `T1/Merriwthr-OsF/m/n' instead on input line
71.

LaTeX Font Info:    Font shape `T1/Merriwthr-OsF/m/up' will be
(Font)              scaled to size 6.24973pt on input line 71.
LaTeX Font Info:    Font shape `T1/Merriwthr-OsF/m/up' will be
(Font)              scaled to size 5.24997pt on input line 71.
LaTeX Font Info:    Trying to load font information for U+eur on input
line 71.

(c:/TeXLive/2022/texmf-dist/tex/latex/amsfonts/ueur.fd
File: ueur.fd 2013/01/14 v3.01 Euler Roman
) (c:/TeXLive/2022/texmf-dist/tex/latex/microtype/mt-eur.cfg
```

```
File: mt-eur.cfg 2006/07/31 v1.1 microtype config. file: AMS Euler Roman
(RS)
)

LaTeX Font Warning: Font shape `OMS/cmsy/m/n' in size <7.5> not available
(Font)              size <7> substituted on input line 71.

LaTeX Font Info:    External font `cmex10' loaded for size
(Font)              <7.5> on input line 71.
LaTeX Font Info:    External font `cmex10' loaded for size
(Font)              <6.24973> on input line 71.
LaTeX Font Info:    External font `cmex10' loaded for size
(Font)              <5.24997> on input line 71.
LaTeX Font Info:    Trying to load font information for U+euf on input
line 71.

(c:/TeXLive/2022/texmf-dist/tex/latex/amsfonts/ueuf.fd
File: ueuf.fd 2013/01/14 v3.01 Euler Fraktur
) (c:/TeXLive/2022/texmf-dist/tex/latex/microtype/mt-euf.cfg
File: mt-euf.cfg 2006/07/03 v1.1 microtype config. file: AMS Euler
Fraktur (RS)

)
LaTeX Font Info:    Trying to load font information for U+eus on input
line 71.

(c:/TeXLive/2022/texmf-dist/tex/latex/amsfonts/ueus.fd
File: ueus.fd 2013/01/14 v3.01 Euler Script
) (c:/TeXLive/2022/texmf-dist/tex/latex/microtype/mt-eus.cfg
File: mt-eus.cfg 2006/07/28 v1.2 microtype config. file: AMS Euler Script
(RS)
)
LaTeX Font Info:    Trying to load font information for U+euex on input
line 71
.
(c:/TeXLive/2022/texmf-dist/tex/latex/amsfonts/ueuex.fd
File: ueuex.fd 2013/01/14 v3.01 Euler extra symbols
)

LaTeX Font Warning: Font shape `OML/cmm/m/it' in size <7.5> not available
(Font)              size <7> substituted on input line 71.

LaTeX Font Info:    Font shape `T1/Merriwthr-OsF/m/n' will be
(Font)              scaled to size 6.24973pt on input line 71.
LaTeX Font Info:    Font shape `T1/Merriwthr-OsF/m/n' will be
(Font)              scaled to size 5.24997pt on input line 71.
LaTeX Font Info:    Font shape `T1/Merriwthr-OsF/m/it' will be
(Font)              scaled to size 7.5pt on input line 71.
LaTeX Font Info:    Font shape `T1/Merriwthr-OsF/m/it' will be
(Font)              scaled to size 6.24973pt on input line 71.
LaTeX Font Info:    Font shape `T1/Merriwthr-OsF/m/it' will be
(Font)              scaled to size 5.24997pt on input line 71.
LaTeX Font Info:    Font shape `T1/Merriwthr-OsF/m/n' will be
(Font)              scaled to size 8.0pt on input line 71.
```

```
LaTeX Font Info:    Font shape `T1/Merriwthr-OsF/m/it' will be
(Font)              scaled to size 8.0pt on input line 71.
LaTeX Font Info:    Font shape `T1/Merriwthr-OsF/b/it' will be
(Font)              scaled to size 8.0pt on input line 71.
Package caption Info: Begin \AtBeginDocument code.
Package caption Info: float package is loaded.
Package caption Info: floatrow package is loaded.
Package caption Info: End \AtBeginDocument code.

(c:/TeXLive/2022/texmf-dist/tex/latex/translations/translations-basic-
dictionar
y-english.trsl
File: translations-basic-dictionary-english.trsl (english translation
file `tra
nslations-basic-dictionary')
)
Package translations Info: loading dictionary `translations-basic-
dictionary' f
or `english'. on input line 71.
TextBlockOrigin set to 4pc+6.64pt x 4pc+6pt
<oup.pdf, id=97, 49.18375pt x 48.18pt>
File: oup.pdf Graphic file (type pdf)
<use oup.pdf>
Package pdftex.def Info: oup.pdf  used on input line 92.
(pdftex.def)             Requested size: 59.24683pt x 58.038pt.
<gigascience-logo.pdf, id=98, 99.37125pt x 33.12375pt>
File: gigascience-logo.pdf Graphic file (type pdf)
<use gigascience-logo.pdf>
Package pdftex.def Info: gigascience-logo.pdf  used on input line 92.
(pdftex.def)             Requested size: 126.00902pt x 42.0pt.

Overfull \hbox (54.64pt too wide) in paragraph at lines 92--92
[][]
 []

LaTeX Font Info:    Font shape `T1/Merriwthr-OsF/m/n' will be
(Font)              scaled to size 14.0pt on input line 92.
LaTeX Font Info:    Font shape `T1/Merriwthr-OsF/m/n' will be
(Font)              scaled to size 8.99997pt on input line 92.
LaTeX Font Info:    Calculating math sizes for size <14> on input line
92.
LaTeX Font Info:    Font shape `T1/Merriwthr-OsF/m/up' will be
(Font)              scaled to size 14.0pt on input line 92.
LaTeX Font Info:    Font shape `T1/Merriwthr-OsF/m/up' will be
(Font)              scaled to size 11.66617pt on input line 92.
LaTeX Font Info:    Font shape `T1/Merriwthr-OsF/m/up' will be
(Font)              scaled to size 9.79996pt on input line 92.
LaTeX Font Info:    External font `cmex10' loaded for size
(Font)              <14> on input line 92.
LaTeX Font Info:    External font `cmex10' loaded for size
(Font)              <11.66617> on input line 92.
LaTeX Font Info:    External font `cmex10' loaded for size
(Font)              <9.79996> on input line 92.
LaTeX Font Info:    Font shape `T1/Merriwthr-OsF/m/n' will be
```

```
(Font)              scaled to size 11.66617pt on input line 92.
LaTeX Font Info:    Font shape `T1/Merriwthr-OsF/m/n' will be
(Font)              scaled to size 9.79996pt on input line 92.
LaTeX Font Info:    Font shape `T1/Merriwthr-OsF/m/it' will be
(Font)              scaled to size 14.0pt on input line 92.
LaTeX Font Info:    Font shape `T1/Merriwthr-OsF/m/it' will be
(Font)              scaled to size 11.66617pt on input line 92.
LaTeX Font Info:    Font shape `T1/Merriwthr-OsF/m/it' will be
(Font)              scaled to size 9.79996pt on input line 92.
LaTeX Font Info:    Font shape `T1/Merriwthr-OsF/b/n' will be
(Font)              scaled to size 18.0pt on input line 92.
LaTeX Font Info:    Font shape `T1/Merriwthr-OsF/m/n' will be
(Font)              scaled to size 13.0pt on input line 92.
LaTeX Font Info:    Calculating math sizes for size <13> on input line
92.
LaTeX Font Info:    Font shape `T1/Merriwthr-OsF/m/up' will be
(Font)              scaled to size 13.0pt on input line 92.
LaTeX Font Info:    Font shape `T1/Merriwthr-OsF/m/up' will be
(Font)              scaled to size 10.83287pt on input line 92.
LaTeX Font Info:    Font shape `T1/Merriwthr-OsF/m/up' will be
(Font)              scaled to size 9.09996pt on input line 92.

LaTeX Font Warning: Font shape `OMS/cmsy/m/n' in size <13> not available
(Font)              size <12> substituted on input line 92.

LaTeX Font Info:    External font `cmex10' loaded for size
(Font)              <13> on input line 92.
LaTeX Font Info:    External font `cmex10' loaded for size
(Font)              <10.83287> on input line 92.
LaTeX Font Info:    External font `cmex10' loaded for size
(Font)              <9.09996> on input line 92.

LaTeX Font Warning: Font shape `OML/cmm/m/it' in size <13> not available
(Font)              size <12> substituted on input line 92.

LaTeX Font Info:    Font shape `T1/Merriwthr-OsF/m/n' will be
(Font)              scaled to size 10.83287pt on input line 92.
LaTeX Font Info:    Font shape `T1/Merriwthr-OsF/m/n' will be
(Font)              scaled to size 9.09996pt on input line 92.
LaTeX Font Info:    Font shape `T1/Merriwthr-OsF/m/it' will be
(Font)              scaled to size 13.0pt on input line 92.
LaTeX Font Info:    Font shape `T1/Merriwthr-OsF/m/it' will be
(Font)              scaled to size 10.83287pt on input line 92.
LaTeX Font Info:    Font shape `T1/Merriwthr-OsF/m/it' will be
(Font)              scaled to size 9.09996pt on input line 92.
LaTeX Font Info:    Trying to load font information for TS1+Merriwthr-OsF
on in
put line 92.
(c:/TeXLive/2022/texmf-dist/tex/latex/merriweather/TS1Merriwthr-OsF.fd
File: TS1Merriwthr-OsF.fd 2020/08/30 (autoinst) Font definitions for
TS1/Merriw
thr-OsF.
)
LaTeX Font Info:    Font shape `TS1/Merriwthr-OsF/m/n' will be
```

```
(Font)              scaled to size 10.83287pt on input line 92.
Package microtype Info: Loading generic protrusion settings for font
family
(microtype)                `Merriwthr-OsF' (encoding: TS1).
(microtype)                For optimal results, create family-specific
settings.
(microtype)                See the microtype manual for details.
LaTeX Font Info:    Font shape `T1/Merriwthr-OsF/m/n' will be
(Font)              scaled to size 9.0pt on input line 92.
LaTeX Font Info:    Font shape `T1/Merriwthr-OsF/m/up' will be
(Font)              scaled to size 9.0pt on input line 92.
LaTeX Font Info:    Font shape `T1/Merriwthr-OsF/m/up' will be
(Font)              scaled to size 7.0pt on input line 92.
LaTeX Font Info:    Font shape `T1/Merriwthr-OsF/m/up' will be
(Font)              scaled to size 5.0pt on input line 92.
LaTeX Font Info:    External font `cmex10' loaded for size
(Font)              <9> on input line 92.
LaTeX Font Info:    External font `cmex10' loaded for size
(Font)              <7> on input line 92.
LaTeX Font Info:    External font `cmex10' loaded for size
(Font)              <5> on input line 92.
LaTeX Font Info:    Font shape `T1/Merriwthr-OsF/m/n' will be
(Font)              scaled to size 7.0pt on input line 92.
LaTeX Font Info:    Font shape `T1/Merriwthr-OsF/m/n' will be
(Font)              scaled to size 5.0pt on input line 92.
LaTeX Font Info:    Font shape `T1/Merriwthr-OsF/m/it' will be
(Font)              scaled to size 9.0pt on input line 92.
LaTeX Font Info:    Font shape `T1/Merriwthr-OsF/m/it' will be
(Font)              scaled to size 7.0pt on input line 92.
LaTeX Font Info:    Font shape `T1/Merriwthr-OsF/m/it' will be
(Font)              scaled to size 5.0pt on input line 92.
LaTeX Font Info:    Font shape `T1/Merriwthr-OsF/m/n' will be
(Font)              scaled to size 6.5pt on input line 92.
LaTeX Font Info:    Calculating math sizes for size <6.5> on input line
92.
LaTeX Font Info:    Font shape `T1/Merriwthr-OsF/m/up' will be
(Font)              scaled to size 6.5pt on input line 92.
LaTeX Font Info:    Font shape `T1/Merriwthr-OsF/m/up' will be
(Font)              scaled to size 5.41643pt on input line 92.
LaTeX Font Info:    Font shape `T1/Merriwthr-OsF/m/up' will be
(Font)              scaled to size 4.54997pt on input line 92.

LaTeX Font Warning: Font shape `OMS/cmsy/m/n' in size <6.5> not available
(Font)              size <6> substituted on input line 92.


LaTeX Font Warning: Font shape `OMS/cmsy/m/n' in size <5.41643> not
available
(Font)              size <5> substituted on input line 92.


LaTeX Font Warning: Font shape `OMS/cmsy/m/n' in size <4.54997> not
available
(Font)              size <5> substituted on input line 92.
```

```
LaTeX Font Info:    External font `cmex10' loaded for size
(Font)              <6.5> on input line 92.
LaTeX Font Info:    External font `cmex10' loaded for size
(Font)              <5.41643> on input line 92.
LaTeX Font Info:    External font `cmex10' loaded for size
(Font)              <4.54997> on input line 92.

LaTeX Font Warning: Font shape `OML/cmm/m/it' in size <6.5> not available
(Font)              size <6> substituted on input line 92.


LaTeX Font Warning: Font shape `OML/cmm/m/it' in size <5.41643> not
available
(Font)              size <5> substituted on input line 92.


LaTeX Font Warning: Font shape `OML/cmm/m/it' in size <4.54997> not
available
(Font)              size <5> substituted on input line 92.

LaTeX Font Info:    Font shape `T1/Merriwthr-OsF/m/n' will be
(Font)              scaled to size 5.41643pt on input line 92.
LaTeX Font Info:    Font shape `T1/Merriwthr-OsF/m/n' will be
(Font)              scaled to size 4.54997pt on input line 92.
LaTeX Font Info:    Font shape `T1/Merriwthr-OsF/m/it' will be
(Font)              scaled to size 6.5pt on input line 92.
LaTeX Font Info:    Font shape `T1/Merriwthr-OsF/m/it' will be
(Font)              scaled to size 5.41643pt on input line 92.
LaTeX Font Info:    Font shape `T1/Merriwthr-OsF/m/it' will be
(Font)              scaled to size 4.54997pt on input line 92.
LaTeX Font Info:    Font shape `TS1/Merriwthr-OsF/m/n' will be
(Font)              scaled to size 5.41643pt on input line 92.

Overfull \hbox (54.64pt too wide) in paragraph at lines 92--92
[][][]
 []

LaTeX Font Info:    Font shape `T1/Merriwthr-OsF/b/n' will be
(Font)              scaled to size 10.0pt on input line 92.
LaTeX Font Info:    Font shape `T1/Merriwthr-OsF/b/n' will be
(Font)              scaled to size 8.0pt on input line 92.

Overfull \hbox (54.64pt too wide) in paragraph at lines 92--92
[][][]
 []

Package mdframed Info: mdframed works in twoside mode on input line 96.
LaTeX Font Info:    Font shape `T1/Merriwthr-OsF/b/n' will be
(Font)              scaled to size 8.2pt on input line 96.
LaTeX Font Info:    Font shape `TS1/Merriwthr-OsF/m/n' will be
(Font)              scaled to size 7.5pt on input line 98.
Package mdframed Info: mdframed inside float
 mdframed uses option nobreak mdframed on input line 105.
```

```
Package mdframed Info: mdframed inside a box
 mdframed uses option nobreak mdframed on input line 105.
LaTeX Font Info:    Font shape `T1/Merriwthr-OsF/b/n' will be
(Font)              scaled to size 8.5pt on input line 110.
LaTeX Font Info:    Font shape `T1/Merriwthr-OsF/b/n' will be
(Font)              scaled to size 7.5pt on input line 113.

Package natbib Warning: Citation `o2017dockstore' on page 1 undefined on
input
line 113.


Package natbib Warning: Citation `da2017biocontainers' on page 1
undefined on i
nput line 113.


Underfull \vbox (badness 10000) has occurred while \output is active []


Package natbib Warning: Citation `dai2012bioinformatics' on page 1
undefined on
 input line 117.


Package natbib Warning: Citation `schadt2010computational' on page 1
undefined
on input line 117.


Package natbib Warning: Citation `schadt2011cloud' on page 1 undefined on
input
 line 117.


Package natbib Warning: Citation `lau2017cancer' on page 1 undefined on
input l
ine 117.


Package natbib Warning: Citation `reynolds2017isb' on page 1 undefined on
input
 line 117.


Package natbib Warning: Citation `afgan2011harnessing' on page 1
undefined on i
nput line 117.


Package natbib Warning: Citation `birger2017firecloud' on page 1
undefined on i
nput line 117.
```

```
Underfull \vbox (badness 10000) has occurred while \output is active []

LaTeX Font Info:    Font shape `T1/Merriwthr-OsF/m/n' will be
(Font)              scaled to size 7.8pt on input line 118.
LaTeX Font Info:    Font shape `T1/Merriwthr-OsF/b/n' will be
(Font)              scaled to size 7.8pt on input line 118.
[1{c:/TeXLive/2022/texmf-var/fonts/map/pdftex/updmap/pdftex.map}


 <./oup.pdf> <./gigascience-logo.pdf>]

Package natbib Warning: Citation `tatlow2016cloud' on page 2 undefined on
input
 line 143.


Package natbib Warning: Citation `lachmann2018massive' on page 2
undefined on i
nput line 143.


Package natbib Warning: Citation `tatlow2016cloud' on page 2 undefined on
input
 line 143.


Package natbib Warning: Citation `juve2013characterizing' on page 2
undefined o
n input line 143.


Package natbib Warning: Citation `tyryshkina2019predicting' on page 2
undefined
 on input line 145.


Package natbib Warning: Citation `weingartner2015cloud' on page 2
undefined on
input line 148.


Package natbib Warning: Citation `gregg2013thinking' on page 2 undefined
on inp
ut line 148.


Package natbib Warning: Citation `lloyd2014virtual' on page 2 undefined
on inpu
t line 148.


Package natbib Warning: Citation `lloyd2015demystifying' on page 2
undefined on
```

```
 input line 148.


Underfull \vbox (badness 3260) has occurred while \output is active []

LaTeX Font Info:    Font shape `T1/Merriwthr-OsF/m/it' will be
(Font)              scaled to size 7.8pt on input line 149.
[2]

Package natbib Warning: Citation `cp13' on page 3 undefined on input line
150.


Package natbib Warning: Citation `cp14' on page 3 undefined on input line
152.


Package natbib Warning: Citation `ji2019cmonitor' on page 3 undefined on
input
line 152.


Package natbib Warning: Citation `arm1' on page 3 undefined on input line
152.


Package natbib Warning: Citation `arm2' on page 3 undefined on input line
152.


Package natbib Warning: Citation `arm3' on page 3 undefined on input line
152.

LaTeX Font Info:    Trying to load font information for T1+lmtt on input
line 1
61.
(c:/TeXLive/2022/texmf-dist/tex/latex/lm/t1lmtt.fd
File: t1lmtt.fd 2015/05/01 v1.6.1 Font defs for Latin Modern
)
Package microtype Info: Loading generic protrusion settings for font
family
(microtype)             `lmtt' (encoding: T1).
(microtype)             For optimal results, create family-specific
settings.
(microtype)             See the microtype manual for details.

Package natbib Warning: Citation `psutil' on page 3 undefined on input
line 161
.

LaTeX Font Info:    Font shape `T1/Merriwthr-OsF/m/n' will be
(Font)              scaled to size 6.0pt on input line 165.
LaTeX Font Info:    Font shape `T1/Merriwthr-OsF/b/n' will be
(Font)              scaled to size 6.0pt on input line 165.
```

```
LaTeX Font Info:    Font shape `T1/Merriwthr-OsF/m/it' will be
(Font)              scaled to size 6.0pt on input line 165.
<MetricAssociation.png, id=125, 1076.02pt x 781.92125pt>
File: MetricAssociation.png Graphic file (type png)
<use MetricAssociation.png>
Package pdftex.def Info: MetricAssociation.png  used on input line 168.
(pdftex.def)             Requested size: 451.92532pt x 328.40468pt.
LaTeX Font Info:    Font shape `T1/Merriwthr-OsF/b/n' will be
(Font)              scaled to size 7.0pt on input line 176.

Package natbib Warning: Citation `cp12' on page 3 undefined on input line
261.


Package natbib Warning: Citation `cp12' on page 3 undefined on input line
261.


Package natbib Warning: Citation `cp1' on page 3 undefined on input line
261.


Package natbib Warning: Citation `cp2' on page 3 undefined on input line
261.


Package natbib Warning: Citation `cp3' on page 3 undefined on input line
261.


Package natbib Warning: Citation `cp4' on page 3 undefined on input line
261.


Package natbib Warning: Citation `cp5' on page 3 undefined on input line
261.


Package natbib Warning: Citation `cp6' on page 3 undefined on input line
261.


Package natbib Warning: Citation `cp7' on page 3 undefined on input line
261.


Package natbib Warning: Citation `cp8' on page 3 undefined on input line
261.


Package natbib Warning: Citation `cp9' on page 3 undefined on input line
261.
```

```
Package natbib Warning: Citation `cp10' on page 3 undefined on input line
261.


Package natbib Warning: Citation `cp11' on page 3 undefined on input line
261.


Package natbib Warning: Citation `cgroups' on page 3 undefined on input
line 26
3.


Package natbib Warning: Citation `umi-xiong' on page 3 undefined on input
line
284.


Package natbib Warning: Citation `Soumillon003236' on page 3 undefined on
input
 line 284.


Package natbib Warning: Citation `Soumillon003236' on page 3 undefined on
input
 line 284.


Package natbib Warning: Citation `hung2019holistic' on page 3 undefined
on inpu
t line 284.

[3]
Underfull \vbox (badness 10000) has occurred while \output is active []

<fig2-new-e.png, id=139, 1365.1pt x 824.07875pt>
File: fig2-new-e.png Graphic file (type png)
<use fig2-new-e.png>
Package pdftex.def Info: fig2-new-e.png  used on input line 292.
(pdftex.def)             Requested size: 241.62477pt x 145.86319pt.

Overfull \hbox (6.51083pt too wide) in paragraph at lines 292--294
 [][]
 []


LaTeX Warning: `!h' float specifier changed to `!ht'.


Underfull \vbox (badness 2809) has occurred while \output is active []

[4 <./MetricAssociation.png>]
<figure3.png, id=145, 528.0528pt x 431.4519pt>
File: figure3.png Graphic file (type png)
```

```
<use figure3.png>
Package pdftex.def Info: figure3.png  used on input line 302.
(pdftex.def)             Requested size: 448.847pt x 366.73586pt.
LaTeX Font Info:    Font shape `T1/Merriwthr-OsF/b/it' will be
(Font)              scaled to size 8.5pt on input line 308.

Underfull \vbox (badness 10000) has occurred while \output is active []

[5 <./fig2-new-e.png>]
Underfull \vbox (badness 10000) has occurred while \output is active []

[6 <./figure3.png>]
LaTeX Font Info:    Font shape `T1/Merriwthr-OsF/m/up' will be
(Font)              scaled to size 6.0pt on input line 332.
LaTeX Font Info:    External font `cmex10' loaded for size
(Font)              <6> on input line 332.
<figure4-new-merged.png, id=163, 156.1032pt x 185.7339pt>
File: figure4-new-merged.png Graphic file (type png)
<use figure4-new-merged.png>
Package pdftex.def Info: figure4-new-merged.png  used on input line 338.
(pdftex.def)             Requested size: 234.15419pt x 278.60016pt.

Underfull \vbox (badness 10000) has occurred while \output is active []

<new-figure-5-without-vm.png, id=164, 823.659pt x 350.4pt>
File: new-figure-5-without-vm.png Graphic file (type png)
<use new-figure-5-without-vm.png>
Package pdftex.def Info: new-figure-5-without-vm.png  used on input line
353.
(pdftex.def)             Requested size: 235.11394pt x 100.02008pt.
<cp.png, id=166, 603.25375pt x 332.24126pt>
File: cp.png Graphic file (type png)
<use cp.png>
Package pdftex.def Info: cp.png  used on input line 386.
(pdftex.def)             Requested size: 241.29723pt x 132.89413pt.

Overfull \hbox (6.18329pt too wide) in paragraph at lines 386--389
 [][]
 []


LaTeX Warning: `!h' float specifier changed to `!ht'.


Underfull \vbox (badness 10000) has occurred while \output is active []

[7 <./figure4-new-merged.png> <./new-figure-5-without-vm.png>]
Underfull \vbox (badness 3668) has occurred while \output is active []

[8 <./cp.png>]

Package natbib Warning: Citation `ContainerProfilerGigaDB' on page 9
undefined
on input line 466.
```

```
Package natbib Warning: Citation `xiong2017comparison' on page 9
undefined on i
nput line 466.

LaTeX Font Info:    Font shape `T1/Merriwthr-OsF/m/up' will be
(Font)              scaled to size 7.5pt on input line 472.

Underfull \hbox (badness 10000) in paragraph at lines 472--473
[]\T1/Merriwthr-OsF/m/up/7.5 (+20) Project home page:
[][]$\T1/lmtt/m/n/7.5 htt
ps : / / github . com / wlloyduw /
 []

(./main.bbl [9]
Underfull \hbox (badness 5908) in paragraph at lines 190--193
\T1/Merriwthr-OsF/m/up/7.5 (+20) 02/2023).  [][]$\T1/lmtt/m/n/7.5 http :
/ / ww
w . linuxhowtos . org / System / procstat .
 []


Underfull \vbox (badness 10000) has occurred while \output is active []


Underfull \hbox (badness 10000) in paragraph at lines 200--204
\T1/lmtt/m/n/7.5 com / documentation / en-[]us / red _ hat _ enterprise _
linux
 / 6 /
 []


Underfull \hbox (badness 10000) in paragraph at lines 212--216
\T1/lmtt/m/n/7.5 redhat . com / documentation / en-[]us / red _ hat _
enterpris
e _
 []


Underfull \hbox (badness 10000) in paragraph at lines 218--222
\T1/lmtt/m/n/7.5 com / documentation / en-[]us / red _ hat _ enterprise _
linux
 / 6 /
 []


Underfull \hbox (badness 1565) in paragraph at lines 224--228
[]\T1/Merriwthr-OsF/m/up/7.5 (+20) /proc/loadavg (E.2.15.), Red Hat En-
ter-pris
e Linux 6, Red
 []
```

```
Underfull \hbox (badness 10000) in paragraph at lines 224--228
\T1/lmtt/m/n/7.5 redhat . com / documentation / en-[]us / red _ hat _
enterpris
e _
 []


Underfull \hbox (badness 10000) in paragraph at lines 230--234
\T1/lmtt/m/n/7.5 com / documentation / en-[]us / red _ hat _ enterprise _
linux
 / 6 /
 []


Underfull \hbox (badness 10000) in paragraph at lines 236--241
\T1/lmtt/m/n/7.5 red _ hat _ enterprise _ linux / 6 / html / resource _
managem
ent _
 []


Underfull \hbox (badness 1406) in paragraph at lines 243--247
[]\T1/Merriwthr-OsF/m/up/7.5 (+20) /cgroup/memory (3.7.), Red Hat En-ter-
prise
Linux 6, Red
 []


Underfull \hbox (badness 10000) in paragraph at lines 243--247
\T1/lmtt/m/n/7.5 redhat . com / documentation / en-[]us / red _ hat _
enterpris
e _
 []


Underfull \hbox (badness 10000) in paragraph at lines 249--253
\T1/lmtt/m/n/7.5 com / documentation / en-[]us / red _ hat _ enterprise _
linux
 / 6 /
 []


! LaTeX Error: Unicode character ′ (U+2032)
               not set up for use with LaTeX.

See the LaTeX manual or LaTeX Companion for explanation.
Type  H <return>  for immediate help.
 ...

l.303 ...NA sequencing with random primed and 3′
                                                  -directed
You may provide a definition with
\DeclareUnicodeCharacter
```

```
)

Package natbib Warning: There were undefined citations.

[10]
enddocument/afterlastpage: lastpage setting LastPage.
(./main.aux

Package natbib Warning: Citation(s) may have changed.
(natbib)                Rerun to get citations correct.

)

LaTeX Font Warning: Size substitutions with differences
(Font)              up to 1.0pt have occurred.


LaTeX Font Warning: Some font shapes were not available, defaults
substituted.


LaTeX Warning: Label(s) may have changed. Rerun to get cross-references
right.

Package rerunfilecheck Info: File `main.out' has not changed.
(rerunfilecheck)             Checksum:
5E44E32F8FB08A380D3FF57F646C6670;4659.
 )
Here is how much of TeX's memory you used:
 24708 strings out of 476024
 480686 string characters out of 5794017
 1914382 words of memory out of 5000000
 43985 multiletter control sequences out of 15000+600000
 1827106 words of font info for 597 fonts, out of 8000000 for 9000
 1141 hyphenation exceptions out of 8191
 123i,12n,131p,2320b,1063s stack positions out of
10000i,1000n,20000p,200000b,200000s
{c:/TeXLive/2022/texmf-dist/fonts/enc/dvips/lm/lm-
ec.enc}{c:/TeXLive/2022/tex
mf-
dist/fonts/enc/dvips/merriweather/merriwthr_posqbl.enc}{c:/TeXLive/2022/t
exm
f-
dist/fonts/enc/dvips/merriweather/merriwthr_owzwzj.enc}<c:/TeXLive/2022/t
exmf
-dist/fonts/type1/sorkin/merriweather/Merriwthr-
Bold.pfb><c:/TeXLive/2022/texmf
-dist/fonts/type1/sorkin/merriweather/Merriwthr-
BoldItalic.pfb><c:/TeXLive/2022
/texmf-dist/fonts/type1/sorkin/merriweather/Merriwthr-
Italic.pfb><c:/TeXLive/20
22/texmf-dist/fonts/type1/sorkin/merriweather/Merriwthr-
Regular.pfb><c:/TeXLive
```

```
/2022/texmf-
dist/fonts/type1/public/amsfonts/cm/cmsy7.pfb><c:/TeXLive/2022/texm
f-dist/fonts/type1/public/lm/lmtt8.pfb>
Output written on main.pdf (10 pages, 1224322 bytes).
PDF statistics:
 336 PDF objects out of 1000 (max. 8388607)
 292 compressed objects within 3 object streams
 88 named destinations out of 1000 (max. 500000)
 203489 words of extra memory for PDF output out of 221844 (max.
10000000)
```

Click here to access/download;LaTeX - Figure (eps, ps, etc.);ContainerProfiler-Performance-cost7.png

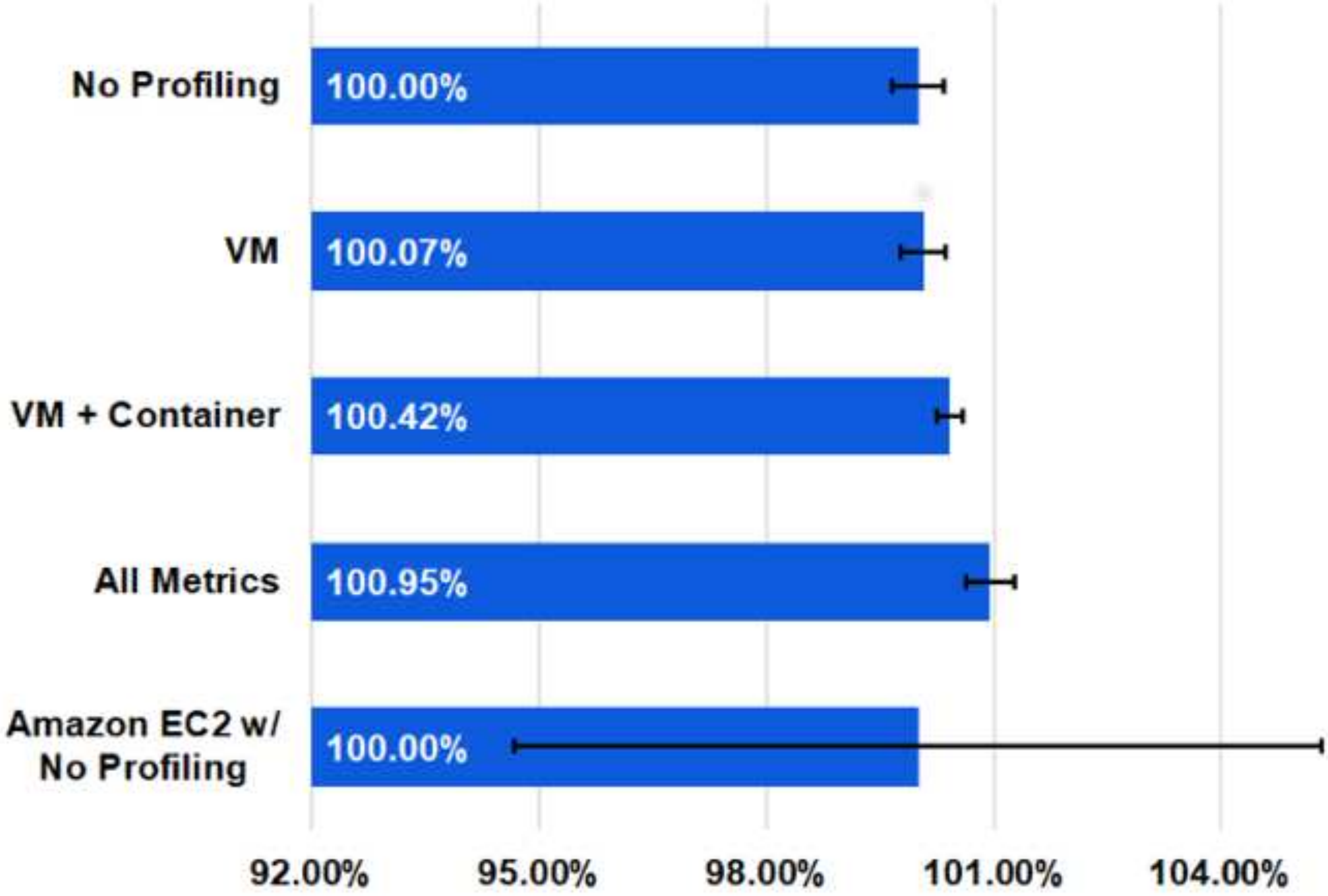

 Click here to access/download;LaTeX - Figure (eps, ps, etc.);profiler_figure.png

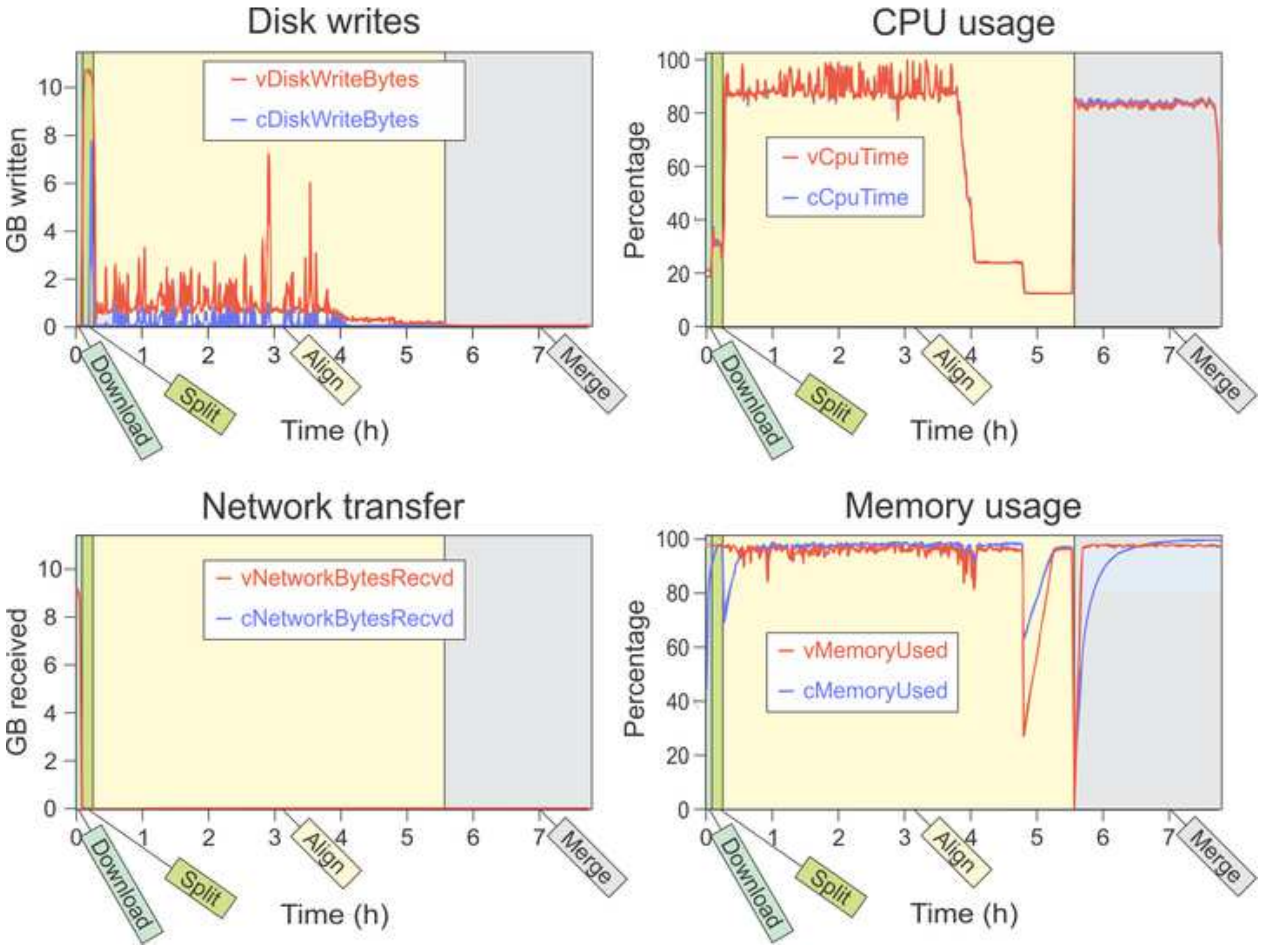

Click here to access/download;LaTeX - Figure (eps, ps, etc.);RNAseq-RU_graph_11.png

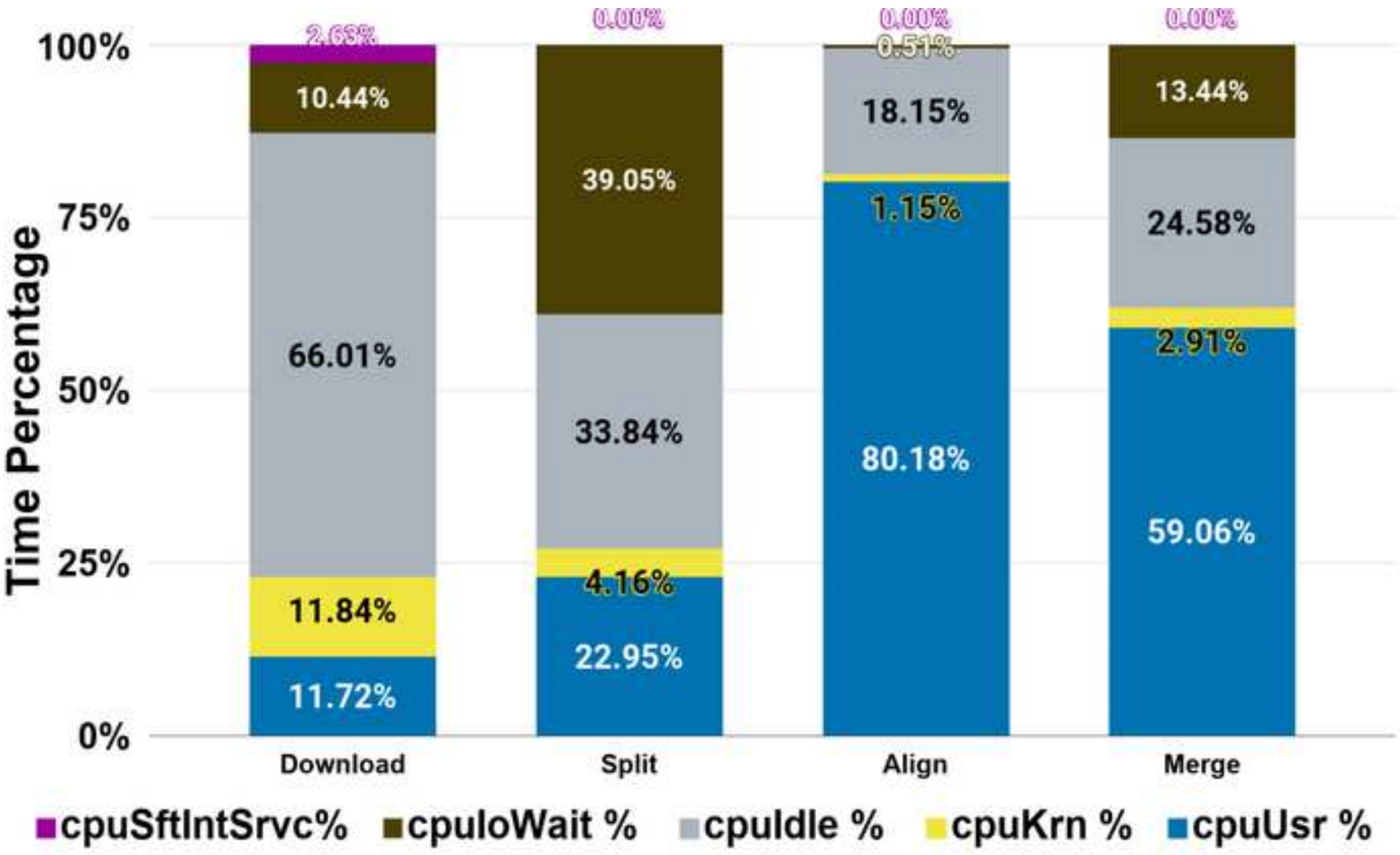

Manuscript

Click here to access/download;Manuscript;ContainerProfiler_Gigascience_T

OXFORD (GIGA)n SCIENCE

GigaScience, 2023, 1–11

doi: xx.xxxx/xxxx
Manuscript in Preparation
Paper

PAPER

Container Profiler: Profiling Resource Utilization of Containerized Big Data Pipelines

Varik Hoang[1,*,2], Ling-Hong Hung[1,*,3], David Perez[1], Huazeng Deng[1], Raymond Schooley[1,4], Niharika Arumilli[1], Ka Yee Yeung[1,5] and Wes Lloyd[1,†,6]

[1]School of Engineering and Technology, University of Washington Tacoma and [2]ORCID 0000-0001-7311-1308 and [3]ORCID 0000-0002-5209-2248 and [4]ORCID 0009-0009-1958-9525 and [5]ORCID 0000-0002-1754-7577 and [6]ORCID 0000-0003-2021-8501

[*]Contributed equally.
[†]wlloyd@uw.edu

Abstract

Background This paper presents the *Container Profiler*, a software tool that measures and records the resource usage of any containerized task. Our tool profiles the CPU, memory, disk, and network utilization of containerized tasks collecting over sixty Linux operating system metrics at the virtual machine, container, and process levels. The *Container Profiler* supports performing time series profiling at a configurable sampling interval to enable continuous monitoring of the resources consumed by containerized tasks and pipelines.
Results To investigate the utility of the *Container Profiler*, we profile the resource utilization requirements of a multi-stage bioinformatics analytical pipeline (RNA sequencing using unique molecular identifiers). We examine profiling metrics to assess patterns of CPU, disk, and network resource utilization across the different stages of the pipeline. We also quantify the profiling overhead of our Container Profiler tool to assess the impact of profiling a running pipeline with different levels of profiling granularity verifying that impacts are negligible.
Conclusions The *Container Profiler* provides a useful tool that can be used to continuously monitor the resource consumption of long and complex containerized applications that run locally or on the cloud. This can help identify bottlenecks where more resources are needed to improve performance.

Key words: Resource profiling; performance; testing; cloud computing; RNA sequencing

Findings

Background

Large-scale and diverse biomedical data have been generated to advance the understanding of biological mechanisms. Interpreting these data typically includes multiple analytical steps, each of which consists of different computational methods and software tools. An analytical *pipeline* (or *workflow*) is a sequence of computational tasks used to process and analyze specific biomedical data. Each analytical step in a pipeline can potentially require a different set of applications, libraries, and software dependencies. As a result, software containers that encapsulate executables with their dependencies have become popular to facilitate the deployment of complicated pipelines and to enhance their reproducibility [1, 2]. Additionally, different analytical steps in a pipeline could have different computing resource requirements. In particular, many bioinformatics pipelines consist of one or more computationally intensive steps stemming from their operation on large datasets requiring significant CPU, memory, network, and disk resources. As an example, the alignment step in a RNA sequencing pipeline

Compiled on: August 2, 2023.
Draft manuscript prepared by the author.

Key Points

- We present the *Container Profiler* a tool that enables profiling the resource utilization of any script or container-based task on Linux.
- The *Container Profiler* collects CPU, memory, disk, and network resource utilization metrics at the virtual machine, container, and process levels.
- The *Container Profiler* supports delta and time series resource utilization profiling at an adjustable time interval (e.g. 1-second) supporting monitoring and graphing of resource utilization enabling time series analysis to help identify performance bottlenecks for any Linux-based computational task.
- The *Container Profiler* can profile complex containerized computational jobs such as bioinformatics pipelines where multiple individual containers are used to implement specific steps.
- The *Container Profiler* is provided as a container which can merge with any existing container or used separately to profile independent Linux scripts or executables to characterize task resource utilization locally or on the cloud.
- We illustrate how different resources are required when performing different steps of a containerized pipeline analyzing unique molecular identifiers (UMI) RNA sequencing data.

typically requires relatively more CPU and memory resources than other steps, while the data download step typically requires more disk I/O and network resources.

Cloud computing has emerged as a solution that can provide the necessary resources needed for computationally intensive bioinformatics analyses [3, 4, 5, 6, 7, 8, 9]. However, deployment of analytical pipelines using Infrastructure-as-a-Service (IaaS) cloud platforms requires selecting the appropriate type and quantity of virtual machines (VMs) to address performance goals while balancing hosting costs. Cloud resource type selection is presently complicated by the rapidly growing number of available VM instance types and pricing models offered by public cloud providers. For example, the Amazon, Microsoft, and Google public clouds presently offer hundreds of different VM types under different pricing models. Further, Google allows users to create custom VM types with unique combinations of CPUs, memory, and disk capacity. These cloud VMs are available directly, or through various container platforms. Determining the best cloud deployment requires understanding the resource requirements of the pipeline.

Our Contributions

This paper presents the *Container Profiler*, a tool that supports profiling the computational resources utilized by software within a Docker container. Our tool is simple, easy-to-use, and can record the resource utilization for any Dockerized computational job. Understanding fine-grained resource utilization of containerized computational tasks can help identify resource bottlenecks and inform the choice of optimal cloud deployment. The *Container Profiler* collects over sixty metrics to characterize the CPU, memory, disk, and network resource utilization at the VM, container, and process level. In addition, the *Container Profiler* supports time-series graphing enabling the visualization and monitoring of resource utilization of containerized tasks and pipelines.

We present a case study involving profiling the resource utilization of a multi-stage containerized bioinformatics pipeline that analyzes the unique molecular identifiers (UMI) of RNA sequencing data. In this study, we demonstrate how the *Container Profiler* performed time-series sampling at a one-second interval while a compute-bound bioinformatics pipeline simultaneously ran up to 85 distinct processes. Under load, our tool was able to profile the RNA-sequencing pipeline with full verbosity (all metrics) with 100% of the profiling samples obtained in under 100ms.

Related Work

Cloud computing has been used to process massive RNA sequencing (RNA-seq) datasets [10, 11]. These pipelines typically consist of multiple computational tasks, where not all tasks necessarily have the same resource requirements. Tatlow *et al.* studied the performance and cost profiles for processing large-scale RNA-seq data using pre-emptible virtual machines (VMs) on the Google Cloud Platform [10]. The authors collected resource utilization metrics to characterize user and system vCPU utilization, memory usage, disk activity, and network activity for the different computational stages of the RNA-seq pipeline. Tatlow *et al.* observed how resource utilization can vary dramatically across different processing tasks in the pipeline, while demonstrating that resource profiling can help to identify resource requirements of unique pipeline stages. Juve *et al.* developed a pair of tools called wfprof (pipeline profiling) to collect and summarize performance metrics for diverse scientific pipelines from multiple domains including bioinformatics [12]. Wfprof consists of two tools, ioprof to measure process I/O, and pprof that characterizes process runtime, memory usage, and CPU utilization. These tools accomplish profiling at the machine level primarily by analyzing process level resource utilization, and they do not focus on profiling containerized pipelines, nor do they collect container specific metrics.

Tyryshkina, Coraor, and Nekrutenko leveraged coarse grained resource utilization data from historical job runs collected over 5 years on the Galaxy platform to estimate the required CPU time and memory to improve task scheduling [13]. Galaxy, a scientific workflow, data integration, data analysis, persistence, and publishing platform was initially developed for genomics research and is now considered largely domain agnostic and is used for processing general bioinformatics pipelines. The authors identified the challenge of determining the appropriate amount of memory and processing resources for scheduling bioinformatics analyses at scale. The majority of metrics in the study consisted of metadata regarding job configurations. Assessing the utility of using fine grained operating system metrics as with the Container Profiler to profile resource utilization of genomics pipelines was not the focus. This effort considered many older jobs that ran on Galaxy where containers were not used thus they lacked container based metrics.

Outside bioinformatics, Weingartner et al. highlight the importance of profiling resource requirements of applications for deployment in the cloud to improve resource allocation and forecast performance [14]. Brendan Gregg described the USE method (Utilization, Saturation, and Errors) as a tool to diagnose performance bottlenecks [15]. Gregg's method involves

checking utilization of every resource involved in the system including CPUs, disks, memory, and more to identify saturation and errors. Lloyd *et al.* provided a virtual machine manager known as VM-scaler that integrated resource utilization profiling of software deployments to Infrastructure-as-a-Service (IaaS) cloud VMs [16]. VM-scaler focused on the management and profiling of cloud infrastructure used to host environmental modeling web services. This work was later extended by building resource utilization models to enable identifying the most cost effective cloud VM types to host environmental modeling web service workloads without sacrificing runtime or throughput [17]. This effort demonstrated a cost variance of 25% for hosting these workloads across different VM types on the Amazon Elastic Compute Cloud (EC2) while identifying potential for cost savings up to $25,000 for 10,000 hours of compute time.

To characterize resource requirements of containerized tasks and pipelines, a variety of commercial and open source tools exist. The vast majority of the available tools, however, require the setup and maintenance of a complete monitoring application including a time-series database and web application server. [18] These monitoring applications require dedicated infrastructure (i.e. servers and/or virtual machines) to run always-on daemons. Many of these tools are also oriented towards monitoring entire container clusters (e.g. Kubernetes). Access to such cluster-level monitoring tools is often restricted organizationally to system administrators and privileged users and not made freely available to any user. For container profiling, there are far fewer solutions that enable a user to easily profile the resource utilization of containerized tasks or pipelines on a local computer or personal cloud VM with minimal effort and expertise. The lack of lightweight easy-to-use developer tools that require no setup or maintenance of a permanent monitoring application and/or database server is what motivated the creation of the *Container Profiler*.

CMonitor as a related tool has been developed to support similar goals of lightweight container profiling without setup of a full monitoring application [19, 20]. CMonitor is installed and run on the host and is used to profile host metrics in addition to container metrics as the tool is not focused specifically on profiling a containerized task or pipeline. CMonitor, however, runs as an external tool which requires the user to posses detailed information about the host's operating system, runtime configuration, and Docker setup. Additionally CMonitor does not support container profiling of ARM-based Linux VMs or servers. These systems are of interest with the advent of low-cost compute-optimized VMs based on the Graviton series of ARM CPUs (e.g. c6g and c7g) on Amazon EC2 [21, 22, 23]. These VMs offer performance improvements and cost savings of interest for executing bioinformatics pipelines. CMonitor is installed as a package requiring several dependencies.

Container Profiler: Overview

The *Container Profiler* tool supports profiling resource utilization including CPU, memory, disk, and network metrics of containerized tasks. Resource utilization metrics are obtained across three levels: virtual machine (VM)/host, container, and process. Our implementation leverages facilities provided by the Linux operating system that is integral with Docker containers. Development and testing of the *Container Profiler* described in this paper was completed using Debian-based Ubuntu Linux.

The *Container Profiler* collects information from the Linux `/proc` and `/sys/fs/cgroup` file systems while a workload is running inside a container on the host machine. To support collecting metrics the *Container Profiler* is implemented using Python3 while leveraging psutil, a cross-platform library for retrieving information on running processes and system utilization [24]. It should be noted that psutil itself is not a profiling tool. Psutil assists with collecting host-level and process-level metrics from the system, but does not process metrics for time-series analysis or graphing. Psutil also does not output metrics in specific formats (e.g. JSON, CSV) or orchestrate time-series profiling. The host machine could be a physical computer such as a laptop or a virtual machine (VM) in the public cloud. The workload being profiled can be any job capable of running inside a Docker container. Figure 1 provides an overview of the various metrics collected by the *Container Profiler*.

Host-Level Metrics: Host/VM level resource utilization metrics are obtained from the Linux `/proc` virtual filesystem using psutil. The `/proc` filesystem is a virtual filesystem that consists of dynamically generated files produced on demand by the Linux operating system kernel providing an immense amount of data regarding the state of the system [25]. Files in the `/proc` filesystem are generated at access time from metadata maintained by Linux to describe current resource utilization, devices, and hardware configuration as managed by the Linux kernel. The *Container Profiler* queries the `/proc` filesystem directly and by using the psutil library at regular time intervals to obtain resource utilization metrics. Documentation regarding the Linux `/proc` filesystem is found on the `/proc` Linux manual pages [25] though other references provide more detailed descriptions of available metadata: [26, 27, 28, 29, 30, 31, 32, 33, 34, 35, 36]. User-mode and kernel-mode CPU utilization metrics can be obtained found in the `/proc/stat` file. Table 1 provides a subset of CPU, disk, and network utilization metrics profiled by the *Container Profiler* at the VM/host level.

Container-Level Metrics: Docker relies on the Linux `cgroup` and `namespace` features to facilitate the aggregation of a set of Linux processes together to form a container. `Cgroups` were originally added to the Linux operating system to provide system administrators with the ability to dynamically control hardware resources for a set of related Linux processes [37]. Linux control groups (`cgroups`) provide a kernel feature to both limit and monitor total resource utilization of containers. Docker leverages `cgroups` for resource management to restrict hardware access to the underlying host machine to facilitate sharing when multiple containers share the host. Linux subsystems such as CPU and memory are attached to a `cgroup` enabling the ability to control resources of the `cgroup`. Resource utilization of `cgroup` processes is aggregated for reporting purposes under the `/sys/fs/cgroup` virtual filesystem and we leverage this filesystem to obtain container-level metrics in the *Container Profiler*. `Cgroup` files provide aggregated resource utilization statistics describing all of the processes inside a container. Container-level metrics are not available from psutil. As a profiling example, a container's CPU utilization statistics can be obtained from `/sys/fs/cgroups/cpuacct/cpuacct.stat`. Table 2 describes a subset of the CPU, disk, and network utilization metrics profiled at the container level by the *Container Profiler*.

Process-Level Metrics: The *Container Profiler* also supports profiling the resource utilization for each process running inside a container. The *Container Profiler* leverages support from the psutil library to capture process level metrics from Linux. Table 3 describes a subset of the process-level metrics collected by the *Container Profiler* to profile resource utilization of container processes.

Resource utilization data collected at the VM/host, container, and process level allows characterization of resource use with increasingly greater isolation. Host-level resource metrics for example, do not isolate background processes. This could lead to variance in measurements as background processes on the host machine outside the container may be randomly present. Profiling at the container level allows fine-

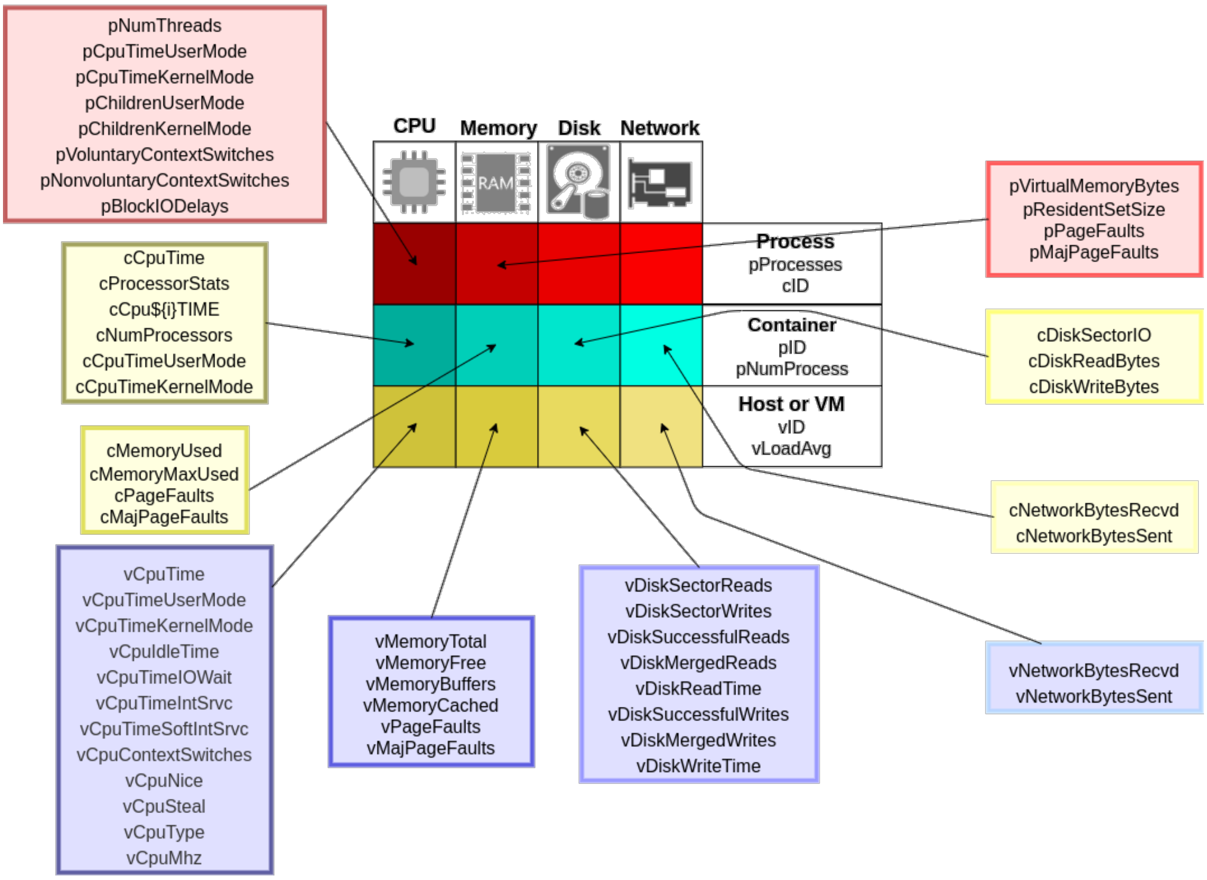

Figure 1. Overview summarizing resource utilization metrics (61 total) collected by the *Container Profiler* across three levels (i.e. host/VM, container, and process level) and four categories (i.e. CPU, memory, network, and disk). Process level metrics are depicted by red and prefaced with lower case "p", container level metrics by yellow and prefaced with lower case "c", and host/VM level metrics by blue and prefaced with lower case "v".

Table 1. Selected CPU, disk, and network utilization metrics profiled at the VM/host level.

Metric	Description	Source
vCpuTimeUserMode	Time the CPU spent executing in user mode	/proc/stat
vCpuTimeKernelMode	Time the CPU spent executing in kernel mode	/proc/stat
vCpuIdleTime	Time the CPU was idle	/proc/stat
vCpuTimeIOWait	Time the CPU waits for I/O to complete	/proc/stat
vCpuContextSwitches	The total number of context switches across all CPU cores	/proc/stat
vDiskSectorReads	Number of sector reads	/proc/diskstats
vDiskSectorWrites	Number of sectors writes	/proc/diskstats
vDiskReadTime	Time spent reading	/proc/diskstats
vDiskWriteTime	Time spent writing	/proc/diskstats
vNetworkBytesRecvd	Network Bytes received	/proc/net/dev
vNetworkBytesSent	Network Bytes written	/proc/net/dev

grained resource profiling of ONLY the resources used by the containerized task or pipeline. Finally, profiling at the process level allows very fine-grained profiling so that resource bottlenecks can be attributed to the specific activities or tasks. The ability of the *Container Profiler* to characterize resource utilization at multiple levels enables high observability of the resource requirements of computational tasks. This observability can be crucial to improving job deployments to cloud platforms to alleviate performance bottlenecks and optimize performance and cost.

Results

We demonstrate the Container Profiler using unique molecular identifier (UMI) RNA sequencing data generated by the LINCS Drug Toxicity Signature (DToxS) Generation Center at Icahn School of Medicine at Mount Sinai in New York [38]. The scripts and supporting files for the analytical pipeline to analyse this originated from the Broad Institute [39]. In addition to downloading the datasets, there are 3 other stages. The first stage is a demultiplexing or split step that sorts the reads using a sequence barcode to identify the originating sample. The second stage aligns the reads to a human reference sequence to identify the gene that produced the transcript. The final stage is

Table 2. Selected CPU, disk, and network utilization metrics profiled at the container level.

Metric	Description	Source
cCpuTimeUserMode	CPU time consumed by tasks in user mode	/sys/fs/cgroup/cpuacct/cpuacct.stat
cCpuTimeKernelMode	CPU time consumed by tasks in kernel mode	/sys/fs/cgroup/cpuacct/cpuacct.stat
cDiskSectorIO	Number of sectors transferred to or from specific devices	/sys/fs/cgroup/blkio/blkio.sectors
cDiskReadBytes	Number of bytes transferred from specific devices	/sys/fs/cgroup/blkio/blkio.throttle.io_service_bytes
cDiskWriteBytes	Number of bytes transferred to specific devices	/sys/fs/cgroup/blkio/blkio.throttle.io_service_bytes
cNetworkBytesRecvd	The number of bytes each interface has received	/proc/net/dev
cNetworkBytesSent	The number of bytes each interface has sent	/proc/net/dev

Table 3. List of important metrics for profiling process resource utilization.

Metric	Description	Source
pCpuTimeUserMode	Amount of time that this process has been scheduled in user mode	/proc/[pid]/stat
pCpuTimeKernelMode	Amount of time that this process has been scheduled in kernel mode	/proc/[pid]/stat
pVoluntaryContextSwitches	Number of voluntary context switches	/proc/[pid]/status
pNonvoluntaryContextSwitches	Number of involuntary context switches	/proc/[pid]/status
pBlockIODelays	Aggregated block I/O delays	/proc/[pid]/stat
pResidentSetSize	Number of pages the process has in real memory	/proc/[pid]/stat

the "merge" step which counts all the aligned reads to identify the number of transcripts produced by each gene. The unique molecular identifier (UMI) sequence is used to filter out reads that arise from duplication during the sample preparation process. In the original pipeline, only the most CPU intensive part of the pipeline, the alignment step, was optimized and executed in parallel. We further optimized the split and align steps in the original pipeline [39] to decrease the running time from 29 to 3.5 hours in our previous work [40]. We also encapsulated each step in the pipeline in separate Docker containers to facilitate deployment and ensure reproducibility.

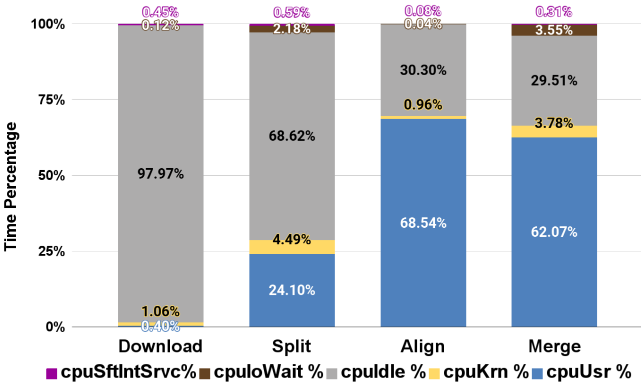

Figure 2. CPU utilization graph for the four stages (e.g. download, split, align, and merge) of the UMI RNA-seq pipeline. This graph depicts the percentage of CPU utilization in each CPU mode. CpuUsr (shown in green) captures time the pipeline spent executing its source code. CpuKrn (shown in yellow) captures time when the processor executed code in the Linux kernel. Typically the kernel is invoked to support disk and network I/O which are considered privileged operations. CpuIdle (shown in blue) is unused time across the 8 available CPU cores throughout each stage. CpuIdle time is common when waiting for disk or network I/O to complete. High CpuIdle time during computational stages indicates potential for performance optimization with better parallelization of code. CpuIOWait (shown in maroon) depicts CPU time where the pipeline was waiting for I/O (disk or network) to complete. cpuSftIntSrvc (shown in magneta) is time spent handling soft interrupts. Soft interrupts commonly occur with network I/O.

To profile resource utilization, we deployed our UMI RNA-sequencing pipeline alongside the *Container Profiler* on an IBM Cloud bx2d-metal-96x384 virtual machine with dual Intel Platinum 8260 CPUs at 2.4 GHz, with 96 virtual CPU cores, 384GB of memory, and a 960 GB SATA M.2 mirrored SSD as the local boot disk. We leveraged the UMI RNA-sequencing pipeline as our case study as each stage of the RNA-seq pipeline exhibits different resource utilization characteristics. Specifically, the dataset download stage is limited by the network capacity. The split stage writes many files and is limited by the speed of disk writes. The alignment stage is performed by multiple CPU-intensive processes and performance is primarily limited by the CPU. However, it is possible that available memory capacity will limit the performance in some circumstances. The final merge stage involves reading many files in parallel, consuming both memory and CPU resources depending on the number of threads used.

Container Profiler can inform pipeline optimization

Figure 2 summarizes the CPU utilization characteristics of different stages of the UMI RNA-seq pipeline. The CPU usage profile is consistent with our expectations. The execution of the align and merge steps are expected to be bound by CPU resources and they indeed spent the majority of the time executing source code. Download is limited by the network bandwidth and the split stage by disk I/O. Hence the cpuIdle time is highest in these stages.

Despite the fact that the align stage is expected to be limited by the CPU resources, there is significant CPU-idle time during that stage. This suggests the presence of a bottleneck that may be the target for further optimization. We collected CPU, memory, network, and disk utilization metrics at both the container and VM/host levels for the RNA sequencing analytical pipeline. These are visualized in Figure 3. Note that the x-axis depicting time in this figure encompasses the entire pipeline incorporating all stages: download, split, align, and merge. Overall our profiling results depict resource utilization patterns that we expected. The download stage consumes network resources. The split stage is the most disk intensive step. The alignment and merge stages consume the most CPU resources. Our profiling

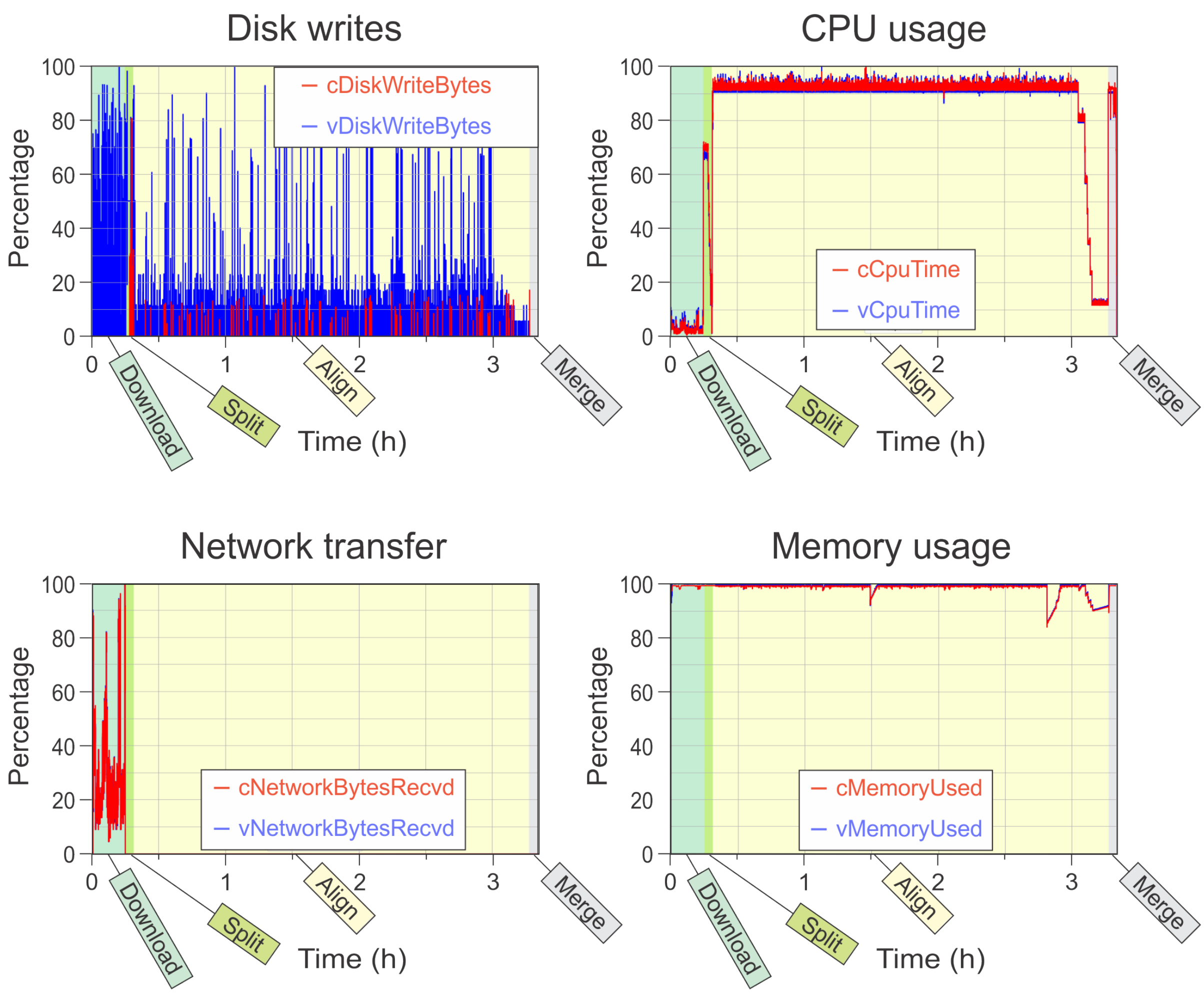

Figure 3. Output graphs comparing Container and VM (host) level metrics over time for a multi-stage RNA sequencing data pipeline. Four output graphs are shown: disk writes (top left), CPU usage (top right), network usage (bottom left) and memory usage (bottom right). In each graph, the container level metrics are shown in red and the VM (host) level metrics are shown in blue. For disk usage and memory usage, the native host metric was transformed to have the same units as the container metric. All metrics have been transformed to the same units and scaled as a percentage of the maximum observed value. The four stages of the pipeline include downloading the data (download), splitting and demultiplexing the reads (split), aligning the reads to the reference (align), and assembling the counts while removing duplicate reads (merge). We observe that the container and VM-level metrics mostly overlap in the stages. However, there are differences when there are background processes, most notably when there is considerable disk usage. The alignment stage is also notable in that we can see that the CPU usage declines near the end, probably indicating that the pipeline is waiting on some slower threads (i.e. stragglers) to finish before it can proceed, indicating this stage might be improved with better load balancing, or with smaller workloads for the threads. This is an example of how the *Container Profiler* can be used to identify portions of the pipeline that can be optimized.

data also points to areas where resource consumption may be a problem. For example, memory usage is high for all the stages. This may be due to greedy allocation by the executables, or it may indicate that allocating more memory could benefit the pipeline. Most interesting, is CPU utilization during the alignment stage. Just before the 3 hour mark, we see a series of drops over the next 30 minutes, creating a ladder of 8 steps. The alignment stage uses up to 8 vCPUs to align different files of reads simultaneously. Near the end of the alignment stage, most of the files will have been processed and there will be more available vCPUs than unprocessed files. As a result, the CPU utilization drops as vCPUs lie idle waiting for the final files to be processed. However, this under-utilization of resources lasts for 30 minutes indicating that the final files are rather large. This presents an opportunity to improve pipeline performance by splitting the processing into smaller files (which is an option in the split software), or by processing the largest files first. We would not have known about these potential optimizations without fine-grained profiling results from the *Container Profiler*.

Container-level metrics can provide useful additional information

A key feature of the *Container Profiler* is the ability to capture container-level metrics to describe resource utilization of only the containerized task(s). We expect these metrics to be similar, and that they could differ given that the VM/host level metrics also encompass resources being used by processes running on the host external to the container and pipeline. Since we only ran our pipeline on an dedicated test VM, the container metrics should be very similar to the VM/host metrics, which was in fact the case from our observations. However, one can see differences between the disk utilization metrics during the split and alignment stages where there are a large number of disk writes to the host file system. Docker manages these disk writes by providing the container with an internal mount point which is eventually written to a host file. The caching and management of this data is external to the container and is not captured by the container metrics, but is captured by the host metric. In addition, during the alignment stage, intermediate results from the aligner are continuously piped to another process which then re-formats the intermediate output

and writes the final output to a file on the host system. Multiple threads are used, more than the available number of cores resulting in frequent context switches. The pipe management and context-switching are also handled by the operating system and are captured by the host metric and not the container metrics. The separation of container and OS based consumption can be useful for example, when trying to assess effects due to resource contention that may occur when multiple jobs are run on the same physical host, which often happens on public clouds where the assignment of instances to hosts is controlled by the vendor.

Container Profiler can sample container and host metrics with sub-second resolution

For the *Container Profiler* to be useful, the collection of profiling metrics must have sufficiently low overhead to enable rapid sampling of resource utilization to collect many samples for time series analysis. The time required to collect the metrics limits the granularity of the profile. To achieve 1 second sampling for time series analysis requires the ability to repeatedly sample resource utilization every 1 second (1000 ms). However, profiling time is not constant, and depends on the state of resources being utilized by the containerized pipeline and the host. The variability of profiling time is shown in the histogram in Figure 4. When profiling our RNA-sequencing pipeline, VM-level and container-level profiling had a bi-modal distribution, while process-level sampling had a tri-modal distribution. The slowest profiling was observed during the stressful compute-bound alignment stage of the pipeline. For all levels of profiling verbosity, the *Container Profiler* was able to profile resource utilization in less than 100ms. The longest profiling time and highest variation was for process-level profiling as metrics are collected for each process in the pipeline. The number of processes can vary throughout the execution of complex parallel pipelines, as was the case for the align stage of our RNA-sequencing pipeline. Our RNA-sequencing pipeline featured a maximum of 85 concurrent processes during the align stage. These processes ran for approximately 39% of the duration of the align stage. The time required to capture host and container level metrics was less variable as the number of metrics collected is fixed. As shown in Figure 4, 90% of the time, the container and host level metrics were collected in less than 63 milliseconds and always under 75 milliseconds. The process metrics do take longer to collect but still less than 100 milliseconds in the worst case. Profiling at the process-level involves collecting all metrics every second. For profiling our UMI RNA-sequencing pipeline use case which required 2.5 hours to execute with one-second sampling and full profiling verbosity (process-level metrics), 9,000 JSON files were collected which required 296 MB of storage space.

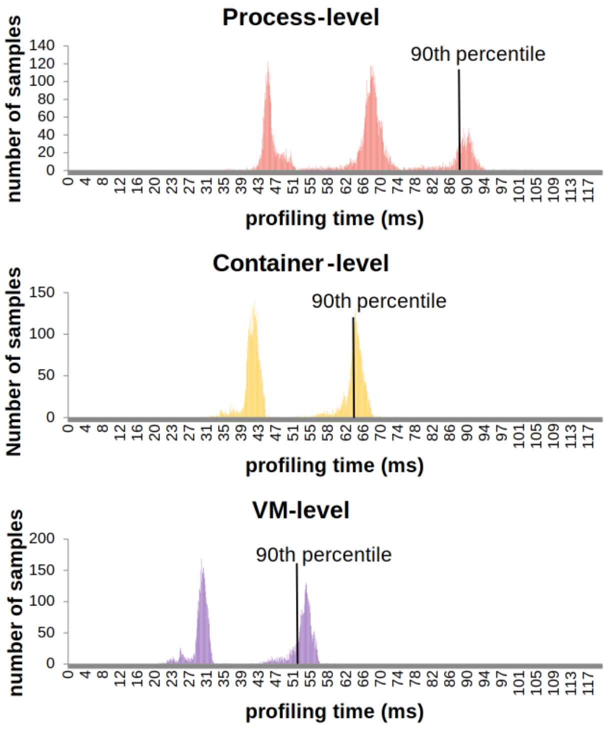

Figure 4. Distribution plot (log-scale) of time required to collect profiling data. We profiled resource utilization of the RNA-sequencing pipeline on an IBM Cloud bx2d-metal-96x384 virtual machine with dual Intel Platinum 8260 CPUs at 2.4 GHz, with 96 virtual CPU cores, 384GB of memory, and a 960 GB SATA M.2 mirrored SSD as the local boot disk). We executed the complete RNA-seq pipeline four times to profile 1) only VM/host metrics, 2) VM/host and container metrics, 3) *ALL* metrics, and no metrics by running the pipeline in the absence of the profiler. Plots depict time to collect resource utilization samples at one-second intervals with the Container Profiler while running the entire RNA-seq pipeline. Time to collect 9000 samples of each type (Process-level, Container-level, and VM-level) is shown. 99.95% of process-level samples were collected under 100 milliseconds, while all container-level samples were collected under 74 milliseconds, and all VM-level samples were collected at or under 60 milliseconds. The figure shows the process-level, container-level, and VM-level profiling time distribution over 120 milliseconds on the x-axis. The 90th percentiles for sample collection are shown.

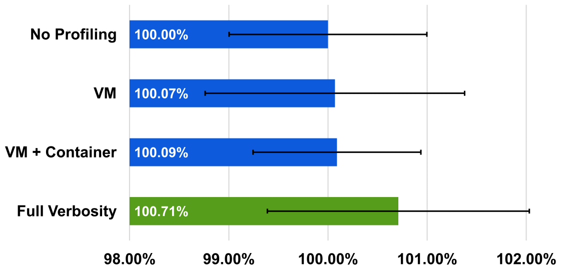

Figure 5. This figure depicts the profiling overhead of the *Container Profiler* and the resulting percentage increase in the total runtime of the entire RNA-seq pipeline. The increases in run time are very modest: Host/VM only (0.07%), Host/VM + Container (0.09%), and Host/VM + Container + Process (0.71%). Error bars depict one standard deviation from the average. Standard deviation of pipeline runtime for 5 runs of the RNA-seq pipeline on the IBM bx2d-16x64 Virtual Machine with no profiling was (1.38%), approximately 194% greater than the worst case overhead of the Container Profiler when profiling with full verbosity (i.e. collecting all metrics).

Container Profiler has lower overhead than the variation in pipeline execution time on public clouds

A design objective for the *Container Profiler* is to not significantly impact the performance of the pipeline being profiled. Failing to realize this objective may result in the overhead from resource profiling impacting the collected metrics. While some overhead is unavoidable, ideally it should be lower than the inherent variations of pipeline execution time on the public cloud.

To measure the performance impact of resource utilization profiling when running the RNA-seq pipeline, we initially attempted to assess the overhead using Amazon Elastic Compute Cloud (EC2) cloud VMs. However, we discovered that the runtime of the RNA-seq pipeline varied by more than 5% on Amazon EC2, which was more than 5x greater than the overhead of the *Container Profiler*. This degree of performance variance made it difficult to evaluate the performance overhead of the

Container Profiler since we could not easily distinguish between pipeline performance variance and profiling overhead on EC2. We then measured the performance overhead of the *Container Profiler* by profiling the pipeline using the IBM cloud bx2d-metal-96x384 server which had performance variance around 1%. Figure 5 depicts the overhead from one-second resource utilization sampling by the *Container Profiler* for the RNA-seq pipeline on the IBM metal server. IBM metal servers are private and not shared with multiple users. Running on this isolated server greatly reduced the performance variance of running RNA-seq. We measured worst case overhead for the *Container Profiler* to be 0.71%, which equates to about 3.4 minutes for an 8-hour pipeline with full verbosity metrics collection (VM + container + process). Overhead is reduced to as little as .07% overhead, or about 20 seconds for an 8-hour pipeline when only collecting VM-level metrics. Adding container-level, and especially process-level metrics slightly increased the runtime of the RNA-seq pipeline for collecting resource utilization data. We believe that this profiling overhead is within an acceptable level and note that even at maximum profiling verbosity, it is substantially less than the observed performance variance for running our RNA-seq pipeline on a public cloud VM. Users can reflect on our reported overhead times to make informed decisions when planning to profile their own pipelines.

Methods

Implementation Details

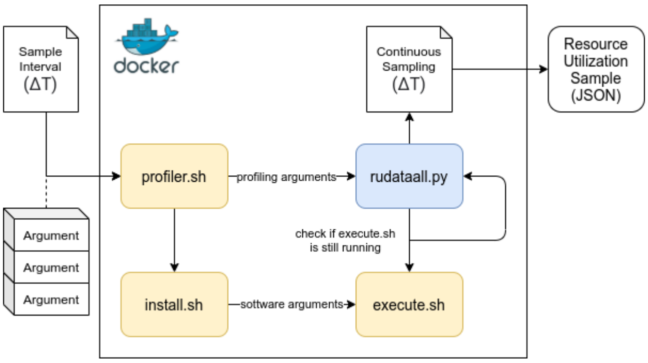

Figure 6. Profiling scripts used in the implementation of Container Profiler. All scripts are deployed inside the container alongside the software being profiled. The software to be profiled can be installed using an install script, or the Container Profiler can be installed on top of the original container image. The user provides a sampling interval and profiling arguments to initialize profiling.

The *Container Profiler* is implemented as a collection of Bash and Python scripts. Figure 6 provides an overview. There are three basic use cases for building a docker image for the Container Profiler. The first use case allows users to profile an existing Docker container by providing a Dockerfile which specifies their own setup and software installation inside the container. This is the simplest approach to profiling when the user has a working Dockerfile. The other two use cases support users who do not know how to write Docker files but are familiar with writing Bash scripts. The second use case gives users the ability to install all software inside the Docker image when the software installation becomes too complicated to put in the Dockerfile. In other words, it puts the required installation commands into a script that will be executed by the Dockerfile. For the third use case, the user provides their own executable bash script as the entry point in the Docker container. This use case can help the user simplify a set of commands they have to profile. In this case, the user just puts a set of commands into an executable script file and runs it as the entrypoint of the container. When the *Container Profiler* is executed inside a Docker container, it snapshots the resource utilization for the host (i.e. VM), container, and all processes running inside the container producing output statistics to a `.json` file. A sampling interval (e.g. once per second) is specified to configure how often resource utilization data is collected to support time series analysis of containerized applications and pipelines. Time series data can be used to train mathematical models to predict the runtime or resource requirements of applications and pipelines. Time series data can be visualized by using matplotlib Python graphing scripts that are included with the *Container Profiler*.

To improve the periodicity of time series sampling, we continuously subtract the most recent observed run time of the *Container Profiler* for sample collection from the configured sampling interval (e.g. 1 second) in `rudataall.py`. This approach notably improved the periodicity of sampling when the container was under load improving our ability to obtain samples at evenly spaced intervals. To enable addressing any potential drift of sample collection times, we capture timestamps for when each resource utilization metric is sampled in the output JSON. These timer ticks enable precise calculation of the time that transpires between resource utilization samples for each metric. This allows the rate of consumption of system resources (e.g. CPU, memory, disk/network I/O) to be precisely determined throughout the pipeline's execution. The *Container Profiler* consists of the profiling script and two supporting scripts (for installation and pipeline execution) depicted in Figure 6: `profiler.sh`, `install.sh`, and `execute.sh`.

The `profiler.sh` script is the primary script that generates profiling information in JSON format describing resource utilization of the containerized task. The `profiler.sh` script requires the user to provide a command or a set of commands along with arguments to start the profiling. This script internally invokes another Python script `rudataall.py`.

The `rudataall.py` script collects the resource utilization data. Specifically, this script takes a snapshot of the resource utilization metrics and records output to a JSON file using the time of the sample as a unique filename. The script accepts parameters `-v`, `-c`, and `-p` to inform the tool what type of data to collect: VM, container, and/or processlevel metrics respectively. The default behavior when running this script without any parameters is to collect all metrics.

The `profiler.sh` script only works if the workflow/software is already installed in the containerized environment. This means that we cannot profile workflows/software that has not been containerized. The *Container Profiler* provides an option that enables users to install software in a container using the `install.sh` script. Users provide a set of commands in the `install.sh` script to install their dependencies and software they wish to profile. Once installed, the user can run the `profiler.sh` script against the newly installed software. To profile resource utilization of a bash script, users can specify a series of commands using the optional `execute.sh` script to configure profiling.

Some users may be more familiar with editing Dockerfiles instead of bash scripts. We provide support for users to provide their own Dockerfile to build a custom container to be profiled.

Technical details using our scripts

To use the *Container Profiler* scripts with any container, a Linux based Docker container that encapsulates a script or job to run

Table 4. Container Profiler with four different modes

Mode	Description
profile	profile resource utilization
delta	calculate the different between two profiling samples
csv	convert a set of JSON resource utilization files into a single CSV file
graph	generate profiling graph(s) from a CSV file

inside is required. To configure the *Container Profiler* tool to profile the container, users can optionally provide an executable script inside the *Container Profiler* which is specified during the `build.sh` script. In the executable script, the user launches the container's job or task to be profiled.

The `profiler.sh` script has four different modes: `profile`, `delta`, `csv`, and `graph`:

For the `profile` mode, there are two required parameters: the output directory specifies the location of generated profiling files in JSON format, and the time interval specifies a time series sampling interval in milliseconds. The profiler generates a JSON file at the beginning and the end of the process if the sampling interval is set to zero. Otherwise, the profiler generates a JSON file at each sampling interval. The Container Profile also collects static metrics which typically describe hardware characteristics. The profiler first checks if a static information file exists (static.json). If missing, the profiler captures static parameters and writes out the static information file at the start of profiling. By default 11 static metrics are captured. They include: the host's kernel info, the host's cpu type, CPU Level 1 instruction cache size, CPU Level 1 data cache size, CPU Level 2 cache size, CPU Level 3 cache size, host boot time, host VM ID, the number of CPU cores available to the container, and the container ID.

For the `delta` mode, there are two required parameters: the input directory which contains the original raw JSON files, and the output directory where the delta JSON files will be written. The `delta| mode` also provides an option to allow users to specify the modification operator for performing the delta. The default delta operator calculates the difference between two samples (i.e. final minus initial value). The typical use case is to calculate the delta of the resource utilization between the first and last sample to capture the full resource utilization of a task or pipeline. Other operators include max, min, and average to determine the max, min, and average values of metrics from a set of JSON files.

For the `csv` mode, there are two required parameters: the input directory that contains processed JSON files in delta format, and the name of an output CSV file where all resource utilization data from the processed JSON files will be aggregated to.

For the `graph` mode, there are two required parameters: the input CSV file capturing all resource utilization data from processed JSON files, and the output directory for writing graph files. In addition, there are a few other options such as one to specify whether to plot the curves together or using separate graph files.

Visualization

The *Container Profiler* in the `graph` mode also provides an option to specify the creation of time-series graphs. The graphing configuration file supports multiple settings to specify how to generate graph(s). Each graph configuration file should start with a line that includes the components: the `###` followed by the title and the y-coordinate label. This is followed by line(s) that describe the metric(s) that users want to output in a single graph (one metric per line). As a starting point, a default graph configuration file `graph.cfg` is provided in the `cfg` directory.

Data Availability

An archival copy of the code and supporting data including processed data, raw data, and Jupyter notebook files are available via the GigaScience repository, GigaDB [41]. The read counts datasets from [42] used in this study are deposited on GEO: GSE98432.

Availability of source code and requirements

- Project name: Container Profiler
- Project home page: https://github.com/wlloyduw/ContainerProfiler
- Contents available for download: Docker Images, Dockerfiles, installation scripts, and execution scripts.
- Operating system(s): Linux, Mac OS X.
- Programming language(s): Python, Bash
- License: MIT License
- SciCrunch.org RRID: RRID:SCR_023770
- bio.tools ID: https://bio.tools/containerprofiler

Declarations

List of abbreviations

AWS: Amazon Web Services; EC2: Elastic Compute Cloud; VM: virtual machine; CPU: central processing unit; IaaS: Infrastructure-as-a-Service; RNAseq: RNA sequencing; LINCS: Library of Integrated Network-Based Cellular Signatures; DToxS: Drug Toxicity Signature; RNA: ribonucleic acid; cgroup: container control group.

Consent for publication

Not applicable.

Competing Interests

LHH and KYY have equity interest in Biodepot LLC, which receives compensation from NCI SBIR contract numbers 75N91020C00009 and 75N91021C00022. The terms of this arrangement have been reviewed and approved by the University of Washington in accordance with its policies governing outside work and financial conflicts of interest in research.

Author's Contributions

VH, LHH, HD, RS, and DP contributed to the development of the Container Profiler. LHH implemented Docker containers for RNA-seq pipelines. VH, RS, NA, and DP conducted performance testing and empirical experiments. KYY, RS, WL, VH, and LHH

drafted the manuscript. WL, KYY, and LHH designed the case study. WL provided cloud computing expertise. WL and KYY coordinated the benchmarking experiments. All authors edited the manuscript.

Acknowledgements

LHH, HD, RS, WL, and KYY are supported by the National Institutes of Health (NIH) grant R01GM126019. DP is supported by the NIH Diversity Supplement R01GM126019-02S2. LHH and KYY are also supported by NIH grants U24HG012674 and R03AI159286. WL is also supported by NSF grant OAC-1849970. We acknowledge support from the AWS Cloud Credits for Research and IBM Cloud Credits (awarded to LHH, WL, and KYY).

Supplementary Information

References

1. O'Connor BD, Yuen D, Chung V, Duncan AG, Liu XK, Patricia J, et al. The Dockstore: enabling modular, community-focused sharing of Docker-based genomics tools and workflows. F1000Research 2017;6.
2. da Veiga Leprevost F, Grüning BA, Alves Aflitos S, Röst HL, Uszkoreit J, Barsnes H, et al. BioContainers: an open-source and community-driven framework for software standardization. Bioinformatics 2017;33(16):2580–2582.
3. Dai L, Gao X, Guo Y, Xiao J, Zhang Z. Bioinformatics clouds for big data manipulation. Biology direct 2012;7(1):43.
4. Schadt EE, Linderman MD, Sorenson J, Lee L, Nolan GP. Computational solutions to large-scale data management and analysis. Nature reviews genetics 2010;11(9):647.
5. Schadt EE, Linderman MD, Sorenson J, Lee L, Nolan GP. Cloud and heterogeneous computing solutions exist today for the emerging big data problems in biology. Nature Reviews Genetics 2011;12(3):224.
6. Lau JW, Lehnert E, Sethi A, Malhotra R, Kaushik G, Onder Z, et al. The Cancer Genomics Cloud: collaborative, reproducible, and democratized—a new paradigm in large-scale computational research. Cancer research 2017;77(21):e3–e6.
7. Reynolds SM, Miller M, Lee P, Leinonen K, Paquette SM, Rodebaugh Z, et al. The ISB Cancer Genomics Cloud: a flexible cloud-based platform for cancer genomics research. Cancer research 2017;77(21):e7–e10.
8. Afgan E, Baker D, Coraor N, Goto H, Paul IM, Makova KD, et al. Harnessing cloud computing with Galaxy Cloud. Nature biotechnology 2011;29(11):972.
9. Birger C, Hanna M, Salinas E, Neff J, Saksena G, Livitz D, et al. FireCloud, a scalable cloud-based platform for collaborative genome analysis: Strategies for reducing and controlling costs. bioRxiv 2017;p. 209494.
10. Tatlow P, Piccolo SR. A cloud-based workflow to quantify transcript-expression levels in public cancer compendia. Scientific reports 2016;6:39259.
11. Lachmann A, Torre D, Keenan AB, Jagodnik KM, Lee HJ, Wang L, et al. Massive mining of publicly available RNA-seq data from human and mouse. Nature communications 2018;9(1):1366.
12. Juve G, Chervenak A, Deelman E, Bharathi S, Mehta G, Vahi K. Characterizing and profiling scientific workflows. Future Generation Computer Systems 2013;29(3):682–692.
13. Tyryshkina A, Coraor N, Nekrutenko A. Predicting runtimes of bioinformatics tools based on historical data: five years of Galaxy usage. Bioinformatics 2019;35(18):3453–3460.
14. Weingärtner R, Bräscher GB, Westphall CB. Cloud resource management: A survey on forecasting and profiling models. Journal of Network and Computer Applications 2015;47:99–106.
15. Gregg B. Thinking methodically about performance. Communications of the ACM 2013;56(2):45–51.
16. Lloyd W, David O, Arabi M, Ascough I, JC G, TR C, et al. The virtual machine (VM) scaler: an infrastructure manager supporting environmental modeling on IaaS clouds. In: Environmental Modeling International Conference Proceedings; 2014. .
17. Lloyd WJ, Pallickara S, David O, Arabi M, Wible T, Ditty J, et al. Demystifying the clouds: Harnessing resource utilization models for cost effective infrastructure alternatives. IEEE Transactions on Cloud Computing 2017;5(4):667–680.
18. 12 Best Docker Container Monitoring Tools [2022 Comparison] - Sematext;. (Accessed 02/2023). https://sematext.com/blog/docker-container-monitoring/.
19. f18m/cmonitor: A Docker/LXC/Kubernetes, database-free, lightweight container performance monitoring solution, perfect for ephemeral containers (e.g. containers used for DevOps automatic testing);. (Accessed 02/2023). https://github.com/f18m/cmonitor.
20. Ji S, Ye K, Xu CZ. Cmonitor: A monitoring and alarming platform for container-based clouds. In: International Conference on Cloud Computing Springer; 2019. p. 324–339.
21. Mathá R, Kimovski D, Zabrovskiy A, Timmerer C, Prodan R. Where to Encode: A Performance Analysis of x86 and Arm-based Amazon EC2 Instances. In: 2021 IEEE 17th International Conference on eScience (eScience) IEEE; 2021. p. 118–127.
22. Lambion D, Schmitz R, Cordingly R, Heydari N, Lloyd W. Characterizing X86 and ARM Serverless Performance Variation: A Natural Language Processing Case Study. In: Companion of the 2022 ACM/SPEC International Conference on Performance Engineering ICPE '22, New York, NY, USA: Association for Computing Machinery; 2022. p. 69–75. https://doi.org/10.1145/3491204.3543506.
23. Jiang Q, Lee YC, Zomaya AY. The power of ARM64 in public clouds. In: 2020 20th IEEE/ACM International Symposium on Cluster, Cloud and Internet Computing (CCGRID) IEEE; 2020. p. 459–468.
24. Rodola G, psutil - PyPI;. (Accessed 02/2023). https://pypi.org/project/psutil/.
25. proc(5) - Linux manual page - process information pseudo-filesystem;. (Accessed 02/2023). http://man7.org/linux/man-pages/man5/proc.5.html.
26. Linux Howtos: System -> /proc/stat explained;. (Accessed 02/2023). http://www.linuxhowtos.org/System/procstat.htm.
27. KB941772: Gathering CPU Utilization from /proc/stat;. (Accessed 02/2023). https://www.idnt.net/en-US/kb/941772.
28. /proc/cpuinfo (E.2.3.), Red Hat Enterprise Linux 6, Red Hat Customer Portal;. (Accessed 04/13/2020). https://access.redhat.com/documentation/en-us/red_hat_enterprise_linux/6/html/deployment_guide/s2-proc-cpuinfo.
29. /procfs/diskstats;. (Accessed 02/2023). https://www.kernel.org/doc/Documentation/ABI/testing/procfs-diskstats.
30. /proc/meminfo (E.2.18.), Red Hat Enterprise Linux 6, Red Hat Customer Portal;. (Accessed 02/2023). https://access.redhat.com/documentation/en-us/red_hat_enterprise_linux/6/html/deployment_guide/s2-proc-meminfo.
31. /proc/net/ (E.3.7.), Red Hat Enterprise Linux 6, Red Hat Customer Portal;. (Accessed 02/2023). https://access.redhat.com/documentation/en-us/red_hat_enterprise_

linux/6/html/deployment_guide/s2-proc-dir-net.
32. /proc/loadavg (E.2.15.), Red Hat Enterprise Linux 6, Red Hat Customer Portal;. (Accessed 02/2023). https://access.redhat.com/documentation/en-us/red_hat_enterprise_linux/6/html/deployment_guide/s2-proc-loadavg.
33. cgroup/cpuacct (3.3.), Red Hat Enterprise Linux 6, Red Hat Customer Portal;. (Accessed 02/2023). https://access.redhat.com/documentation/en-us/red_hat_enterprise_linux/6/html/resource_management_guide/sec-cpuacct.
34. Chapter 3. Subsystems and Tunable Parameters, Red Hat Enterprise Linux 6, Red Hat Customer Portal;. (Accessed 02/2023). https://access.redhat.com/documentation/en-us/red_hat_enterprise_linux/6/html/resource_management_guide/ch-subsystems_and_tunable_parameters.
35. /cgroup/memory (3.7.), Red Hat Enterprise Linux 6, Red Hat Customer Portal;. (Accessed 02/2023). https://access.redhat.com/documentation/en-us/red_hat_enterprise_linux/6/html/resource_management_guide/sec-memory.
36. /proc/net/ (E.3.7.), Red Hat Enterprise Linux 6, Red Hat Customer Portal;. (Accessed 02/2023). https://access.redhat.com/documentation/en-us/red_hat_enterprise_linux/6/html/deployment_guide/s2-proc-dir-net.
37. Linux Programmer's Manual;. (Accessed 02/2023). http://man7.org/linux/man-pages/man7/cgroups.7.html.
38. Xiong Y, Soumillon M, Wu J, Hansen J, Hu B, van Hasselt JGC, et al. A Comparison of mRNA Sequencing with Random Primed and 3′-Directed Libraries. Scientific Reports 2017;7(1):14626. https://doi.org/10.1038/s41598-017-14892-x.
39. Soumillon M, Cacchiarelli D, Semrau S, van Oudenaarden A, Mikkelsen TS. Characterization of directed differentiation by high-throughput single-cell RNA-Seq. BioRxiv 2014;p. 003236.
40. Hung LH, Lloyd W, Agumbe Sridhar R, Athmalingam Ravishankar SD, Xiong Y, Sobie E, et al. Holistic optimization of an RNA-seq workflow for multi-threaded environments. Bioinformatics 2019;35(20):4173–4175.
41. Hoang V, Hung LH, Perez D, Deng H, Schooley R, Arumilli N, et al. Supporting data for "Container Profiler: Profiling Resource Utilization of Containerized Big Data Pipelines". GigaScience Database;http://dx.doi.org/10.5524/102424.
42. Xiong Y, Soumillon M, Wu J, Hansen J, Hu B, Van Hasselt JG, et al. A comparison of mRNA sequencing with random primed and 3-directed libraries. Scientific reports 2017;7(1):14626.
