# Supplementary material for: Container Profiler: Profiling resource utilization of containerized big data pipelines
Source: Gigascience. 2023 Aug 25;12:giad069. doi: 10.1093/gigascience/giad069 (PMC10452954; doi:10.1093/gigascience/giad069)
Supplement: giad069_Response_to_Reviewer_Comments_Original_Submission [file giad069_response_to_reviewer_comments_original_submission.pdf]

Author's Response To Reviewer Comments

Close

All of the requested changes from Reviewer #1 have involved updates to the project's GitHub repository. These have been made.
The reviewers did not identify specific changes to the manuscript.
The authors have worked with the GigaScience editor to meet a number of other requirements including submitting data to GigaDB, providing funding information, ORCIDs, and registering the tool to obtain an RRID and bio.tools ID.
Please let us know of any further updates or modifications or additional information that is required.

Thank you !
Wes

Close
